# Dengue transmission dynamics in China’s border regions adjacent to Myanmar

**DOI:** 10.1186/s40249-026-01440-x

**Published:** 2026-04-07

**Authors:** Yanshu Ke, Hongjie Wei, Chunhai Luo, Jiahui Li, Fang Xie, Bingbing Wang, Hong Liu, Hongning Zhou, Qiuping Chen, Jia Rui, Tianmu Chen

**Affiliations:** 1 State Key Laboratory of Vaccines for Infectious Diseases, Xiang An Biomedicine Laboratory, National Innovation Platform for Industry-Education Integration in Vaccine Research, School of Public Health, Xiamen University, Xiamen, China; 2 Yunnan Innovative Team of Key Techniques for Vector-Borne Disease Control and Prevention, Yunnan Provincial Key Laboratory of Vector-Borne Diseases Control and Research, Yunan International Joint Laboratory of Tropical Infectious Diseases, Yunnan International Joint Laboratory of Vector Biology and Control, Yunnan Institute of Parasitic Diseases, Pu’er, 665000 Yunnan China; 3 Department of Epidemiology and Health Statistics, Xiangya School of Public Health, Central South University, Changsha City, Hunan Province China; 4 Faculty of Innovation Engineering, Macau University of Science and Technology, Taipa, Macau Special Administrative Region China

**Keywords:** Dengue fever, Vector-borne infectious diseases, Mathematical model, Tansmission dynamics

## Abstract

**Background:**

Dengue fever is a major mosquito-borne disease that is spreading rapidly from endemic regions to previously low-incidence regions. The regions at the interface between Southeast Asia and China represent critical hotspots for the cross-border and subsequent local transmission of dengue fever. In this study, a cross-population transmission model was constructed to characterize the transmission dynamics in China's border regions adjacent to Myanmar.

**Methods:**

Dengue case data from the dengue fever case reporting system were collected for Dehong Dai and Jingpo Autonomous Prefecture (DH) and Lincang City (LC), which are situated along the China-Myanmar border in southwestern China (2014–2023). We analyzed spatiotemporal patterns of dengue transmission and developed a transmission dynamics model that accounts for vertical transmission in mosquitoes to quantify transmission dynamics at three levels: the overall disease system, mosquito-to-human, and human-to-mosquito. Model parameters were estimated using least-squares fitting to observed case data, and model performance was evaluated using the coefficient of determination (*R*^2^).

**Results:**

From 2014 to 2023, a total of 10,180 dengue cases were documented across two China's border prefectures adjacent to Myanmar, 83.1% of which were locally acquired infections. There were 7,893 cases reported in DH (906 international imports, 6,961 local) and 2,287 cases reported in LC (717 international imports, 1,497 local). Transmission exhibited pronounced seasonality, peaking between July and November, and a strong temporal correlation between imported and local cases was observed. By using a dynamic transmission model incorporating mosquito vertical transmission, we achieved statistically significant model fits (*P* < 0.001) and quantified temporal changes in transmissibility. During the rising phases of the outbreak, the overall transmissibility consistently exceeded 1. Analysis of directional transmission revealed a marked temporal shift: human-to-mosquito transmissibility was predominant in earlier outbreak years (2017: 1–5 in DH, 10–15 in LC), whereas mosquito-to-human transmissibility has increased substantially in recent years (2023: 5–15 in DH, 10–20 in LC). Sensitivity analyses demonstrated that while overall transmissibility estimates remained robust across different initial mosquito population assumptions, the directional transmission components were sensitive to the ratio of exposed to infected mosquitoes, reflecting the inherent identifiability challenges of the model in the absence of entomological surveillance data.

**Conclusions:**

Our findings reveal the dengue transmission dynamics in China's border regions adjacent to Myanmar over the 2014–2023 study period. Our results underscore the necessity of integrating entomological monitoring with case-based surveillance and support enhanced cross-border coordination for effective outbreak prevention in high-risk frontier zones.

**Graphical Abstract:**

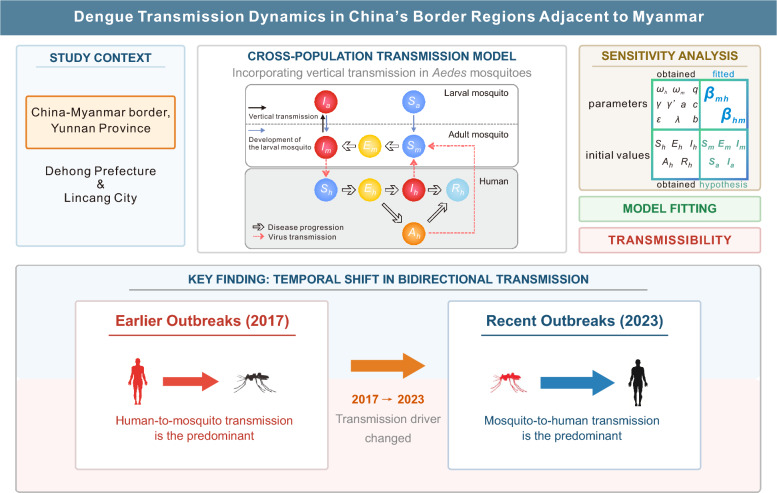

**Supplementary Information:**

The online version contains supplementary material available at 10.1186/s40249-026-01440-x.

## Background

Dengue fever is an acute mosquito-borne infectious disease caused by the dengue virus and has become a major public health challenge globally, particularly in tropical and subtropical regions [1]. In recent years, global climate change, the resumption of international travel and intensified cross-border population movement have exacerbated the risk of dengue transmission [2, 3]. In China, imported cases remain a driving factor for local dengue outbreaks [4], with a high incidence in the Lancang-Mekong River Basin countries, coupled with frequent cross-border population movements and interactions among countries in the border region, posing a significant threat to the neighboring Yunnan Province [5]. Given that Yunnan’s climate is suitable for *Aedes* survival for much of the year [6, 7], Yunnan has therefore been regarded as the ‘bridgehead’ for the transmission of dengue fever into China [4]. Dehong Dai and Jingpo Autonomous Prefecture (DH) and Lincang City (LC), both located on the China–Myanmar border, have a subtropical monsoon climate conducive to the breeding and survival of *Aedes*. As a result of this climate and the frequent migration of individuals from Southeast Asian countries, these regions exhibit higher dengue fever incidence rates within the province [8, 9].

Previous studies on dengue transmission along the China-Myanmar border have primarily focused on epidemiological surveillance and risk factor identification [10, 11]. Spatiotemporal analyses have revealed distinct seasonal patterns and cross-border importation dynamics in these border areas [12]. However, the underlying transmission mechanisms remain inadequately characterized. Mathematical models have proven valuable in dengue research worldwide, with temperature-driven models being used to assess the roles of climate and imported cases in local outbreaks [13], and metapopulation network models being used to examine transmission patterns across urban clusters [14]. Despite these advances, current assessments of transmissibility are mostly limited to the entire disease system and lack independent analyses of the two critical pathways: human-to-mosquito and mosquito-to-human transmission [15, 16].

Here we characterize dengue transmission dynamics in the China-Myanmar border region using a cross-population transmission model that incorporates the vertical transmission patterns of the dengue virus in *Aedes* mosquitoes. We analyze spatiotemporal patterns of dengue cases from 2014 to 2023 and quantified transmissibility across epidemic phases. Crucially, unlike conventional approaches that rely on overall transmissibility estimates, we separately quantified human-to-mosquito and mosquito-to-human transmission, thereby revealing the directional components that drive dengue spread in this border hotspot.

## Methods

### Study design

**Fig. 1:**
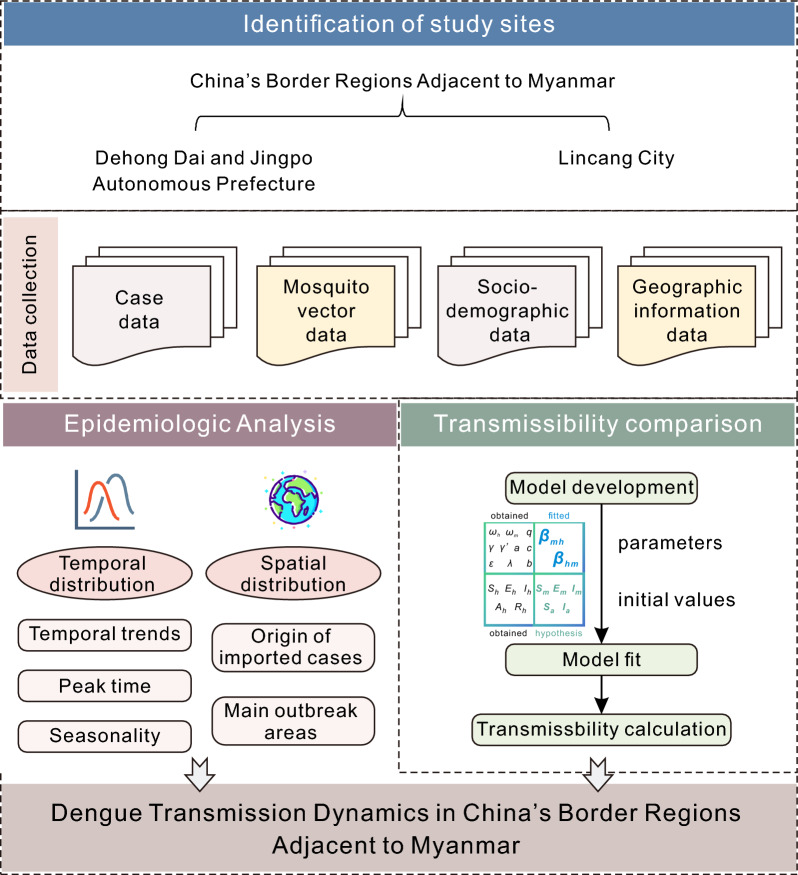
Schematic diagram of the study design

In this study, the transmission dynamics of dengue fever along the China-Myanmar border were investigated. We integrated case reports, mosquito surveillance, sociodemographic and geographical data to perform spatiotemporal analyses. Using a transmission dynamics model and model fitting, we quantified the transmissibility, including its human-to-mosquito and mosquito-to-human components. Analysis of transmission dynamics at the China-Myanmar border provides critical insights into dengue transmission mechanisms and informs evidence-based control strategies. The study framework is shown in Fig. 1.

### Data on dengue cases

The research data on dengue fever in DH and LC were obtained from the dengue fever case reporting system established by the Yunnan Institute of Parasitic Diseases. This dataset includes basic demographic information (e.g., sex, age, place of birth, current residential address according to national standards, and population classification) and disease diagnosis information (e.g., onset date, diagnosis date, and case classification) for each patient. The case discovery and reporting in both DH and LC followed the standards set in the *Yunnan Province Dengue Fever Surveillance and Control Plan (2023 Edition)*. On the basis of origin of the cases, the dengue cases were categorized into imported cases and local cases. Imported cases refer to those who had traveled to countries or regions with dengue fever outbreaks within 14 days before symptom onset, whereas local cases refer to those who had not left their city of residence within 14 days before symptom onset [17]. We included all dengue fever cases reported between January 1, 2014, and December 31, 2023, in the analysis, separately assessing the temporal and spatial distributions of imported and local dengue cases in DH and LC. In parallel, Breteau Index (BI) data were collected for both DH and LC from the routine *Aedes* vector surveillance system operated by the Yunnan Institute of Parasitic Diseases, in which household and container inspections were conducted according to guidelines established in the *Surveillance Program for Aedes Vectors of Dengue in China.*

### Transmission dynamic model of dengue fever

**Fig. 2:**
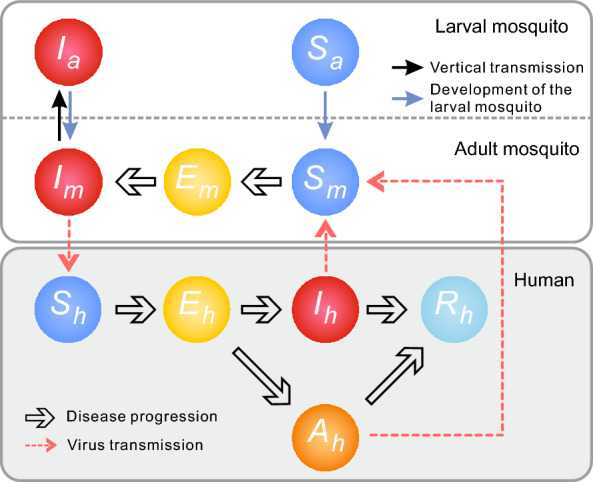
Framework of the dengue fever cross-population transmission dynamics model between *Aedes* and Humans

On the basis of the epidemiological characteristics and the natural disease course of dengue transmission, we developed a cross-population transmission dynamics model for *Aedes* and humans that incorporates the vertical transmission of dengue virus in *Aedes* (Fig. 2). In this model, the human population was divided into five compartments: susceptible individuals (*S*_*h*_), exposed individuals (*E*_*h*_), symptomatic infected individuals (*I*_*h*_), asymptomatic infected individuals (*A*_*h*_), and recovered individuals (*R*_*h*_). We divided the aquatic stage of *Aedes* into two compartments: susceptible larvae (*S*_*a*_) and infected larvae (*I*_*a*_). Adult mosquitoes were categorized into three compartments: susceptible adults (*S*_*m*_), exposed adults (*E*_*m*_), and infected adults (*I*_*m*_).

The model was developed with the following assumptions:

1. During the epidemic, the natural birth and death rates are relatively low compared with the total population size and can therefore be neglected.
2. The transmission rate coefficients from *I*_*m*_ to *S*_*h*_ and from *I*_*h*_/*A*_*h*_ to *S*_*m*_ are *β*_*mh*_ and *β*_*hm*_, respectively. The numbers of newly infected humans and mosquitoes at time *t* are *β*_*mh*_*S*_*h*_*I*_*m*_ and *β*_*hm*_*S*_*m*_(*I*_*h*_ + *A*_*h*_).
3. The proportion of asymptomatic infected individuals is *q*, and the mean intrinsic incubation period is $\frac{1}{{\omega }_{h}}$. Therefore, at time *t*, the number of individuals progressing from *E*_*h*_ to *I*_*h*_ and *A*_*h*_ is (1—*q*)*ω*_*h*_*E*_*h*_ and *qω*_*h*_*E*_*h*_, respectively.
4. Symptomatic individuals *I*_*h*_ recover at rate *γ*, and asymptomatic individuals *A*_*h*_ recover at rate *γ’*. At time *t*, *γI*_*h*_ and *γ’A*_*h*_ individuals transition to *R*_*h*_.
5. Exposed mosquitoes *E*_*m*_ progress to infected mosquitoes *I*_*m*_ after a mean extrinsic incubation period of $\frac{1}{{\omega }_{m}}$. At time *t*, the number of *E*_*m*_ that transition to *I*_*m*_ is *ω*_*m*_*E*_*m*_.
6. The proportion of infected adult female mosquitoes that directly transfer the virus to their offspring is *n*. Thus, at time *t*, the number of *I*_*a*_ generated through vertical transmission from *I*_*m*_ is *acnI*_*m*_.
7. Aquatic-stage mosquitoes progress to adults at rate *λ*. Therefore, at time *t*, the numbers of *S*_*a*_ and *I*_*a*_ that progress to *S*_*m*_ and *I*_*m*_ are *λS*_*a*_ and *λI*_*a*_, respectively.

The differential equations for the transmission dynamics model are as follows:

$$\frac{{dS_{a} }}{dt} = \alpha c\left( {N_{m} - nI_{m} } \right) - \lambda S_{a}$$

$$\frac{{dI_{a} }}{dt} = \alpha cnI_{m} - \lambda I_{a}$$

$$\frac{{dS_{m} }}{dt} = \lambda S_{a} - \frac{{\beta_{hm} S_{m} \left( {A_{h} + I_{h} } \right)}}{{N_{h} }} - bS_{m}$$

$$\frac{{dE_{m} }}{dt} = \frac{{\beta_{hm} S_{m} \left( {A_{h} + I_{h} } \right)}}{{N_{h} }} - \omega_{m} E_{m} - bE_{m}$$

$$\frac{{dI_{m} }}{dt} = \lambda I_{a} + \omega_{m} E_{m} - bI_{m}$$

$$\frac{{dS_{h} }}{dt} = - \frac{{\beta_{mh} S_{h} I_{m} }}{{N_{h} }}$$

$$\frac{{dE_{h} }}{dt} = \frac{{\beta_{mh} S_{h} I_{m} }}{{N_{h} }} - \omega_{h} E_{h}$$

$$\frac{{dI_{h} }}{dt} = \left( {1 - q} \right)\omega_{h} E_{h} - \gamma I_{h}$$

$$\frac{{dA_{h} }}{dt} = q\omega_{h} E_{h} - \gamma^{\prime}A_{h}$$

$$\frac{{dR_{h} }}{dt} = \gamma I_{h} + \gamma^{\prime}A_{h}$$

### Parameter estimation

The definitions and values of the parameters in the model are shown in Table 1. The incubation period of the dengue virus in humans is generally 4–10 days [18], and an average value of 7 days was used in the model, with *ω*_*h*_ = 0.1429. After entering the *Aedes* mosquito, the dengue virus typically requires an extrinsic incubation period of 8–12 days to replicate in the mosquito and become transmittable to a new host [19, 20]. Therefore, ab average value of 10 days was used in the model, with *ω*_*m*_ = 0.1000. The proportion of asymptomatic infections is usually 68.75% [21]; thus, *q* = 0.6875. The infectious period of dengue patients after infection is 3–14 days [19, 22], and we used 7 days in the simulation, with *γ* = *γ’* = 0.1429. In the model, the daily natural birth and death rates of mosquitoes were both 0.0714 [20, 23]. On the basis of life cycle of *Aedes*, it takes approximately 7–10 days for larvae to develop into adults [24]; thus, the average emergence rate in the model *λ* was 0.1176. The vertical transmission rate of the dengue virus ranges from 1.4% to 17.4% [25]; thus, a value of 0.1000 was used in the model. Referring to published studies [19, 26, 27], a seasonal parameter *c* was used in the model, which was, simulated using a trigonometric function:

$$c = \frac{{cos\left[ {\frac{{2\pi \left( {t - \tau } \right)}}{T}} \right] + 1}}{2}$$

in which *τ* represents the time to peak of the epidemic, *T* is the total number of days in the disease cycle, and *c* ranges from 0 to 1. Given the similar seasonality between the BI and case numbers at both study sites, we determined *τ* on the basis of the timing of annual case peaks (Supplementary Fig. 1.3–1.4).

**Table 1:** Parameter description and values of the mathematical model

Parameters	Definition	Unite	Value	Range	Methods
*β*_*mh*_	The transmission rate coefficient from mosquitoes to humans	1	–	≥ 0	Model fitting
*β*_*hm*_	The transmission rate coefficient from humans to mosquitoes	1	–	≥ 0	Model fitting
*ω*_*h*_	Progression rate from latency to disease in humans	Day^−1^	0.1429	0.1000–0.2500	Reference [18]
*ω*_*m*_	Progression rate from latency to disease in mosquitoes	Day^−1^	0.1000	0.0833–0.1250	References [19, 20]
*q*	Proportion of asymptomatic infections	1	0.6875	0–1	Reference [21]
*γ*	Recovery rate of symptomatic infected individuals	Day^−1^	0.1429	0.0714–0.3333	References [19, 22]
*γ’*	Recovery rate of asymptomatic infected individuals	Day^−1^	0.1429	0.0714–0.3333	References [20, 23]
*a*	Natural birth rate of *Aedes*	Day^−1^	0.0714	0.0200–0.2500	References [20, 23]
*c*	Seasonal parameter	1	–	0–1	Data collection
*n*	Proportion of vertical transmission	1	0.1000	0.0140–0.1740	Reference [25]
*λ*	Average emergence rate	Day^−1^	0.1176	0.1000–0.1429	Reference [24]
*b*	Natural death rate of *Aedes*	Day^−1^	0.0714	–	References [20, 23]
*N*_*h*_	Population size of humans	Individuals	–	–	Statistical Yearbook of Yunnan Province
*N*_*m*_	Population size of *Aedes*	Individuals	–	–	Hypothesis

*β*_*mh*_ human biting rate × infection probability per bite, *β*_*hm*_ mosquito biting rate × infection probability per bite

-: Not applicable

### Model fitting

Parameter estimation was performed using the lmfit package in Python 3.12.4 (Python Software Foundation, Wilmington, Delaware, USA). The ODEs were solved with RK45. Only two parameters were estimated, the mosquito-to-human and human-to-mosquito transmission rates (*β*_*mh*_, *β*_*hm*_); all other rates and initial conditions were fixed from data or literature. Model fit was summarized by mean squared error (MSE), coefficient of determination (*R*^2^), and mean absolute error (MAE).

### Quantification of the transmissibility of dengue fever

The basic reproduction number (*R*_0_) refers to the average number of secondary cases generated by a single infectious individual in a fully susceptible population and is used to quantify the transmissibility of an infectious disease [28]. If *R*_0_ > 1, the disease can cause an epidemic; if *R*_0_ < 1, the disease is unlikely to spread. However, *R*_0_ only represents the initial transmissibility of a disease. Since our model incorporated time-varying seasonal parameters, we used the time-varying reproduction number (*R*_*t*_) instead of *R*_0_. *R*_*t*_ is the average number of secondary cases produced by one primary case at time *t* during the epidemic.

For the entire dengue fever transmission system, *R*_*t*_ was calculated using the next-generation matrix method. The calculation process is as follows:

$${ \mathcal{F}} = \left( {\begin{array}{*{20}c} {\frac{{\beta_{hm} S_{m} \left( {I_{h} + A_{h} } \right)}}{{N_{h} }}} \\ 0 \\ {\frac{{\beta_{mh} S_{h} I_{m} }}{{N_{h} }}} \\ 0 \\ 0 \\ \end{array} } \right)$$

$${ \mathcal{V}} = \left( {\begin{array}{*{20}c} {\omega_{m} E_{m} + bE_{m} } \\ { - \lambda I_{a} - \omega_{m} E_{m} + bI_{m} } \\ {\omega_{h} E_{h} } \\ { - \left( {1 - q)\omega_{h} E_{h} + \gamma I_{h} } \right.} \\ { - q\omega_{h} E_{h} + \gamma^{\prime}A_{h} } \\ \end{array} } \right)$$

$$F = \left( {\begin{array}{*{20}c} 0 & 0 & 0 & {\frac{{\beta_{hm} S_{m} }}{{N_{h} }}} & {\frac{{\beta_{hm} S_{m} }}{{N_{h} }}} \\ 0 & 0 & 0 & 0 & 0 \\ 0 & {\frac{{\beta_{mh} S_{h} }}{{N_{h} }}} & 0 & 0 & 0 \\ 0 & 0 & 0 & 0 & 0 \\ 0 & 0 & 0 & 0 & 0 \\ \end{array} } \right)$$

$$V = \left( {\begin{array}{*{20}c} {\omega_{m} + b} & 0 & 0 & 0 & 0 \\ { - \omega_{m} } & b & 0 & 0 & 0 \\ 0 & 0 & {\omega_{h} } & 0 & 0 \\ 0 & 0 & { - \left( {1 - q} \right)\omega_{h} } & \gamma & 0 \\ 0 & 0 & { - q\omega_{h} } & 0 & {\gamma^{\prime}} \\ \end{array} } \right)$$

The disease-free equilibrium is as follows:

$$E_{0} = \left( {S_{a}^{0} , I_{a}^{0} , S_{m}^{0} , E_{m}^{0} , I_{m}^{0} , S_{h}^{0} , E_{h}^{0} , I_{h}^{0} , A_{h}^{0} } \right) = \left( {\frac{{acN_{m} }}{\lambda }, 0,\frac{{acN_{m} }}{b}, 0, 0, N_{h} , 0, 0, 0} \right)$$

By substitution, we obtain the following:

$$R_{t} = \rho \left( {FV^{ - 1} \left( {E_{0} } \right)} \right) = \sqrt {\frac{{\beta_{hm} acN_{m} \left( {\gamma^{\prime} - \gamma^{\prime}q + \gamma q} \right)}}{{b\gamma \gamma ^{\prime}N_{h} }}} \times \sqrt {\frac{{\beta_{mh} \omega_{m} }}{{b\left( {\omega_{m} + b} \right)}}}$$

Furthermore, we decompose *R*_*t*_ into human-to-mosquito and mosquito-to-human transmission components to assess the transmissibility of each pathway. The formula is as follows:

$$R_{{t\left( {hm} \right)}} = \frac{{\beta_{hm} acN_{m} \left( {\gamma^{\prime} - \gamma^{\prime}q + \gamma q} \right)}}{{b\gamma \gamma ^{\prime}N_{h} }}$$

$$R_{{t\left( {mh} \right)}} = \frac{{\beta_{mh} \omega_{m} }}{{b\left( {\omega_{m} + b} \right)}}$$

*R*_*t*(*hm*)_ represents human-to-mosquito transmissibility, and *R*_*t*(*mh*)_ represents mosquito-to-human transmissibility.

### Sensitivity analysis

When fitting the cross-population transmission dynamics model for dengue fever, the initial values of the human compartments were calculated using Statistical Yearbooks, case reporting systems, and parameter settings in the model. However, the lack of monitoring data on the number of *Aedes* made it difficult to obtain accurate estimates of *Aedes,* and we conducted sensitivity analyses by varying the initial mosquito population parameters. Specifically, the total adult mosquito population (*N*_*m*_) was set at 1, 5, 10, 15, and 20 times the human population size, with the number of infected adult mosquitoes set at 10. The number of exposed adult mosquitoes was assumed to be 1–5 times the number of infected adult mosquitoes, and the number of larval compartments was calculated on the basis of the inflow rate defined in the model. A total of 25 hypothetical scenarios were constructed, as shown in Table 2.The model was fitted to dengue fever cases in DH and LC from 2014 to 2023. Years with higher case numbers and better fitting results were selected to calculate the transmissibility.

**Table 2:** Initial values for sensitivity analysis scenarios of the dengue fever transmission model

Scenarios	*S*_*a*0_	*I*_*a*0_	*S*_*m*0_	*E*_*m*0_	*I*_*m*0_
1	*ac*_0_(*N*_*h*_-*nI*_*m*0_)	*ac*_0_*nI*_*m*0_	*N*_*h*_-*E*_*m*0_-*I*_*m*0_	*I*_*m*0_	10
2				2*I*_*m*0_	
3				3*I*_*m*0_	
4				4*I*_*m*0_	
5				5*I*_*m*0_	
6	*ac*_0_(*N*_h_*5-*nI*_*m*0_)	*ac*_0_*nI*_*m*0_	*N*_h_*5-*E*_*m*0_-*I*_*m*0_	*I*_*m*0_	10
7				2*I*_*m*0_	
8				3*I*_*m*0_	
9				4*I*_*m*0_	
10				5*I*_*m*0_	
11	*ac*_0_(*N*_h_*10-*nI*_*m*0_)	*ac*_0_*nI*_*m*0_	*N*_h_*10-*E*_*m*0_-*I*_*m*0_	*I*_*m*0_	10
12				2*I*_*m*0_	
13				3*I*_*m*0_	
14				4*I*_*m*0_	
15				5*I*_*m*0_	
16	*ac*_0_(*N*_h_*15-*nI*_*m*0_)	*ac*_0_*nI*_*m*0_	*N*_h_*15-*E*_*m*0_-*I*_*m*0_	*I*_*m*0_	10
17				2*I*_*m*0_	
18				3*I*_*m*0_	
19				4*I*_*m*0_	
20				5*I*_*m*0_	
21	*ac*_0_(*N*_h_*20-*nI*_*m*0_)	*ac*_0_*nI*_*m*0_	*N*_h_*20-*E*_*m*0_-*I*_*m*0_	*I*_*m*0_	10
22				2*I*_*m*0_	
23				3*I*_*m*0_	
24				4*I*_*m*0_	
25				5*I*_*m*0_	

*S*_*a*0_, *I*_*a*0,_ *S*_*m*0_, *E*_*m*0,_ and *I*_*m*0_ represent the initial values of the susceptible larvae, infected larvae, susceptible adults, exposed adults, and infected adults compartments, respectively. *c*_0_ denotes the value of the seasonal parameter at the beginning of each each annual fitting

### Uncertainty analysis

To explicitly account for uncertainty in poorly identified biological and entomological parameters, we implemented a Monte Carlo-based uncertainty propagation framework. Instead of treating all the parameters as fixed point estimates, we sampled selected parameters from biologically plausible ranges and propagated this uncertainty through the deterministic transmission model. The parameter ranges were defined according to the values reported in Table 1, which summarize the ranges documented in the literature.

For each scenario and time period, we generated 5000 Monte Carlo realizations by independently sampling the selected parameters from uniform distributions over their prescribed ranges and recomputing the corresponding transmission metrics. This procedure yields empirical distributions rather than single-point estimates. We summarized these distributions using medians and percentile-based uncertainty intervals and visualized them using violin plots. All downstream analyses were based on these distributional summaries (Supplementary Fig. 5.1–5.36).

## Results

In this study, the spatiotemporal distribution characteristics of dengue fever in China's border regions adjacent to Myanmar from 2014 to 2023 were analyzed, and a cross-population transmission dynamics model incorporating vertical transmission in *Aedes* was constructed to quantify transmissibility. Through mathematical modeling, we separately derived human-to-mosquito and mosquito-to-human transmissibility, revealing the complexity of dengue transmission mechanisms in border regions.

### Epidemiological characteristics of dengue fever

**Fig. 3:**
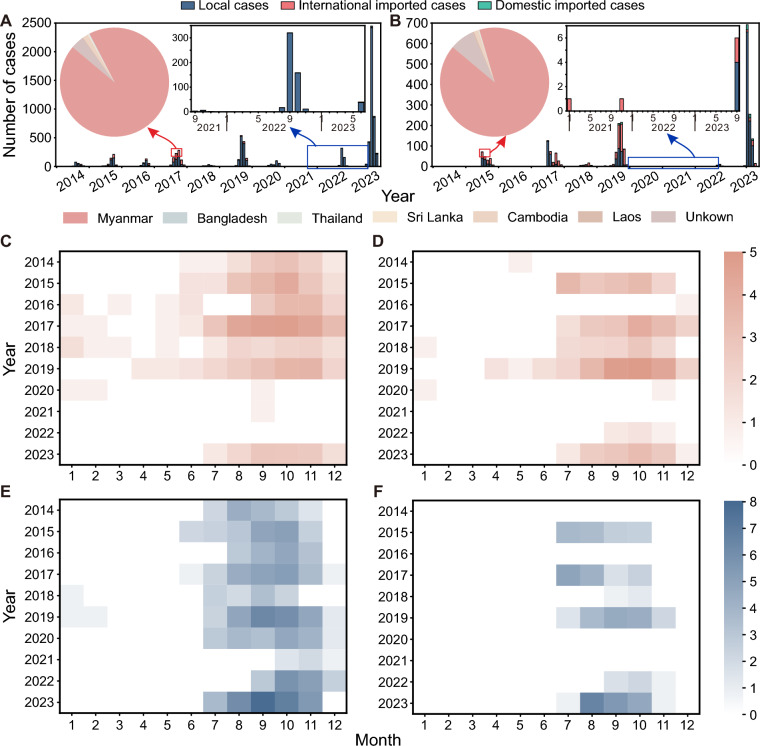
Temporal distribution of dengue fever cases in Dehong Prefecture and Lincang City from 2014 to 2023. **A**, **B** Monthly and annual distributions of dengue fever cases in DH and LC, Blue represents local cases, pink represents international imported cases, and green represents domestic imported cases. Pie charts indicate the proportion of international imported cases by source country. **C**, **D** Seasonal distribution of international imported cases in DH and LC. **E**, **F** Seasonal distribution of local cases in DH and LC. Owing to significant variations in dengue case numbers across different years, the data in **C**–**F** were normalized using a natural logarithmic transformation (ln(cases) + 1). *DH* Dehong Prefecture, *LC* Lincang City

Dengue fever outbreaks in these regions exhibit significant spatial heterogeneity, temporal fluctuations, and seasonal characteristics. From 2014 to 2023, a total of 7,893 dengue fever cases were reported in DH, including 906 international imported cases (hereinafter referred to as imported cases), 26 domestic imported cases, and 6,961 local cases (Fig. 3A). In LC, 2,287 dengue cases were reported, including 717 imported cases, 73 domestic imported cases, and 1,497 local cases (Fig. 3B). Dengue fever outbreaks in DH and LC exhibited a sawtooth-like upward trend, with higher case numbers in odd-numbered years. In DH, the number of imported cases peaked in 2017, while in 2023, the number of local cases peaked. Between 2020 and 2022, the number of imported cases in DH remained below 5, with local cases decreasing to 10 in 2021 but unexpectedly increasing to 506 in 2022 despite zero imports. In LC, the highest number of imported cases occurred in 2019, but this high number of imported cases did not lead to a local outbreak. Instead, the peak number of local cases occurred in 2023. In DH, the proportion of local cases exceeded 50% every year, whereas in LC, local cases accounted for more than half of the total cases only in 2017, 2022, and 2023. In terms of the sources of imported cases, Myanmar was the primary source country for both regions from 2014 to 2023. Both regions showed pronounced dengue seasonality during July–November. DH exhibited synchronous September–October peaks for both imported and local cases (Fig. 3C and E), while LC displayed a slightly shorter local transmission window from July to October for local cases compared to that of imported cases, which extended through November (Fig. 3D and F).

**Fig. 4:**
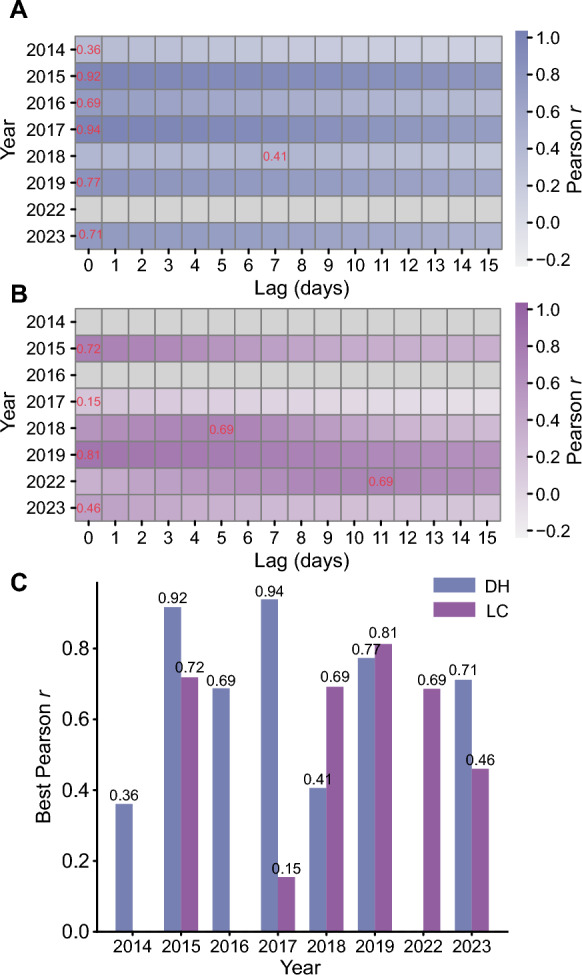
Lagged correlation between imported and local dengue cases in Dehong Prefecture and Lincang City from 2014 to 2023. Cases from 2020 to 2021 were excluded from the analysis because of insufficient case numbers. **A**, **B** Heatmaps display the Pearson correlation coefficients (*r*) between imported and local cases at different lag days (0–15) for DH and LC. Red numbers indicate the maximum significant positive correlation for each year. **C** Bar plot summarizing the best lag-specific correlation (maximum *r*) per year in each region, enabling interannual and interregional comparisons. *DH* Dehong Prefecture, *LC* Lincang City

In both DH and LC, imported and local dengue cases generally exhibited strong temporal correlations across years. Temporal correlation analysis revealed distinct interannual patterns in the association between imported and local dengue cases. In DH, the peak lagged correlation coefficient (*r*) exceeded 0.9 in 2015 and 2017, and remained above 0.5 except 2014 and 2018 (Fig. 4A and C). LC also maintained peak *r* values above 0.5 in most years (Fig. 4B and C).

Spatially, all five counties in DH had reported dengue cases, with Ruili City emerging as the epidemic epicenter, accounting for 68.0% of the imported cases and 96.6% of the local cases. In LC, all eight counties had reported imported cases, and seven counties, except Shuangjiang Lahu, Va, Blang, and Dai Autonomous County, had reported local cases. Zhenkang County (339, 47.3%) and Gengma Dai and Va Autonomous County (338, 47.1%) had the highest numbers of imported cases, while Gengma Dai and Va Autonomous County (1346, 89.9%) had the greatest number of local cases (Table 3).

**Table 3:** Spatial distribution of dengue fever in Dehong Prefecture and Lincang City

Region	Local cases	Imported cases
Dehong Prefecture		
Ruili City	6,722	616
Mang City	102	39
Lianghe County	11	6
Yingjiang County	35	176
Longchuan County	91	69
Lincang City		
Linxiang District	4	34
Fengqing County	7	5
Yun County	5	5
Yongde County	12	2
Zhenkang County	339	72
Shuangjiang Lahu, Va, Blang and Dai Autonomous County	2	0
Gengma Dai and Va Autonomous County	338	1,346
Cangyuan Va Autonomous County	10	33

### Model results

**Fig. 5:**
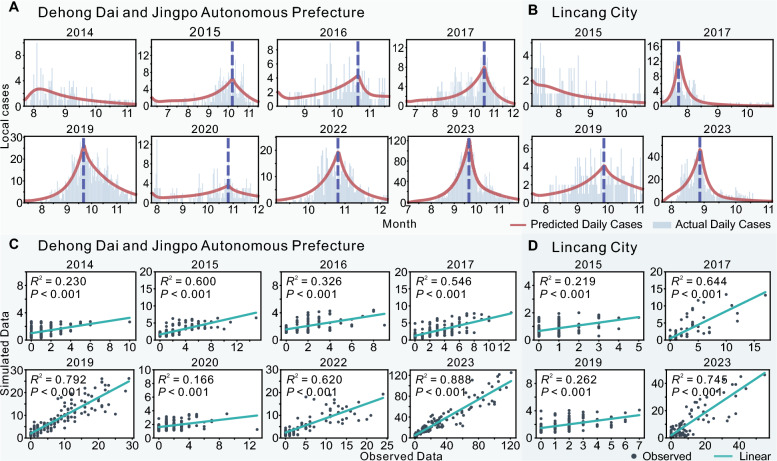
Model fitting results for Scenario 13. **A**, **B** Fitting results of dengue fever outbreaks in DH and LC. The fitted segmentation points between the ascending and descending phases of the epidemic are indicated by purple dashed lines. **C**, **D** Model fitting performance of dengue fever outbreaks in DH and LC. *DH* Dehong Prefecture, *LC* Lincang City

In this study, years with a high number of local cases (> 60) in the two regions from 2014 to 2023 were selected for model fitting. To analyze the epidemic trends in greater detail, the dengue outbreaks (except for DH in 2014 and LC in 2015) were divided into two phases: the rising phase and the declining phase. The fitting results for the 25 scenarios in each year were in good agreement with the actual case numbers, with *P* < 0.001, indicating statistical significance (Fig. 5 and Supplementary Fig. 2.1–2.24).

### Results of dengue fever transmissibility analysis

On the basis of the model fitting results, the years with more reported cases were selected in this study. Owing to the presence of seasonal parameters, the transmissibility of dengue fever varied over time (Supplementary Fig. 3.1–3.12). To facilitate comparison, we summarized *R*_*t*_ using its median value over each epidemic phase. The results revealed that the *R*_*t*_ values were consistently greater than 1 during the epidemic-rising phase, but gradually decreased to less than 1 during the declining phase, which is consistent with the characteristics of epidemic progression. In this study, we focused more on the results during the rising phase when *R*_*t*_ > 1.

**Fig. 6:**
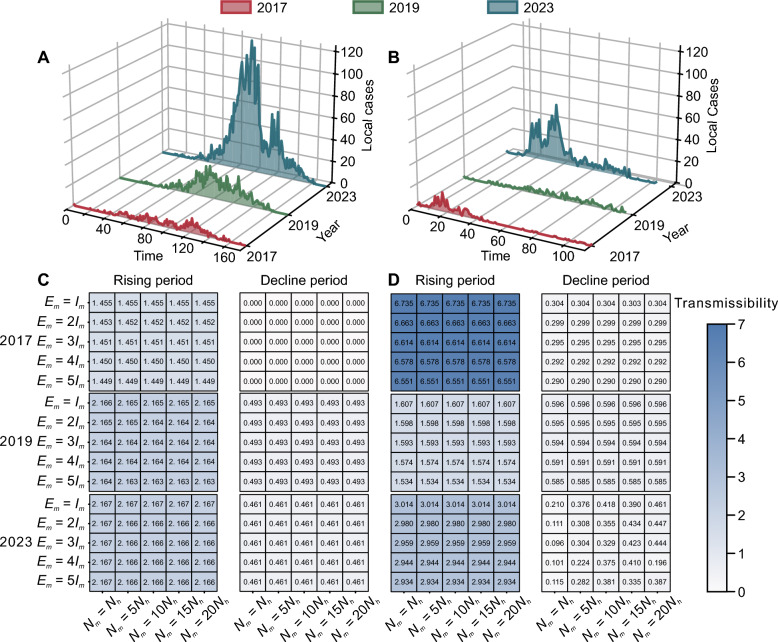
Calculation results of dengue fever transmissibility. **A**, **B** Number of local dengue cases in DH and LC for years with higher case numbers and better fitting performance. **C**, **D** Median *R*_*t*_ during the rising and declining phases of dengue outbreaks in DH and LC for years with higher case numbers and better fitting performance. *N*_*m*_ represents the total number of adult mosquitoes, *N*_*h*_ represents the total human population, *E*_*m*_ represents exposed adult mosquitoes, and *I*_*m*_ represents infected adult mosquitoes. *DH* Dehong Prefecture, *LC* Lincang City

We analyzed the temporal patterns of dengue transmission in the China-Myanmar border region during years with substantial outbreak activity (Fig. 6A and B). The 2023 outbreak exhibited pronounced epidemic peaks, with approximately 120 local cases in DH and 50 cases in LC at maximum intensity, substantially exceeding the case burdens observed in 2017 and 2019. During the 2017 outbreak, the *R*_*t*_ values ranged from 1.45 to 1.46 in DH and from 6.55 to 6.74 in LC across the different scenarios. By 2019, the *R*_*t*_ estimates reached 2.16–2.17 in DH and 1.53–1.61 in LC across different scenarios. The *R*_*t*_ values of 2023 outbreak were maintained at 2.17 in DH, while LC exhibited elevated estimates ranging from 2.86 to 3.01, indicating sustained transmission potential in both prefectures in recent years (Fig. 6C and D). Sensitivity analyses revealed that the *R*_*t*_ estimates remained relatively stable across varying mosquito-to-human population ratios and different initial conditions for exposed and infected mosquitoes during the rising phase.

**Fig. 7:**
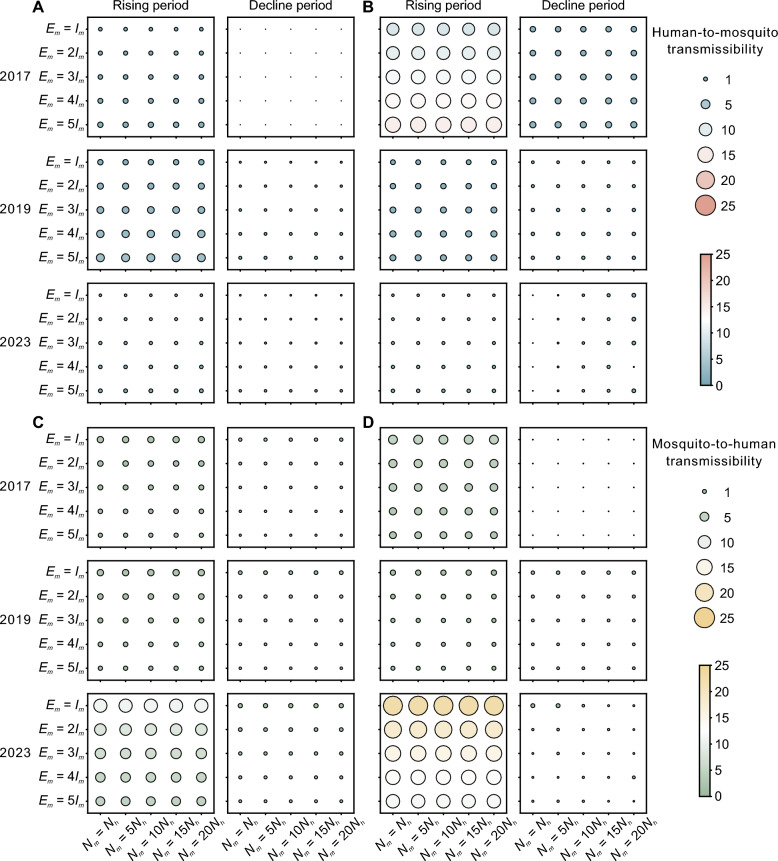
Calculation results of human-to-mosquito and mosquito-to-human transmissibility. **A**, **B** Median *R*_*t*(*hm*)_ during the rising and declining phases of dengue outbreaks in DH and LC for years with higher case numbers and better fitting performance. **C**, **D** Median *R*_*t*(*mh*)_ during the rising and declining phases of dengue outbreaks in DH and LC for years with higher case numbers and better fitting performance. *N*_*m*_ represents the total number of adult mosquitoes, *N*_*h*_ represents the total human population, *E*_*m*_ represents exposed adult mosquitoes, and *I*_*m*_ represents infected adult mosquitoes. *DH* Dehong Prefecture, *LC* Lincang City

With respect to human-to-mosquito transmissibility during the rising period (Fig. 7A and B), DH exhibited moderate *R*_*t*(*hm*)_ values of 1–2 in 2017, approximately 2–4 in 2019, and near-zero levels in 2023. In LC, high *R*_*t*(*hm*)_ values of of 10–15 were observed in 2017. Markedly reduced transmissibility were observed in 2019 and 2023 (approximately about 2 and near-zero, respectively). With respect to the mosquito-to-human direction (Fig. 7C and D), low *R*_*t*(*mh*)_ values (1–3) were maintained in both 2017 and 2019 in DH but increased to 5–10 in 2023. In LC, the *R*_*t*(*mh*)_ values were approximately 3–5 in 2017 and decreased to approximately 1 in 2019, but dramatically increased in 2023, with values reaching 10–20. Sensitivity analyses revealed that transmission dynamics were most responsive to variations in the ratio of exposed to infected mosquitoes (*E*_*m*_/*I*_*m*_) but showed limited sensitivity to total mosquito population size. Notably, human-to-mosquito and mosquito-to-human transmission exhibited opposite responses to changes: increasing *E*_*m*_/*I*_*m*_ ratios were associated with increased *R*_*t*(*hm*)_ but decreased *R*_*t*(*mh*)_, suggesting a trade-off in bidirectional transmission dynamics during outbreak progression.

## Discussion

Dengue fever has been recognized as one of the most significant global public health challenges [29]. Owing to its unique geographical location, Yunnan Province experiences frequent cross-border population movements, making it a high-risk area for dengue fever in China [30]. In this study, using 2014–2023 dengue fever case data from DH and LC, the spatiotemporal distribution characteristics of the outbreaks was analyzed and a mathematical model was constructed to evaluate the transmissibility of dengue fever. The China-Myanmar border region exhibited considerable interannual variability in transmissibility during epidemic phases, with relatively elevated values observed in certain years such as 2023 and 2017. Analysis of bidirectional transmission suggested temporal shifts in transmission patterns: human-to-mosquito transmission tended to be more prominent in earlier outbreaks, whereas mosquito-to-human transmission appeared to intensify in more recent years, reflecting dynamic changes in transmission mechanisms over time.

Our findings reveal distinctive spatiotemporal characteristics of dengue transmission dynamics in the China's border regions adjacent to Myanmar. The number of imported cases peaked from July to November, which coincided with the dengue transmission season in Southeast Asia and the peak mosquito activity [31, 32]. This temporal alignment between importation risk and local transmission suitability is critical for outbreak initiation, as imported cases typically undergo local transmission with lag times of 0–3 months under favorable conditions [33]. The strength of lagged temporal associations varied across border prefectures, reflecting the interplay between importation pressure and local amplification factors. The associations between imported and local cases were stronger in border areas with extensive cross-border mobility, intensive trade, lower altitudes, and warmer climates [34–37]. Notably, despite the significant dengue outbreak in Southeast Asia during 2022 [38, 39], no imported cases were reported in DH during this period. This absence may reflect surveillance limitations, border control measures, or transient undetected infections. The potential role of cross-border mosquito dispersal in maintaining cryptic transmission also warrants attention, as *Aedes* mosquitoes can disperse several hundred meters daily independent of human movement [40].

The mathematical model effectively captured dengue epidemic dynamics in the China-Myanmar border regions with a statistically significant fitting performance. By separately quantifying human-to-mosquito and mosquito-to-human transmission pathways, the model revealed bidirectional transmission patterns that conventional system-level *R*_*t*_ estimates would mask, providing mechanistic insights into dengue transmission processes in border settings. The substantial interannual variability in transmissibility reflects the dynamic nature of dengue transmission at the China-Myanmar border, where epidemic intensity is shaped by multiple interacting factors including imported case introductions, local vector population dynamics, environmental conditions, and accumulated population immunity [14, 41]. The heterogeneity observed across different years and locations underscores the complexity of predicting dengue risk in border regions with continuous cross-border mobility and viral importation. The contrasting temporal trajectories of bidirectional transmission represent the most notable finding. The progressive decline in human-to-mosquito transmissibility alongside the dramatic increase in mosquito-to-human transmission by 2023 suggests a fundamental shift in transmission dynamics, with infected mosquito populations becoming the predominant driver. Several mechanisms may contribute to this pattern. First, the co-circulation of multiple dengue serotypes in border regions [42], combined with waning heterotypic immunity (approximately 2 years) [43], could create population immunity profiles that favor mosquito-driven amplification while reducing human infectiousness to mosquitoes. Second, vertical transmission in *Aedes* populations may sustain infected vectors across seasons, maintaining high mosquito infection prevalence independent of human case numbers [44, 45]. This shift highlights the importance of vector surveillance and control strategies that target infected mosquito populations and the need for molecular surveillance to detect potential serotype displacement or the emergence of strains with altered transmission characteristics.

The sensitivity analysis revealed that while the overall transmissibility estimate of dengue remained stable across scenarios, the directional transmission metrics, namely human-to-mosquito and mosquito-to-human transmissibility, exhibited a clear trade-off: they responded inversely to changes in the assumed initial ratio of exposed to infectious mosquitoes. This inverse relationship highlights a key uncertainty: in the absence of independent data on mosquito infection status, the model cannot uniquely determine the exact strength of each transmission direction. In practice, multiple combinations of human-to-mosquito and mosquito-to-human efficiency could reproduce the observed case curves. Therefore, the absolute values of *R*_*t*(*hm*)_ and *R*_*t*(*mh*)_ should be interpreted as plausible estimates within a constrained range rather than as precise measurements.

Several limitations warrant consideration. First, mechanistic models face inherent identifiability challenges, where different parameter combinations can produce similar epidemic curves. Second, given the lack of actual entomological surveillance data, we set initial mosquito population values on the basis of hypothetical scenarios rather than field observations, which may not fully capture the spatiotemporal dynamics of vector populations. Third, although case collection and reporting followed standardized protocols, potential variations in detection rates during epidemics were not accounted for in the model. To address these gaps and translate findings into actionable strategies, future efforts should prioritize integrated, field-based entomological surveillance to ground model parameters in empirical data, alongside molecular surveillance of circulating serotypes and vector species. Public health measures should strengthen cross-border coordination and focus on the early identification of infected individuals during high-risk periods to interrupt the human-mosquito transmission cycle. Implementing these complementary approaches will provide more robust scientific evidence for dengue prevention and control in Yunnan and other border regions of China.

## Conclusion

Our study reveals the dengue transmission dynamics in China's border regions adjacent to Myanmar between 2014 and 2023. By developing a transmission dynamics model that separates human-to-mosquito transmission and mosquito-to-human transmission, we identified a temporal shift in transmission dynamics, with mosquito-to-human transmission becoming the predominant driver in the later years of the study period. These findings underscore the necessity of integrating entomological surveillance with case-based monitoring and emphasize the importance of binational coordination for effective dengue control in border regions.

## Supplementary Information

Supplementary material 1.

## Data Availability

The Python code supporting the conclusions of this article is available in the GitHub repository (https://github.com/kys1223/dengue_model). The datasets used and analyzed during the current study are available from the corresponding author on reasonable request.

## Abbreviations

DH: Dehong Dai and Jingpo Autonomous Prefecture
LC: Lincang City
BI: Breteau Index
*R*_0_: Basic reproduction number
*R*_*t*_: Time-varying reproduction number
*R*^2^: Coefficient of determination
*R*_*t*__(__*mh*__)_: Mosquito-to-human transmissibility
*R*_*t*__(__*hm*__)_: Human-to-mosquito transmissibility
MSE: Mean Squared Error
MAE: Mean Absolute Error
