# Supplementary material for: Dengue transmission dynamics in China’s border regions adjacent to Myanmar
Source: Infect Dis Poverty. 2026 Apr 7;15:41. doi: 10.1186/s40249-026-01440-x (PMC13055008; doi:10.1186/s40249-026-01440-x)
Supplement: Supplementary file 1 — Supplementary material 1. [file 40249_2026_1440_MOESM1_ESM.docx]

**Supplementary information for**

Dengue transmission dynamics in China’s border regions adjacent to Myanmar

[1. Distribution of *Aedes* Mosquitoes 2](#_Toc20277)

[2. Model fitting results 4](#_Toc983)

[3. Dengue fever transmissibility *R_t_* for all scenarios 16](#_Toc15882)

[4. Dengue fever human-to-mosquito transmissibility *R_t_*_(_*_hm_*_)_ for all scenarios 23](#_Toc13720)

[5. Uncertainty analysis 30](#_Toc4398)

# Distribution of *Aedes* Mosquitoes

Ruili (Dehong) exhibited frequent and intense fluctuations in Breteau Index (BI), with repeated peaks and persistent high volatility, while Gengma (Lincang) showed a later upward trajectory, with a prominent peak over 40 in 2023 (Fig. 1.1). The annual average bar chart reveals that Ruili maintained higher average BI than Gengma from 2014 to 2021, with its BI peaking at 14.9 (2020) and 15.0 (2021), whereas Gengma’s BI was relatively low initially but surged to 14.6 in 2023, surpassing Ruili’s 8.8 that year, indicating divergent temporal trends and regional differences in *Aedes* mosquito abundance over the decade (Fig. 1.2).


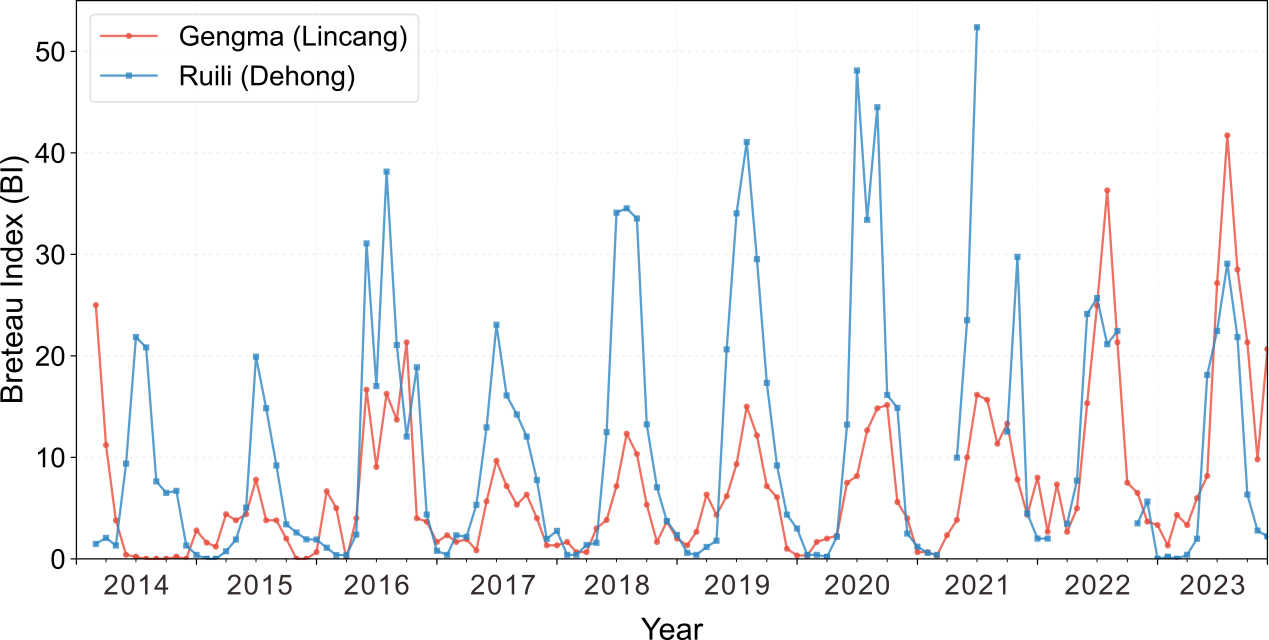


**Fig. 1.1 Monthly Trend of the Breteau Index, 2014–2023.**

**
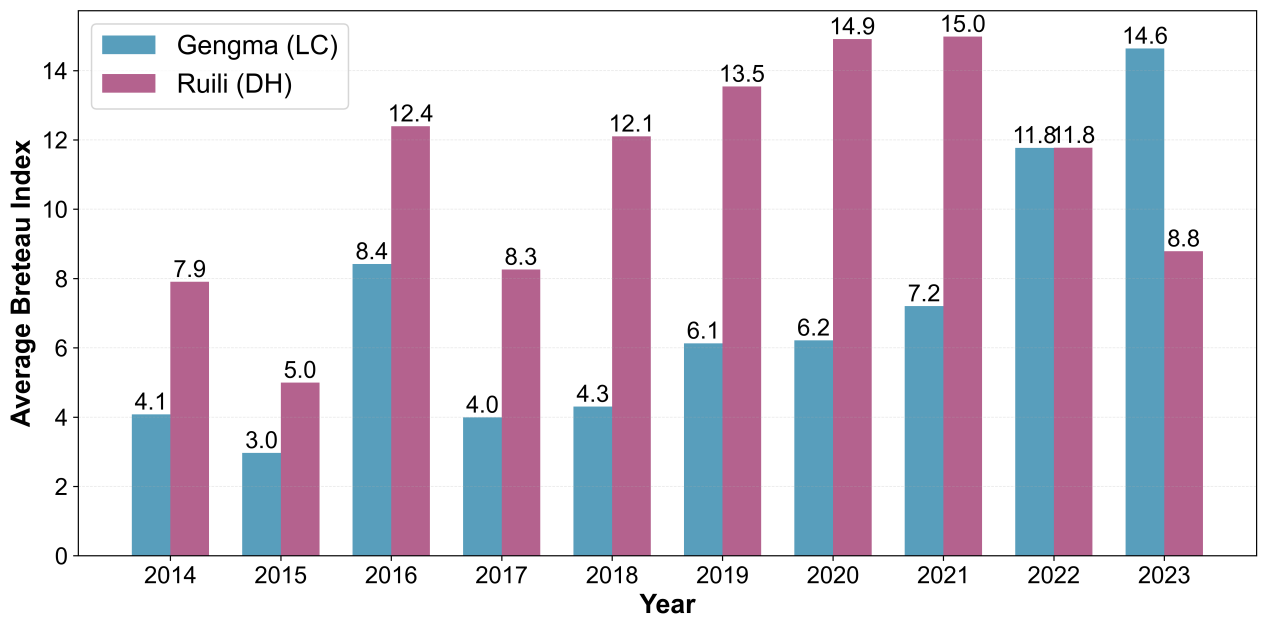
**

**Fig. 1.2 Annual Trend of the Breteau Index, 2014–2023.**

Comparison of monthly trends in the Breteau Index and case numbers between the two locations revealed relatively consistent seasonality (Fig. 1.3 and 1.4).

**
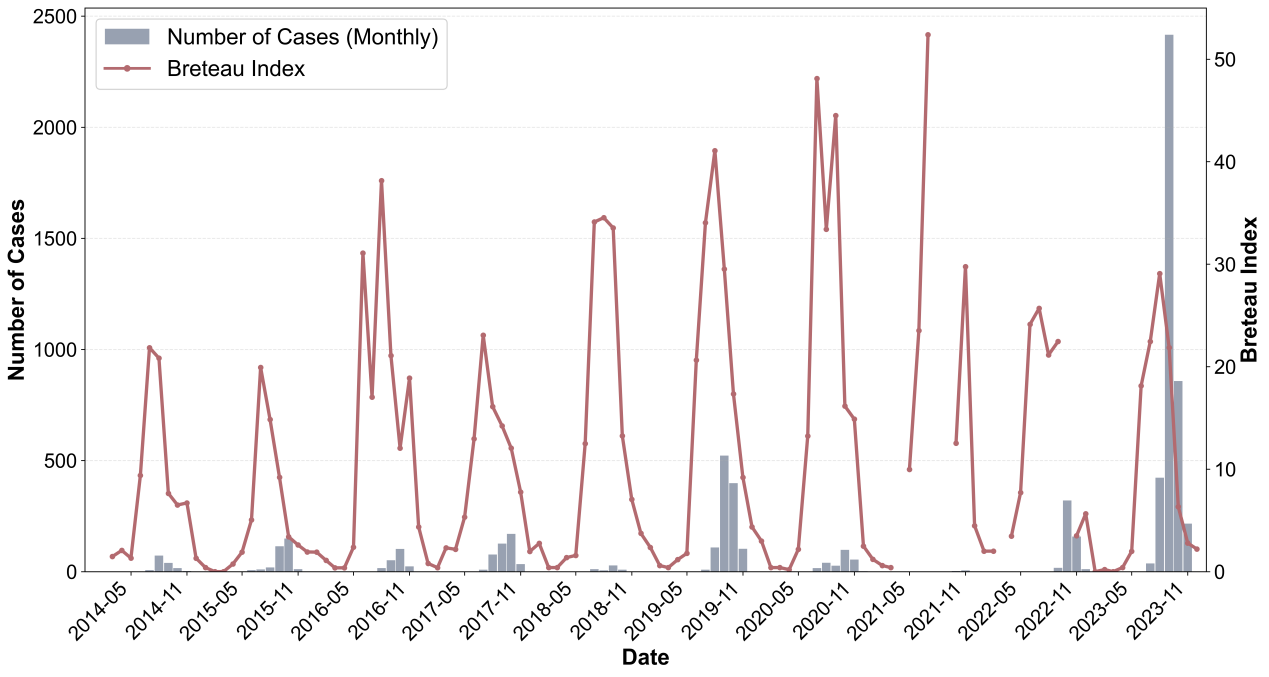
**

**Fig. 1.3 Monthly Trend of the Breteau Index and cases in Dehong Dai and Jingpo Autonomous Prefecture, 2014–2023.**

**
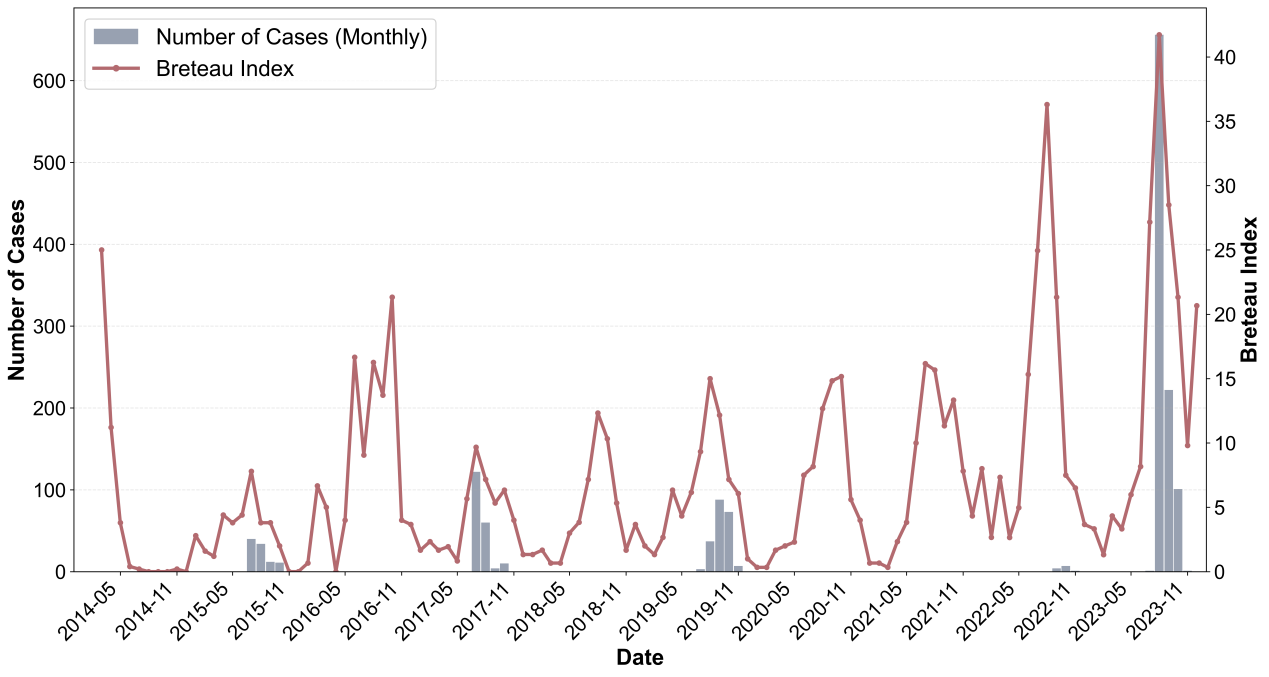
**

**Fig. 1.4 Monthly Trend of the Breteau Index and cases in Lincang City, 2014–2023.**

# Model fitting results


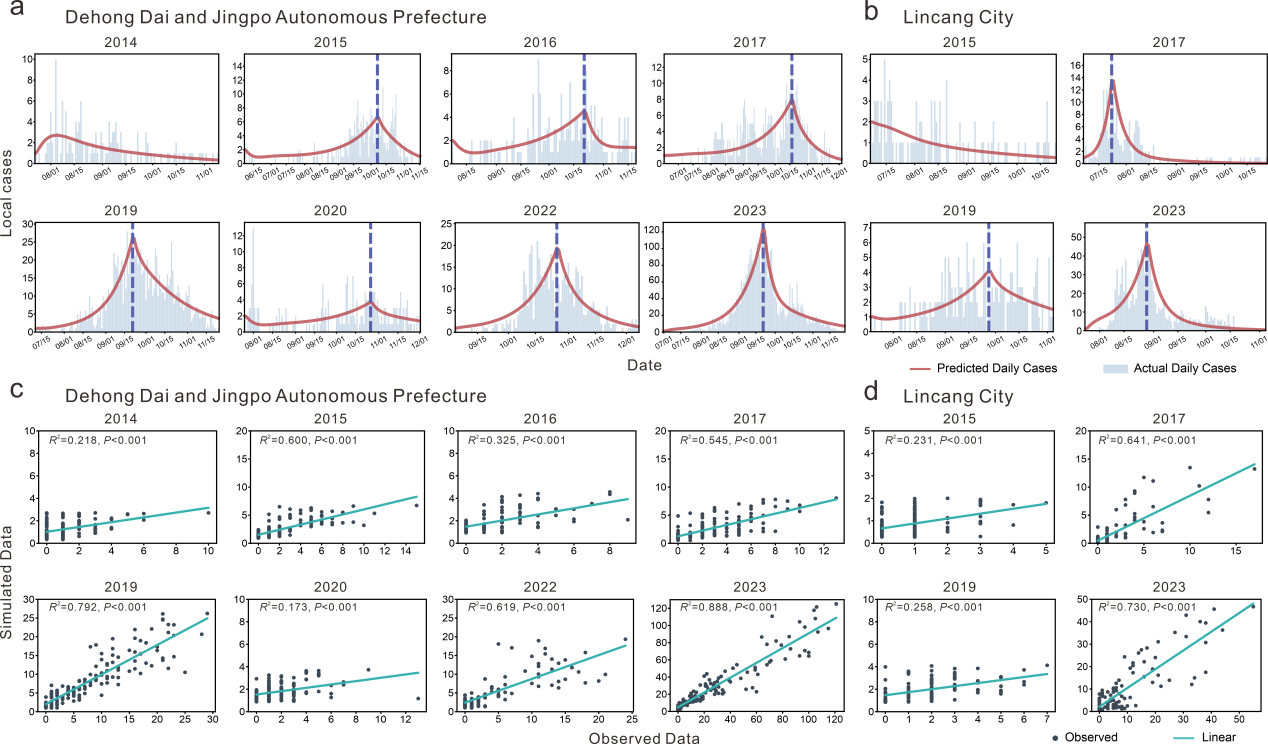


**Fig. 2.1 Model fitting results for Scenario 1.** (A)-(B) Fitting results of dengue fever outbreaks in Dehong Dai and Jingpo Autonomous Prefecture (DH) and Lincang City (LC), respectively, with segmentation points marked by purple dashed lines. (C)-(D) Model fitting performance of dengue fever outbreaks in DH and LC, respectively.


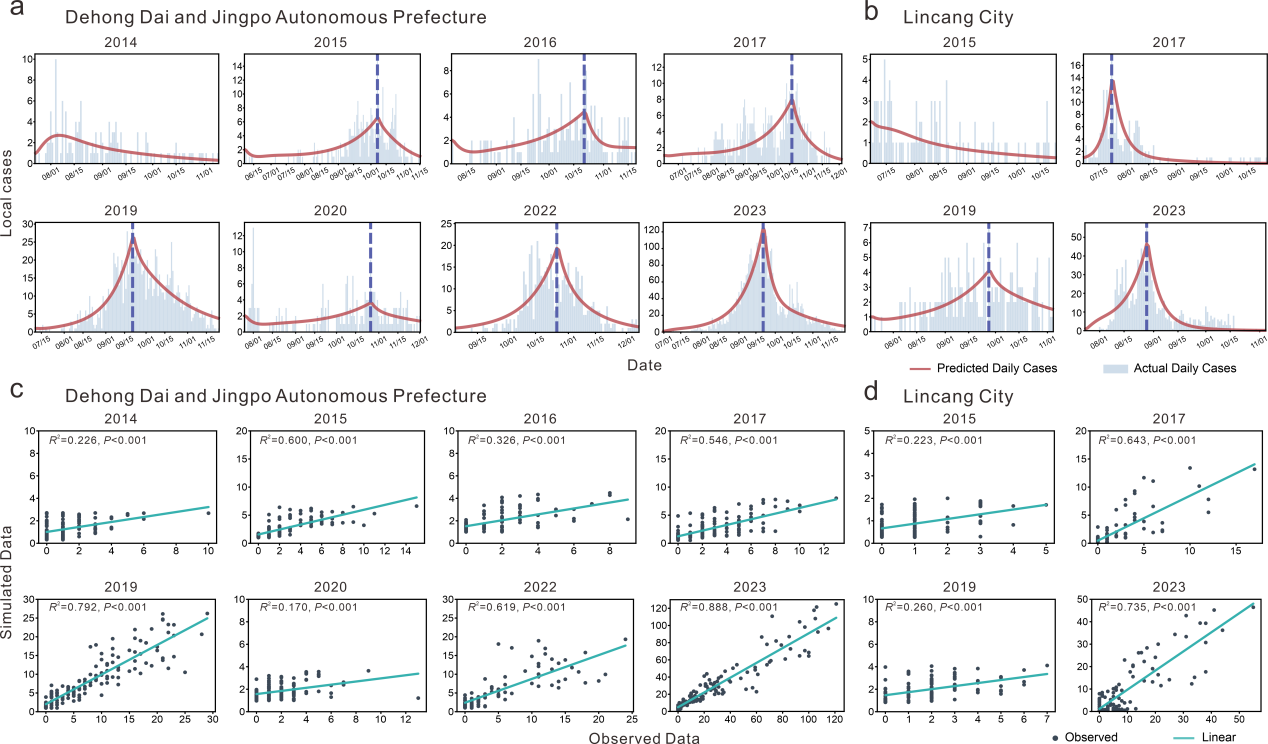


**Fig. 2.2 Model fitting results for Scenario 2.** (A)-(B) Fitting results of dengue fever outbreaks in DH and LC, respectively, with segmentation points marked by purple dashed lines. (C)-(D) Model fitting performance of dengue fever outbreaks in DH and LC, respectively.


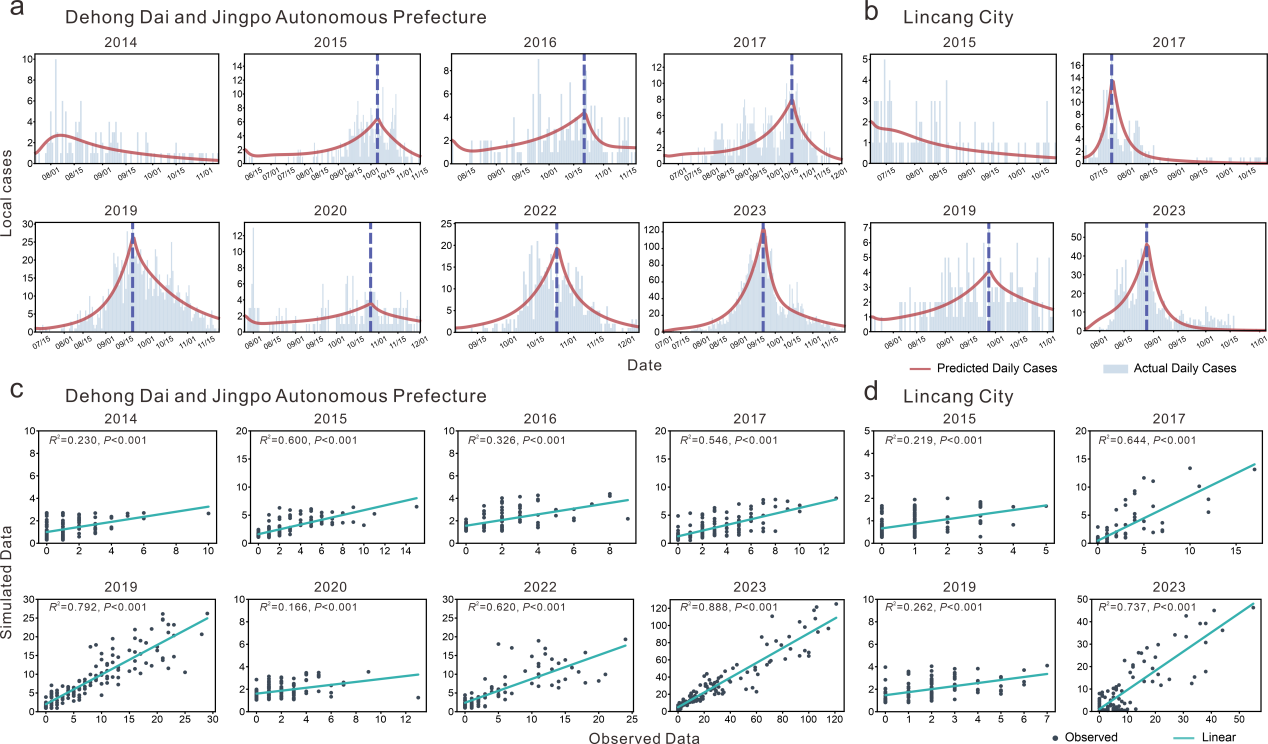


**Fig. 2.3 Model fitting results for Scenario 3.** (A)-(B) Fitting results of dengue fever outbreaks in DH and LC, respectively, with segmentation points marked by purple dashed lines. (C)-(D) Model fitting performance of dengue fever outbreaks in DH and LC, respectively.


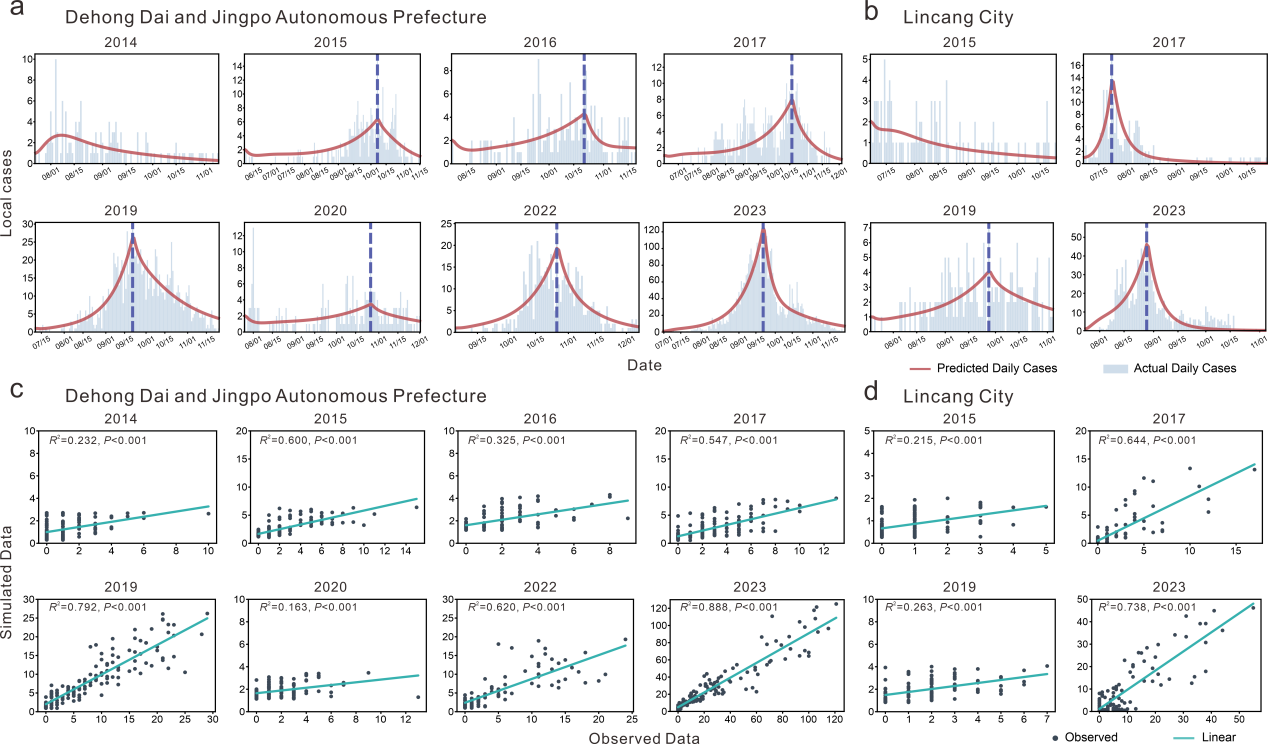


**Fig. 2.4 Model fitting results for Scenario 4.** (A)-(B) Fitting results of dengue fever outbreaks in DH and LC, respectively, with segmentation points marked by purple dashed lines. (C)-(D) Model fitting performance of dengue fever outbreaks in DH and LC, respectively.


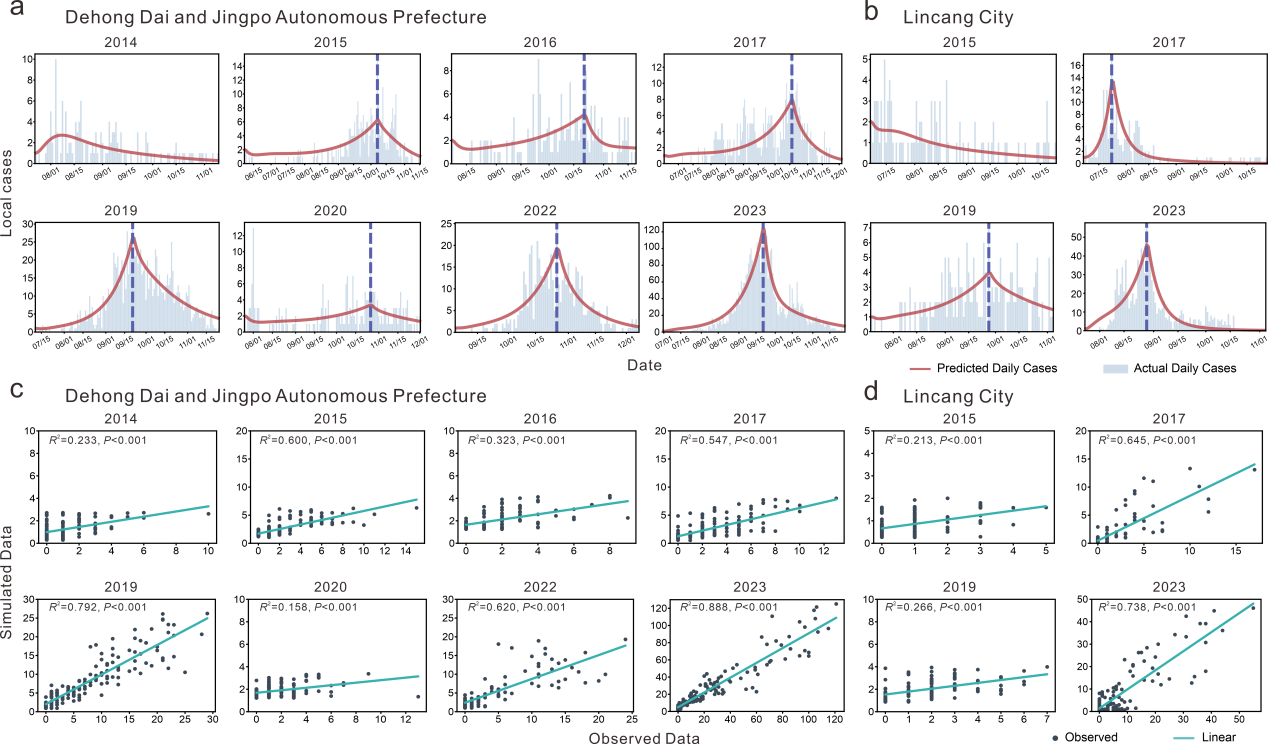


**Fig. 2.5 Model fitting results for Scenario 5.** (A)-(B) Fitting results of dengue fever outbreaks in DH and LC, respectively, with segmentation points marked by purple dashed lines. (C)-(D) Model fitting performance of dengue fever outbreaks in DH and LC, respectively.


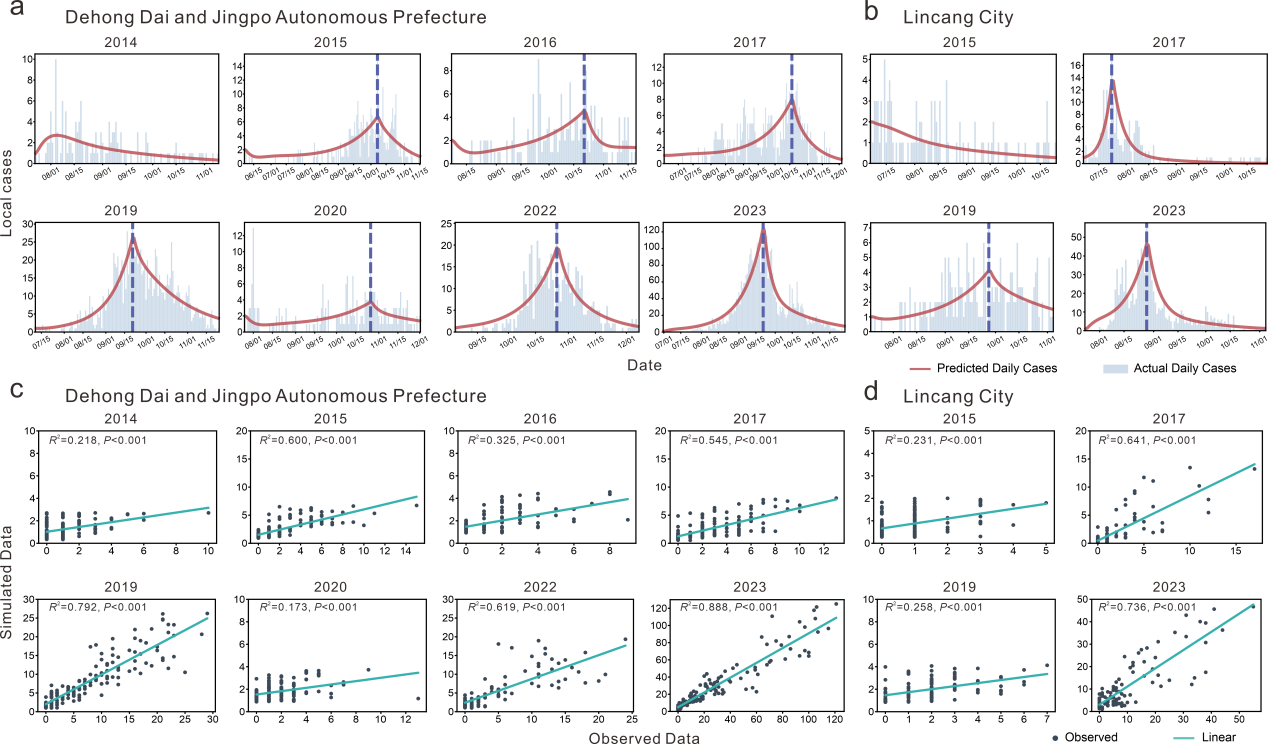


**Fig. 2.6 Model fitting results for Scenario 6.** (A)-(B) Fitting results of dengue fever outbreaks in DH and LC, respectively, with segmentation points marked by purple dashed lines. (C)-(D) Model fitting performance of dengue fever outbreaks in DH and LC, respectively.


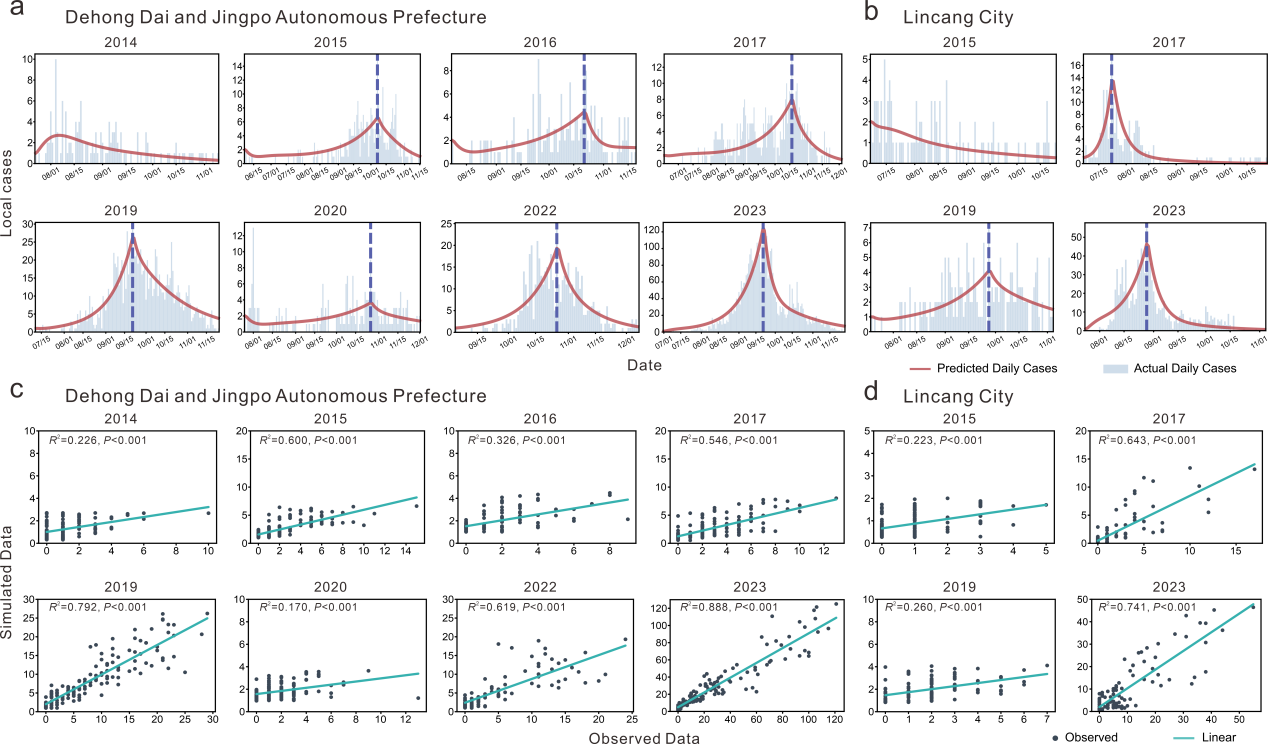


**Fig. 2.7 Model fitting results for Scenario 7.** (A)-(B) Fitting results of dengue fever outbreaks in DH and LC, respectively, with segmentation points marked by purple dashed lines. (C)-(D) Model fitting performance of dengue fever outbreaks in DH and LC, respectively.


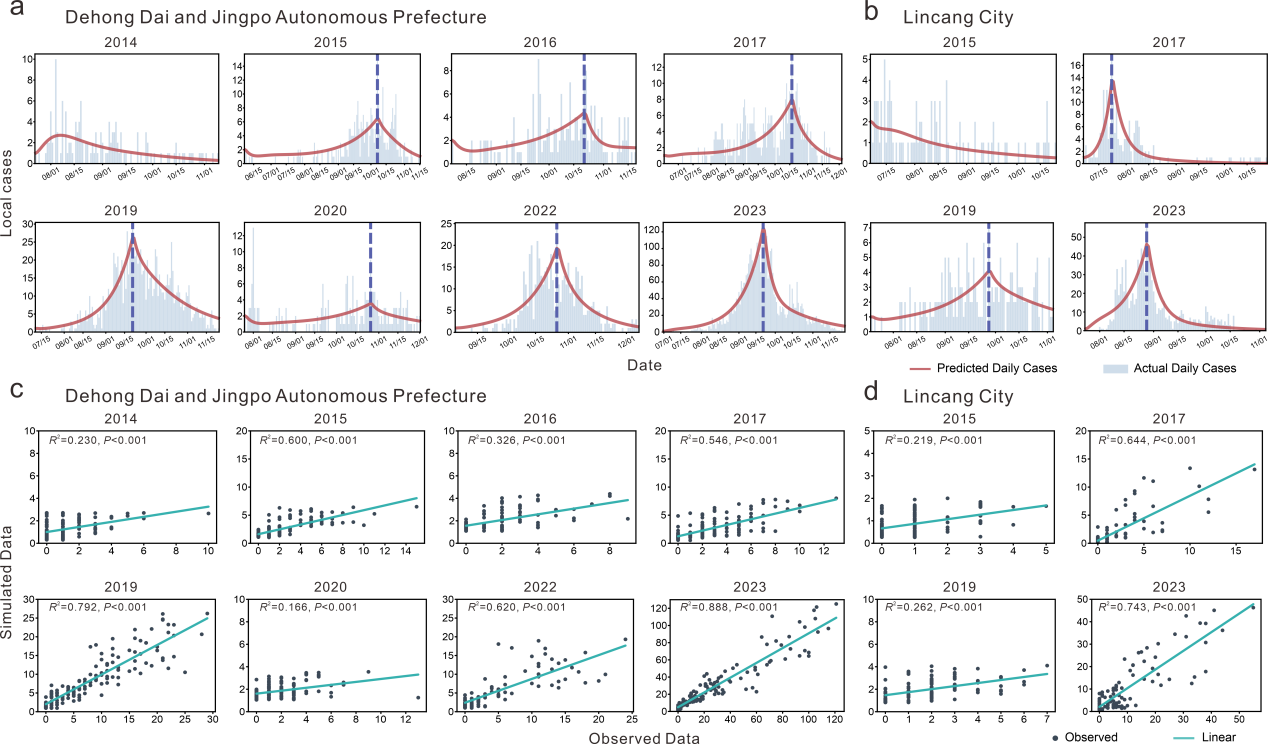


**Fig. 2.8 Model fitting results for Scenario 8.** (A)-(B) Fitting results of dengue fever outbreaks in DH and LC, respectively, with segmentation points marked by purple dashed lines. (C)-(D) Model fitting performance of dengue fever outbreaks in DH and LC, respectively.


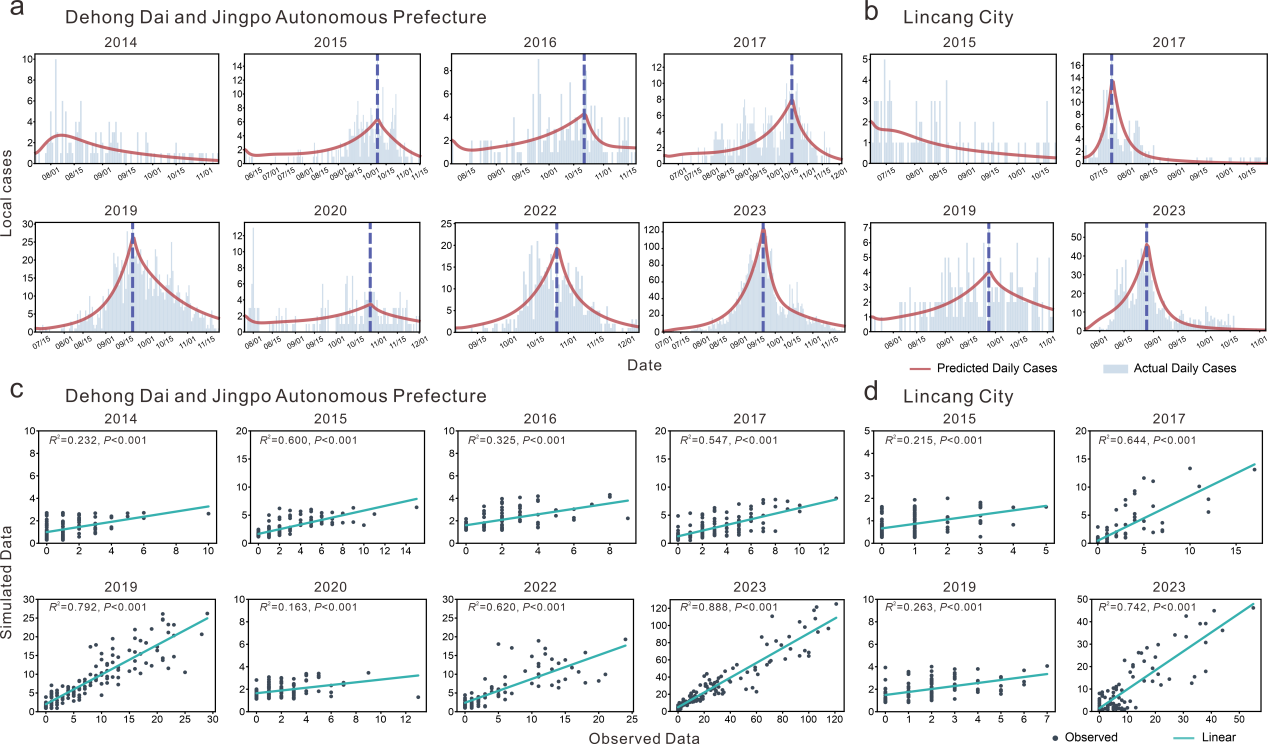


**Fig. 2.9 Model fitting results for Scenario 9.** (A)-(B) Fitting results of dengue fever outbreaks in DH and LC, respectively, with segmentation points marked by purple dashed lines. (C)-(D) Model fitting performance of dengue fever outbreaks in DH and LC, respectively.


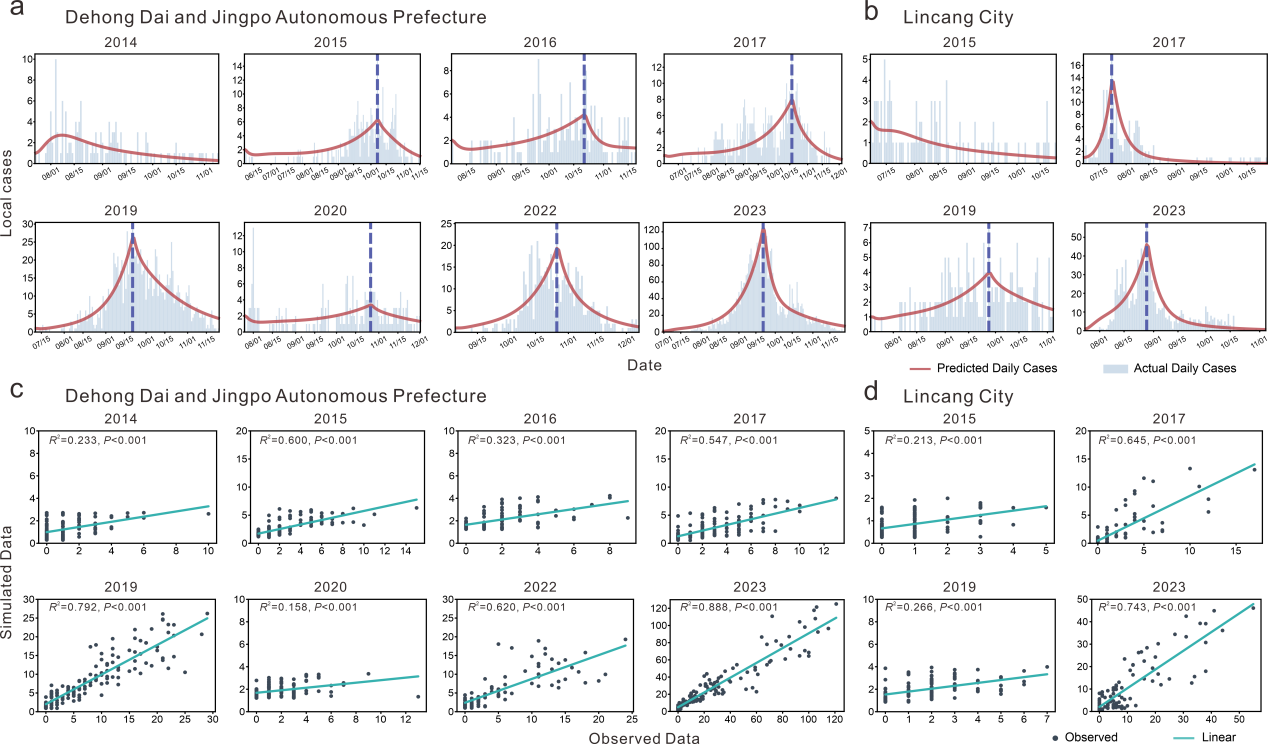


**Fig. 2.10 Model fitting results for Scenario 10.** (A)-(B) Fitting results of dengue fever outbreaks in DH and LC, respectively, with segmentation points marked by purple dashed lines. (C)-(D) Model fitting performance of dengue fever outbreaks in DH and LC, respectively.


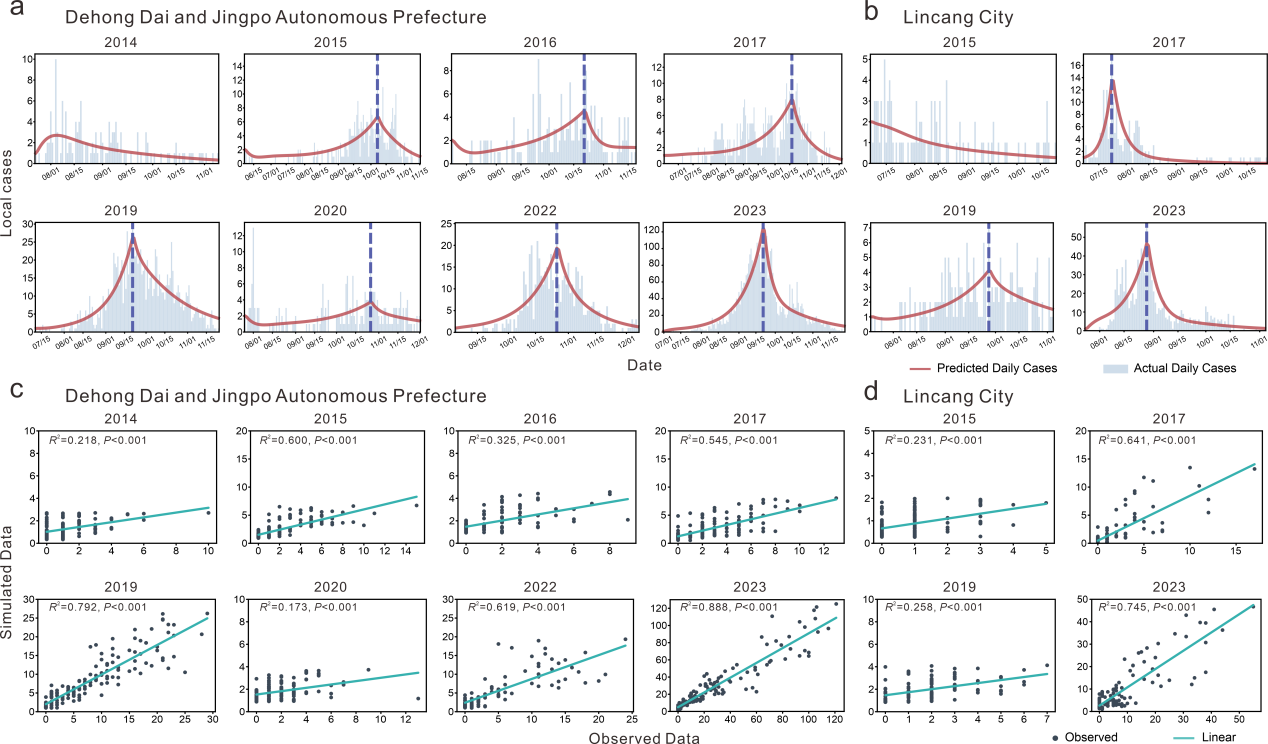


**Fig. 2.11 Model fitting results for Scenario 11.** (A)-(B) Fitting results of dengue fever outbreaks in DH and LC, respectively, with segmentation points marked by purple dashed lines. (C)-(D) Model fitting performance of dengue fever outbreaks in DH and LC, respectively.


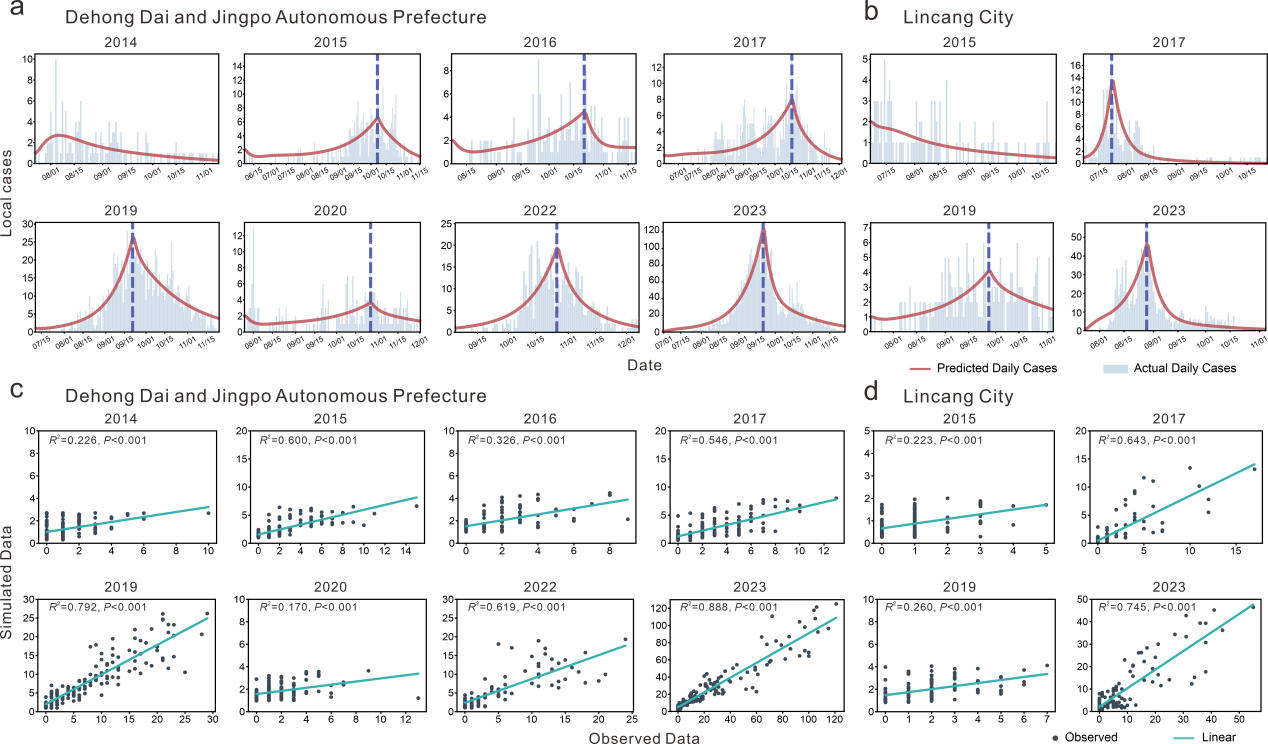


**Fig. 2.12 Model fitting results for Scenario 12.** (A)-(B) Fitting results of dengue fever outbreaks in DH and LC, respectively, with segmentation points marked by purple dashed lines. (C)-(D) Model fitting performance of dengue fever outbreaks in DH and LC, respectively.


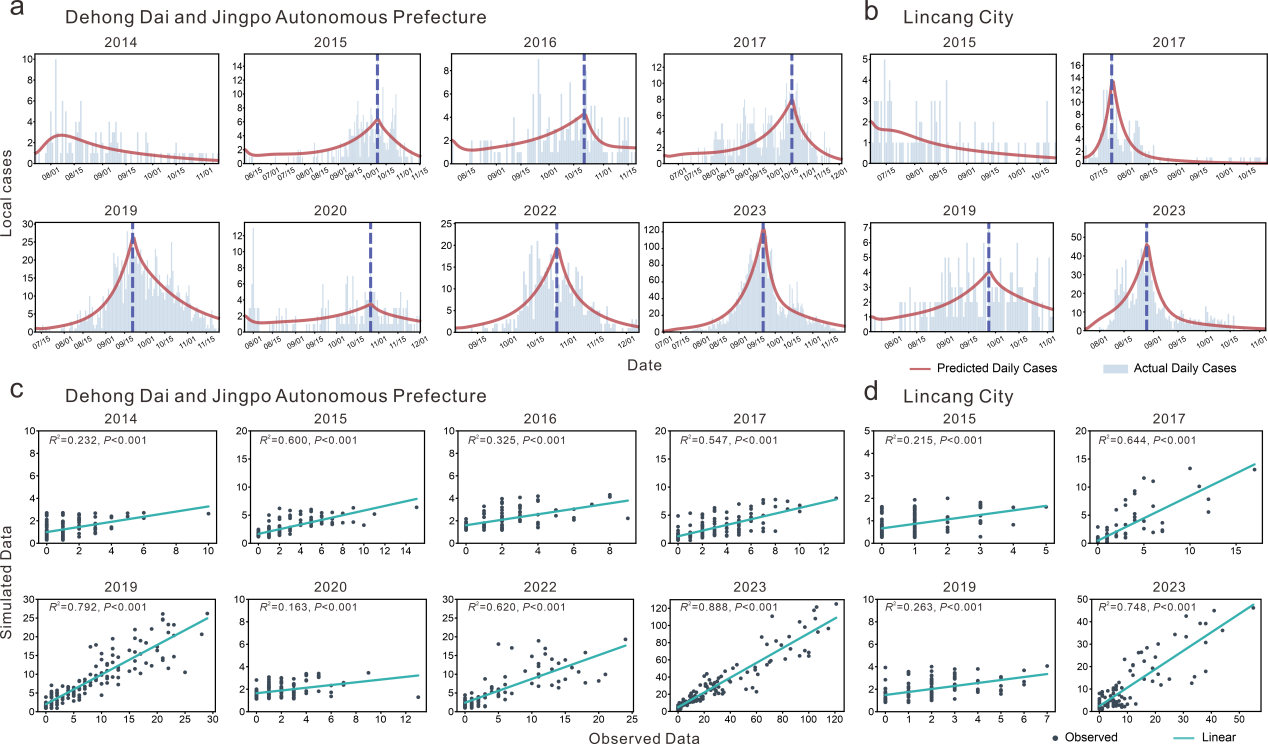


**Fig. 2.13 Model fitting results for Scenario 14.** (A)-(B) Fitting results of dengue fever outbreaks in DH and LC, respectively, with segmentation points marked by purple dashed lines. (C)-(D) Model fitting performance of dengue fever outbreaks in DH and LC, respectively.


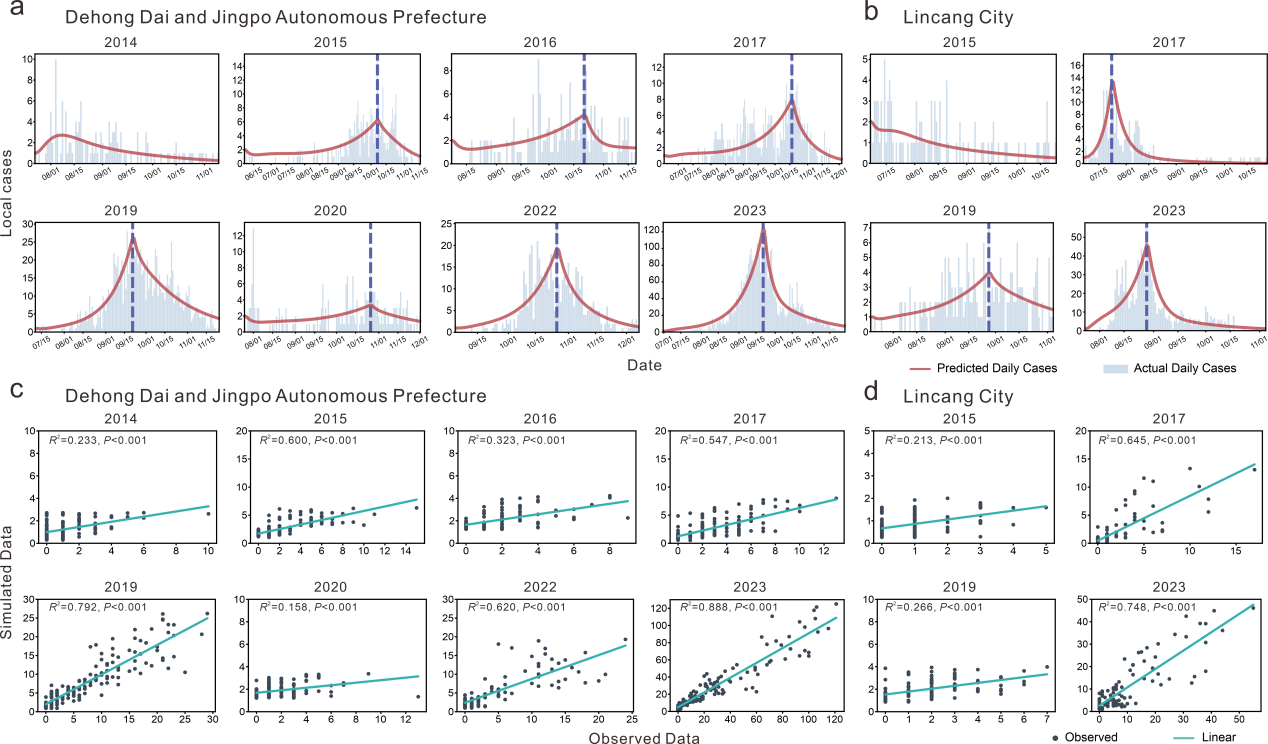


**Fig. 2.14 Model fitting results for Scenario 15.** (A)-(B) Fitting results of dengue fever outbreaks in DH and LC, respectively, with segmentation points marked by purple dashed lines. (C)-(D) Model fitting performance of dengue fever outbreaks in DH and LC, respectively.


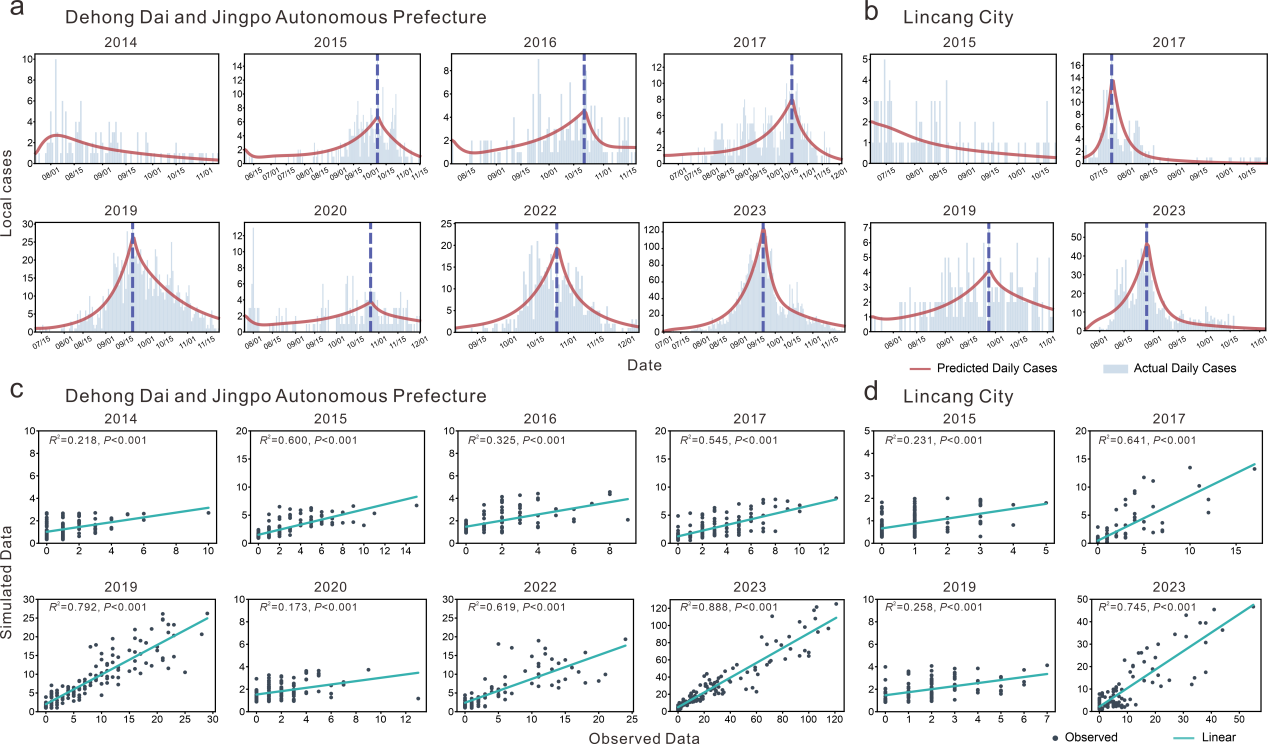


**Fig. 2.15 Model fitting results for Scenario 16.** (A)-(B) Fitting results of dengue fever outbreaks in DH and LC, respectively, with segmentation points marked by purple dashed lines. (C)-(D) Model fitting performance of dengue fever outbreaks in DH and LC, respectively.


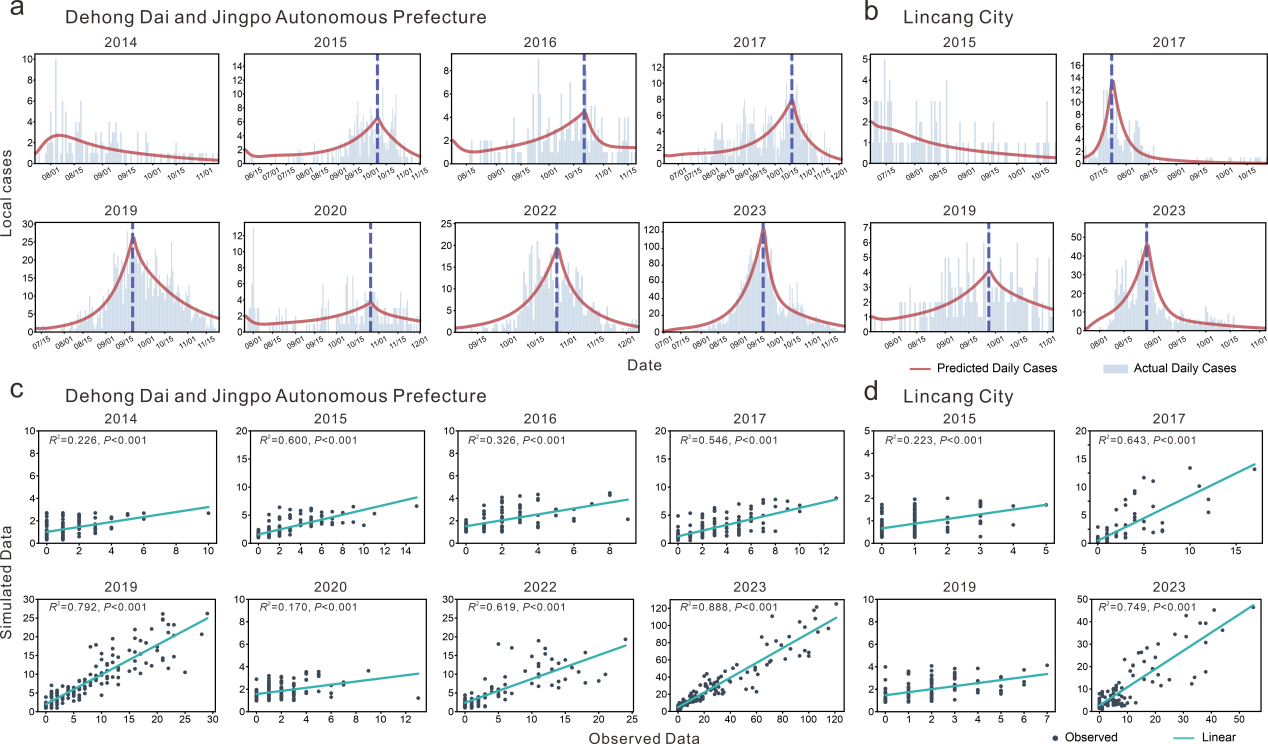


**Fig. 2.16 Model fitting results for Scenario 17.** (A)-(B) Fitting results of dengue fever outbreaks in DH and LC, respectively, with segmentation points marked by purple dashed lines. (C)-(D) Model fitting performance of dengue fever outbreaks in DH and LC, respectively.


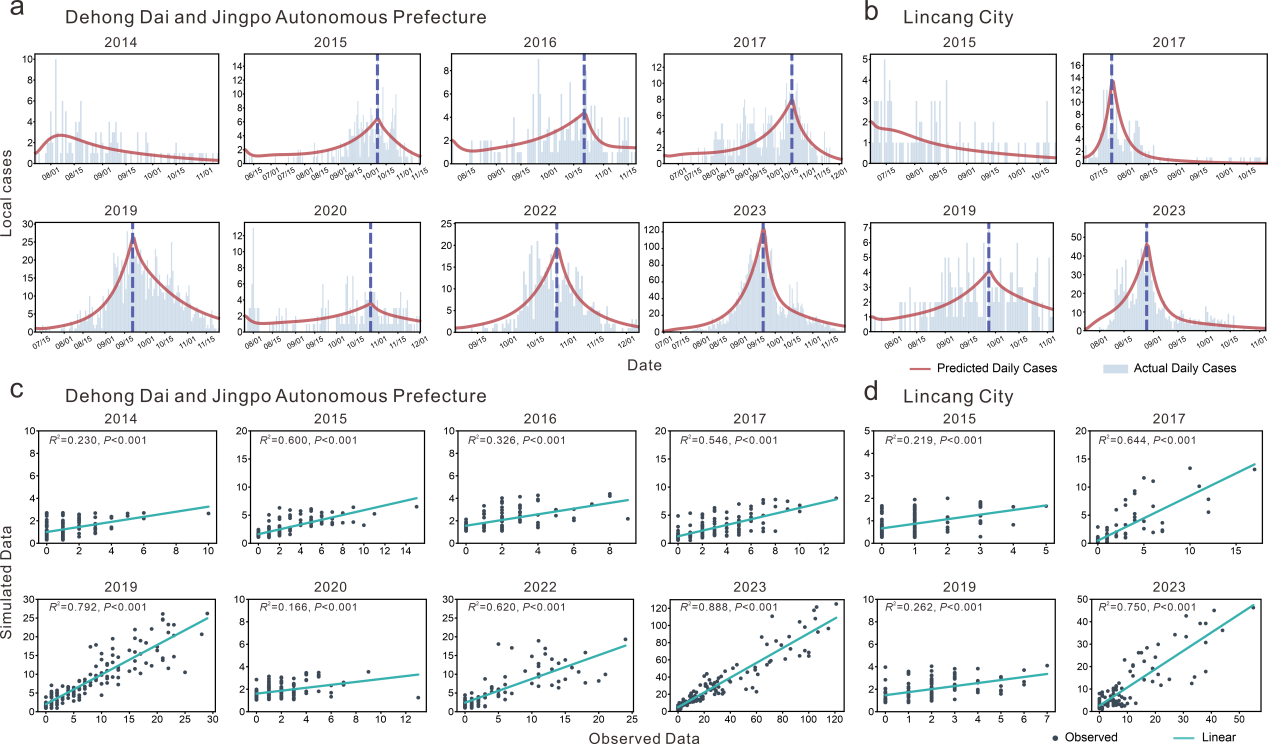


**Fig. 2.17 Model fitting results for Scenario 18.** (A)-(B) Fitting results of dengue fever outbreaks in DH and LC, respectively, with segmentation points marked by purple dashed lines. (C)-(D) Model fitting performance of dengue fever outbreaks in DH and LC, respectively.


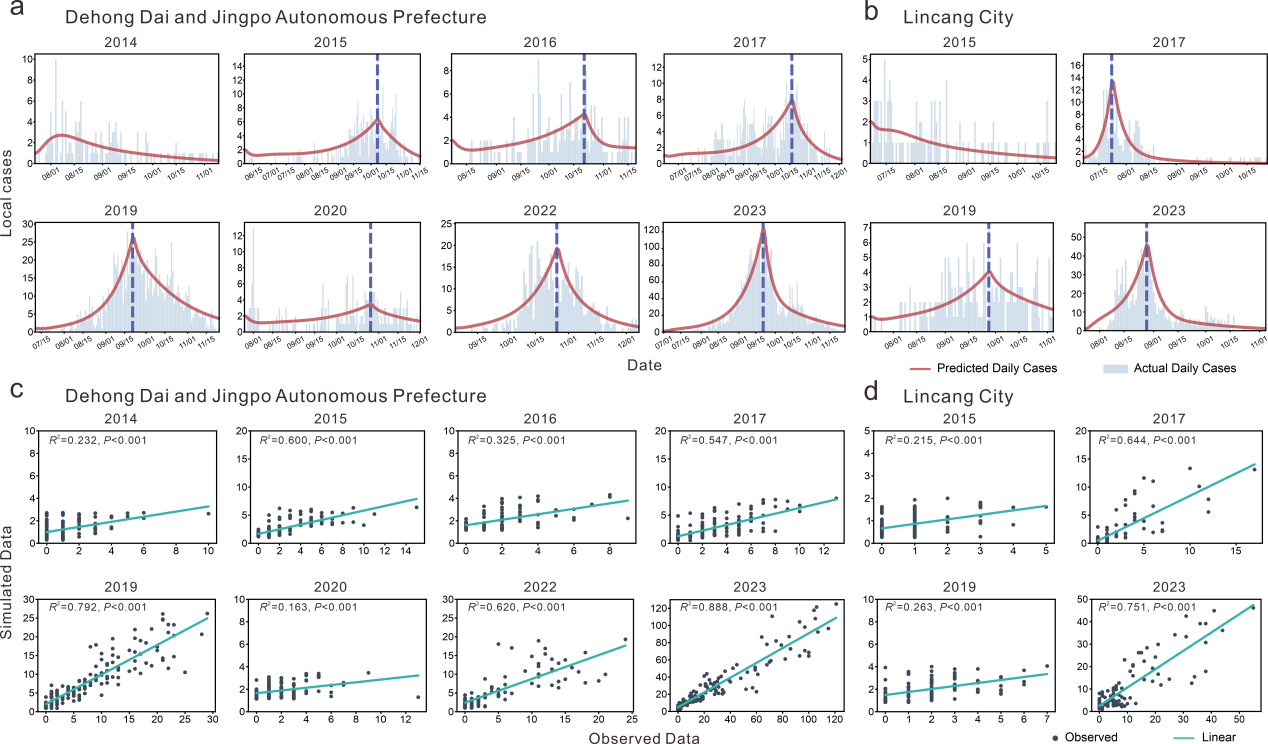


**Fig. 2.18 Model fitting results for Scenario 19.** (A)-(B) Fitting results of dengue fever outbreaks in DH and LC, respectively, with segmentation points marked by purple dashed lines. (C)-(D) Model fitting performance of dengue fever outbreaks in DH and LC, respectively.


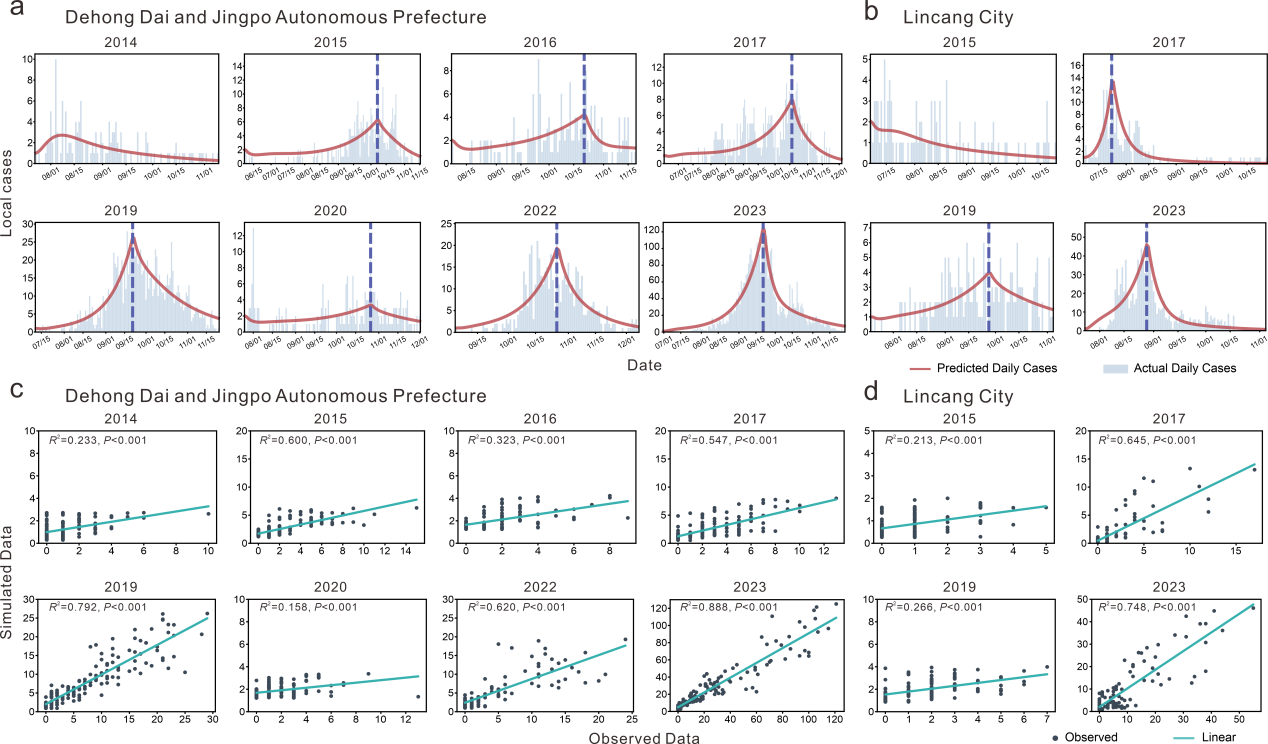


**Fig. 2.19 Model fitting results for Scenario 20.** (A)-(B) Fitting results of dengue fever outbreaks in DH and LC, respectively, with segmentation points marked by purple dashed lines. (C)-(D) Model fitting performance of dengue fever outbreaks in DH and LC, respectively.


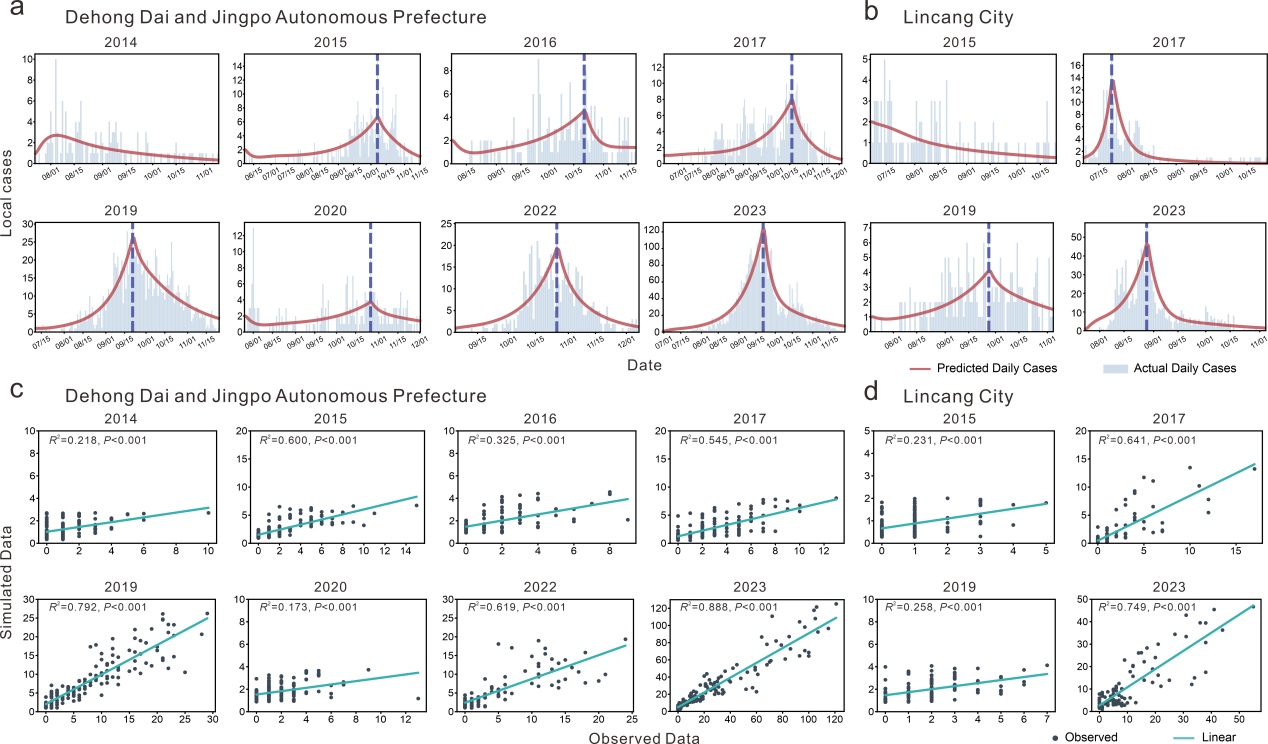


**Fig. 2.20 Model fitting results for Scenario 21.** (A)-(B) Fitting results of dengue fever outbreaks in DH and LC, respectively, with segmentation points marked by purple dashed lines. (C)-(D) Model fitting performance of dengue fever outbreaks in DH and LC, respectively.


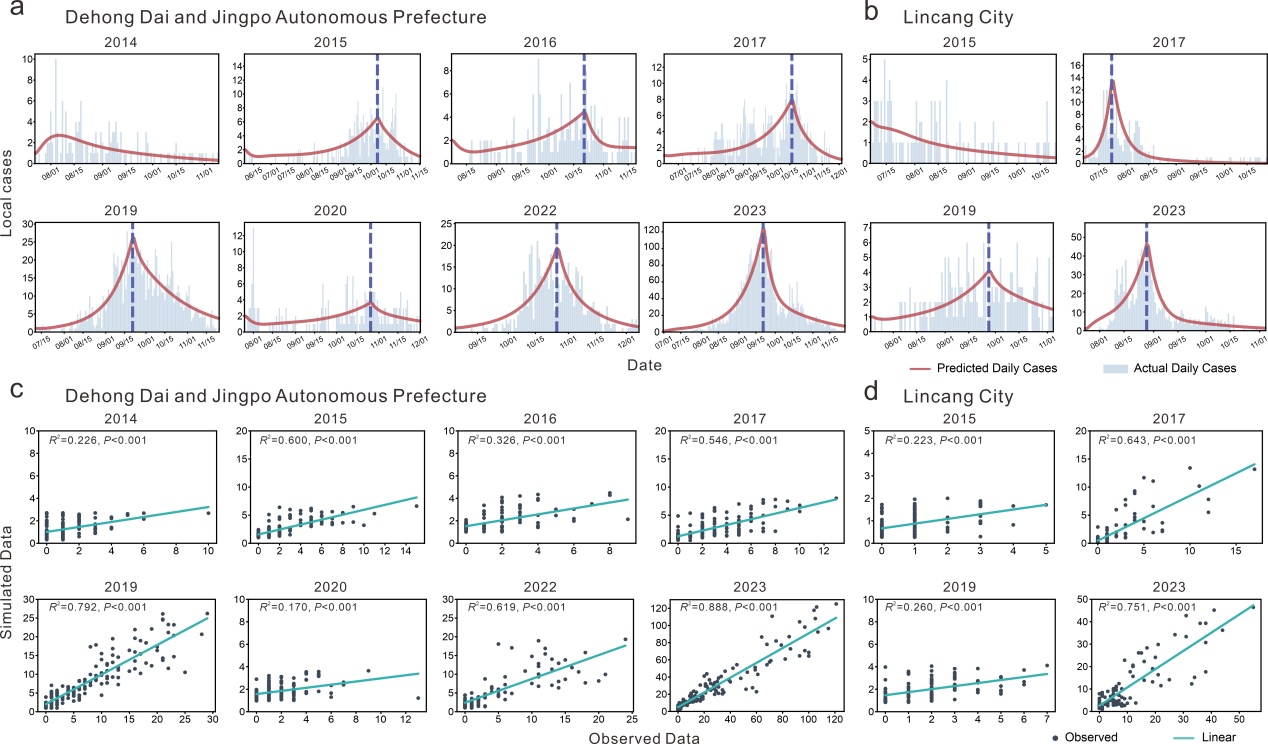


**Fig. 2.21 Model fitting results for Scenario 22.** (A)-(B) Fitting results of dengue fever outbreaks in DH and LC, respectively, with segmentation points marked by purple dashed lines. (C)-(D) Model fitting performance of dengue fever outbreaks in DH and LC, respectively.


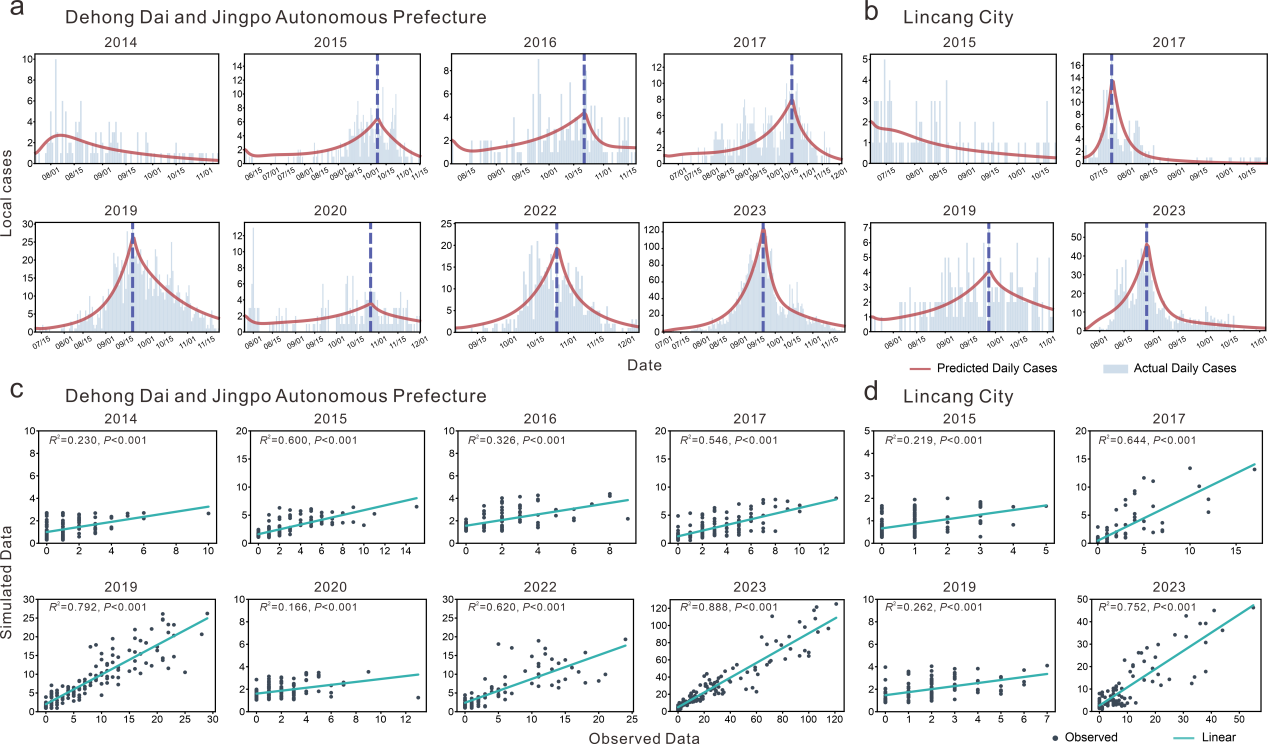


**Fig. 2.22 Model fitting results for Scenario 23.** (A)-(B) Fitting results of dengue fever outbreaks in DH and LC, respectively, with segmentation points marked by purple dashed lines. (C)-(D) Model fitting performance of dengue fever outbreaks in DH and LC, respectively.


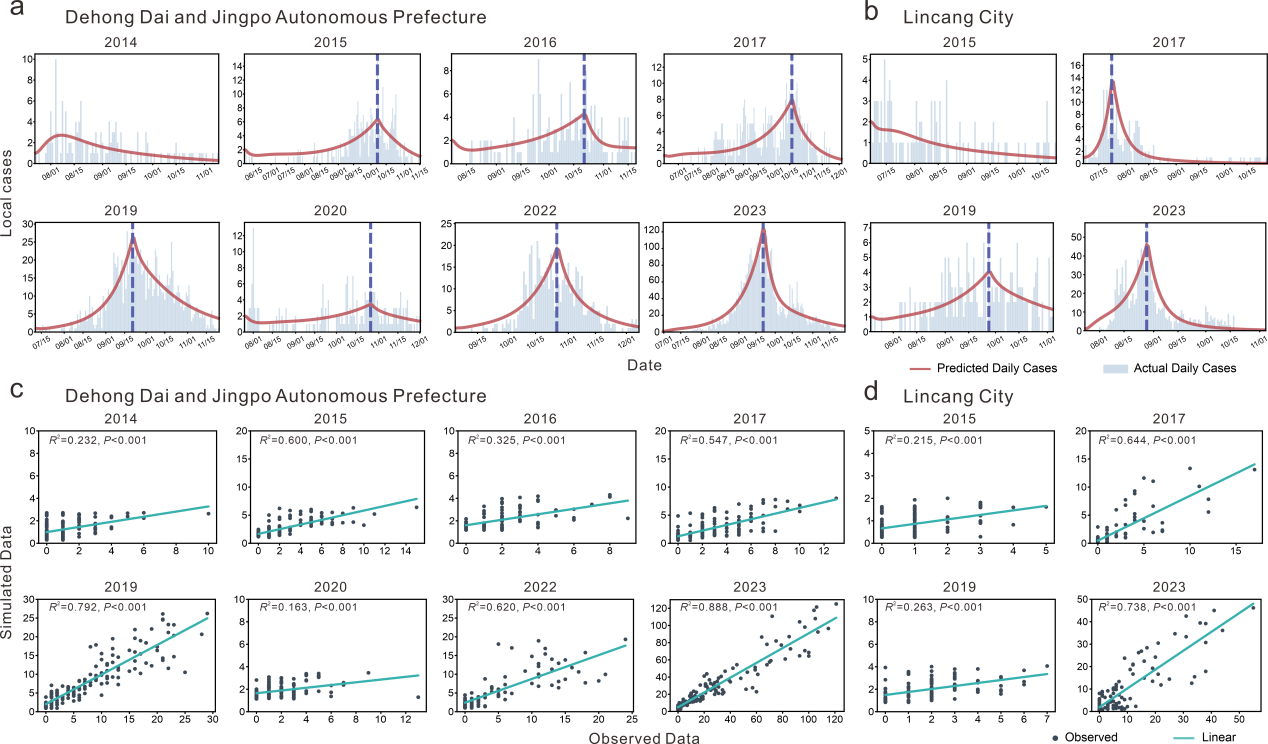


**Fig. 2.23 Model fitting results for Scenario 24.** (A)-(B) Fitting results of dengue fever outbreaks in DH and LC, respectively, with segmentation points marked by purple dashed lines. (C)-(D) Model fitting performance of dengue fever outbreaks in DH and LC, respectively.


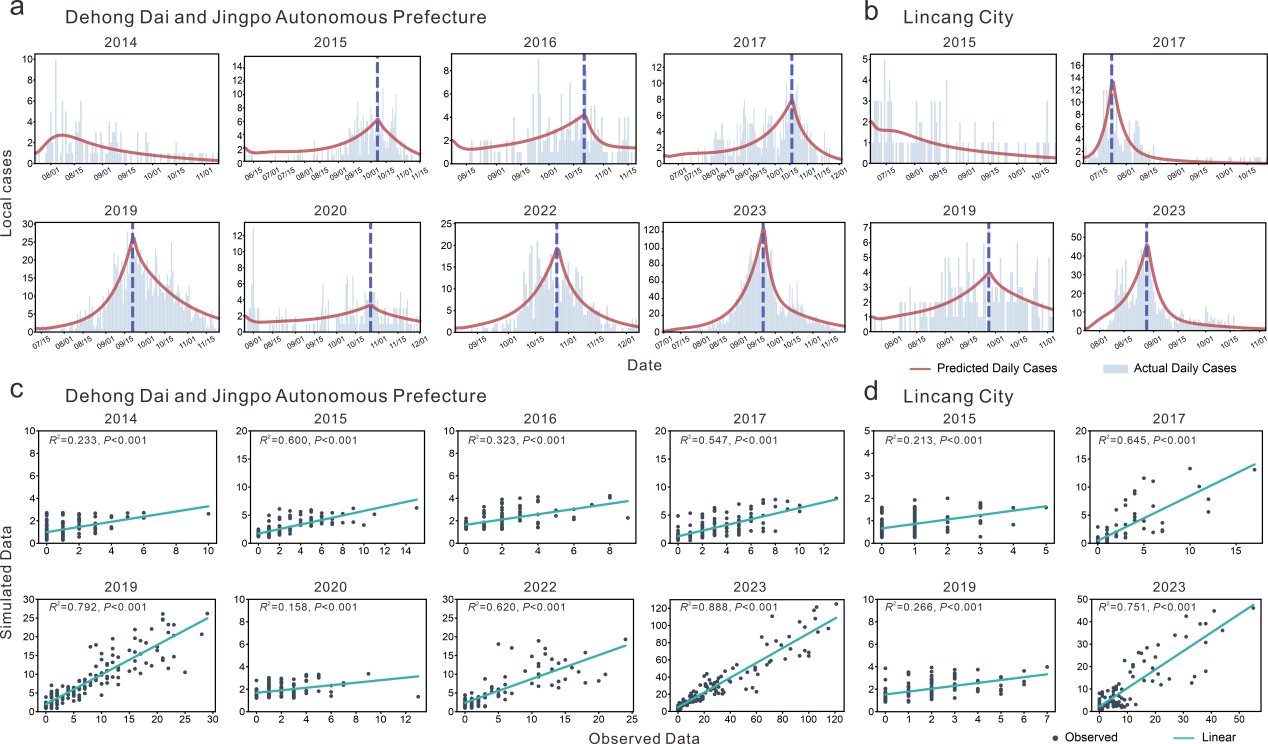


**Fig. 2.24 Model fitting results for Scenario 25.** (A)-(B) Fitting results of dengue fever outbreaks in DH and LC, respectively, with segmentation points marked by purple dashed lines. (C)-(D) Model fitting performance of dengue fever outbreaks in DH and LC, respectively.

# Dengue fever transmissibility *R_t_* for all scenarios


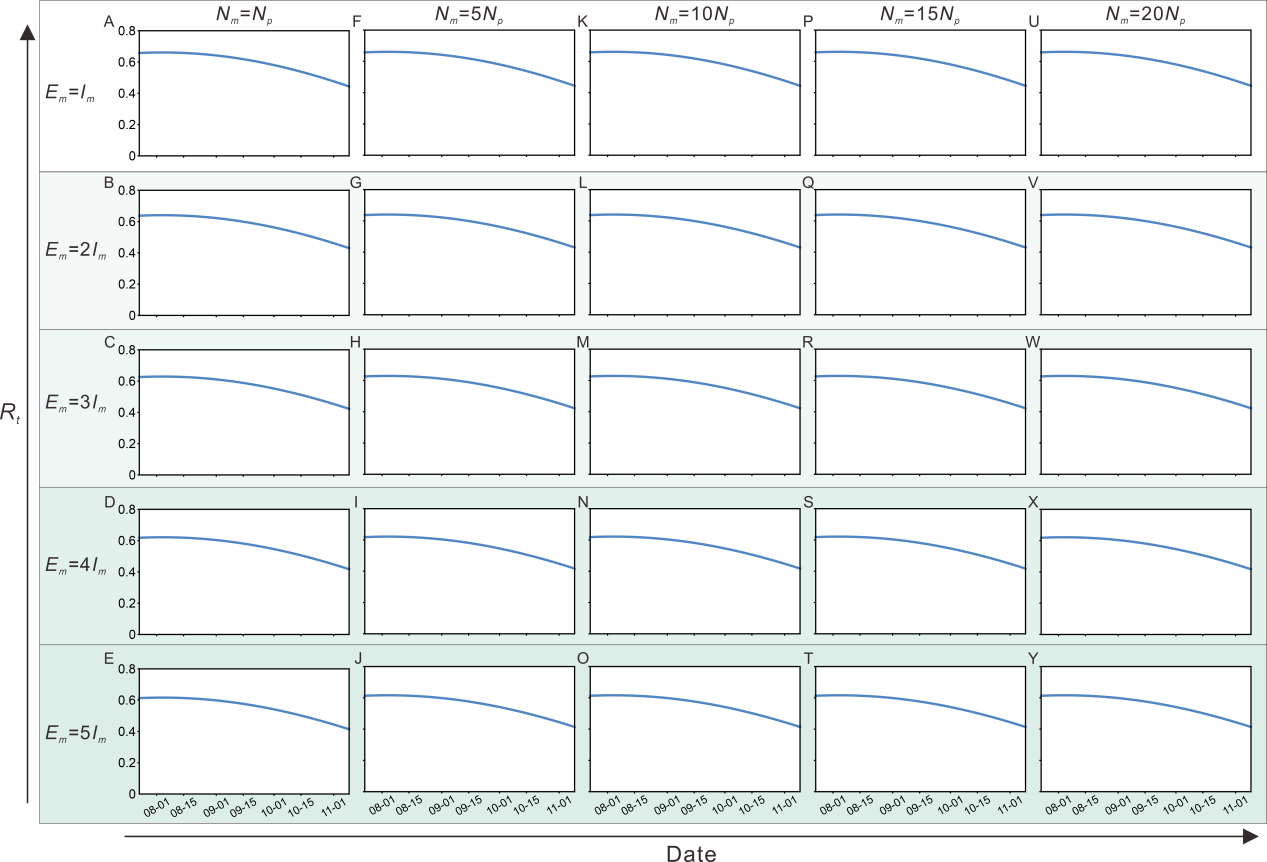


**Fig. 3.1 Calculation results of the dengue fever transmissibility in Dehong Dai and Jingpo Autonomous Prefecture, 2014.** (A)-(Y) for scenario 1-25.


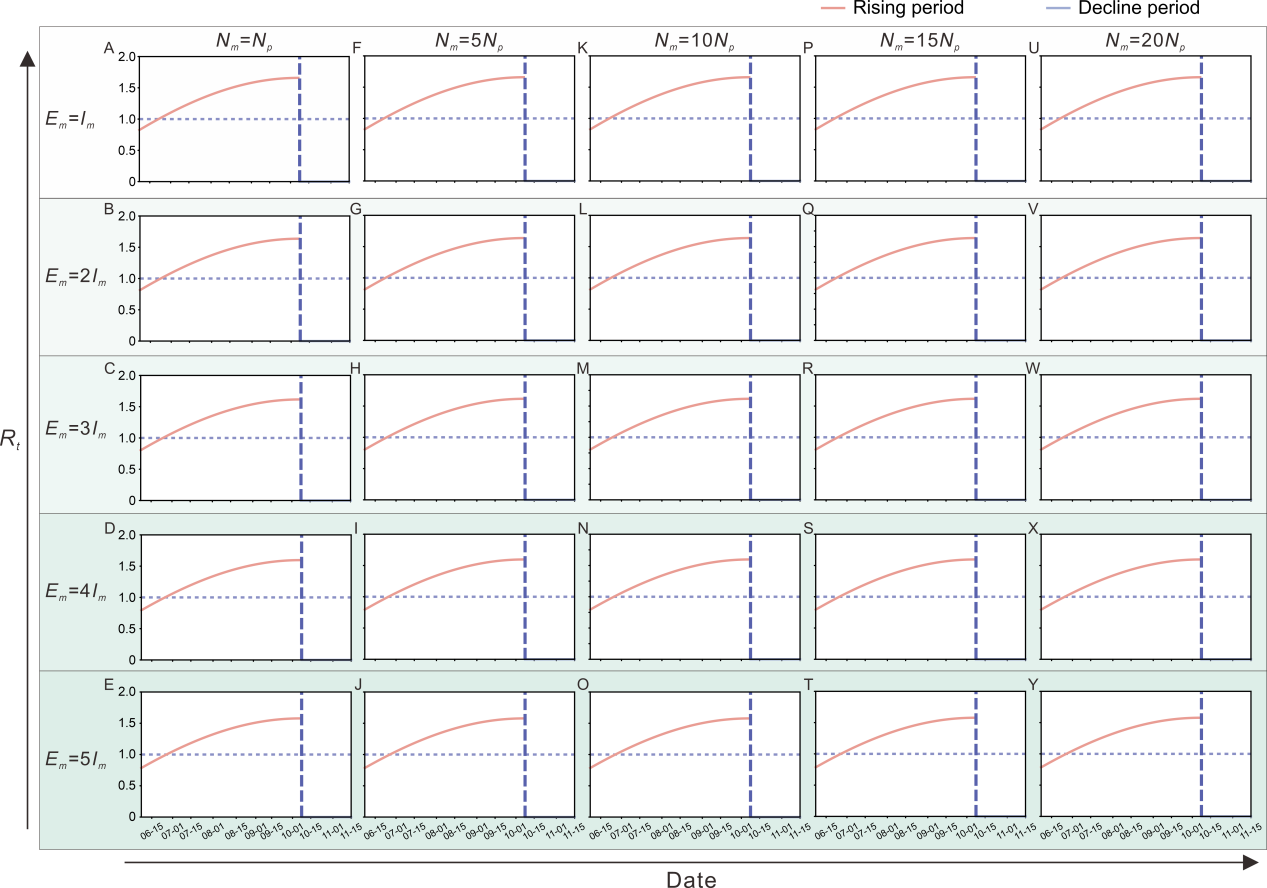


**Fig. 3.2 Calculation results of the dengue fever transmissibility in Dehong Dai and Jingpo Autonomous Prefecture, 2015.** (A)-(Y) for scenario 1-25, with segmentation points marked by purple dashed lines.


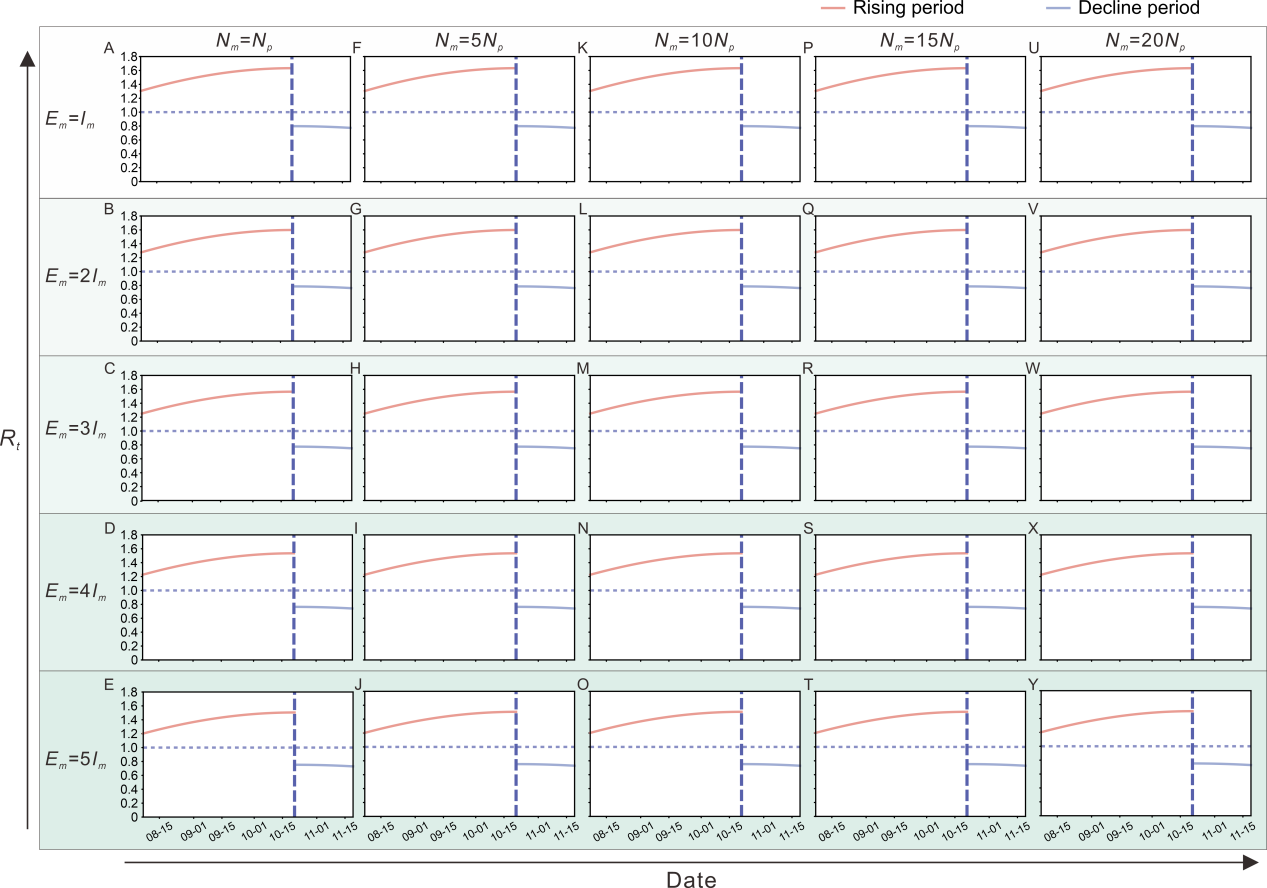


**Fig. 3.3 Calculation results of the dengue fever transmissibility in Dehong Dai and Jingpo Autonomous Prefecture, 2016.** (A)-(Y) for scenario 1-25, with segmentation points marked by purple dashed lines.


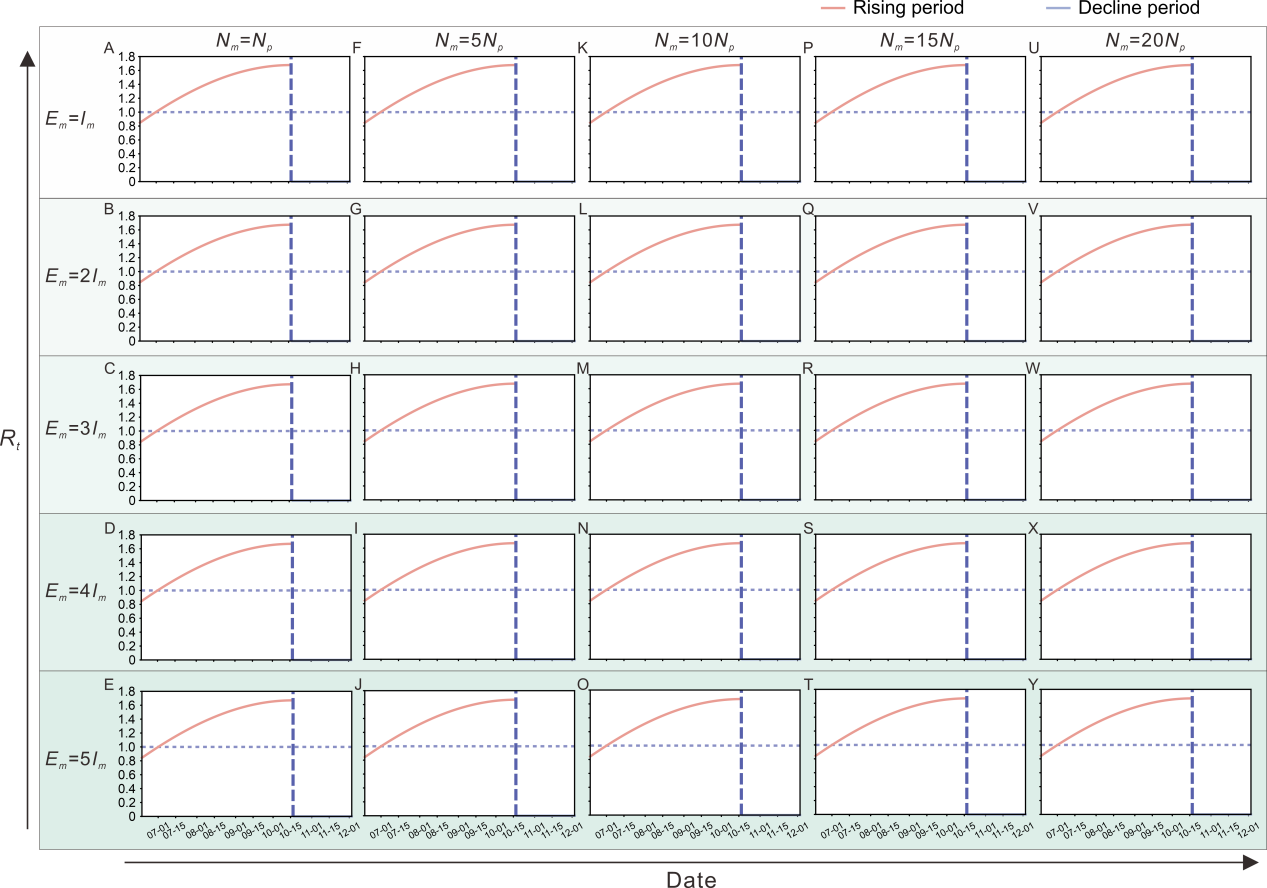


**Fig. 3.4 Calculation results of the dengue fever transmissibility in Dehong Dai and Jingpo Autonomous Prefecture, 2017.** (A)-(Y) for scenario 1-25, with segmentation points marked by purple dashed lines.


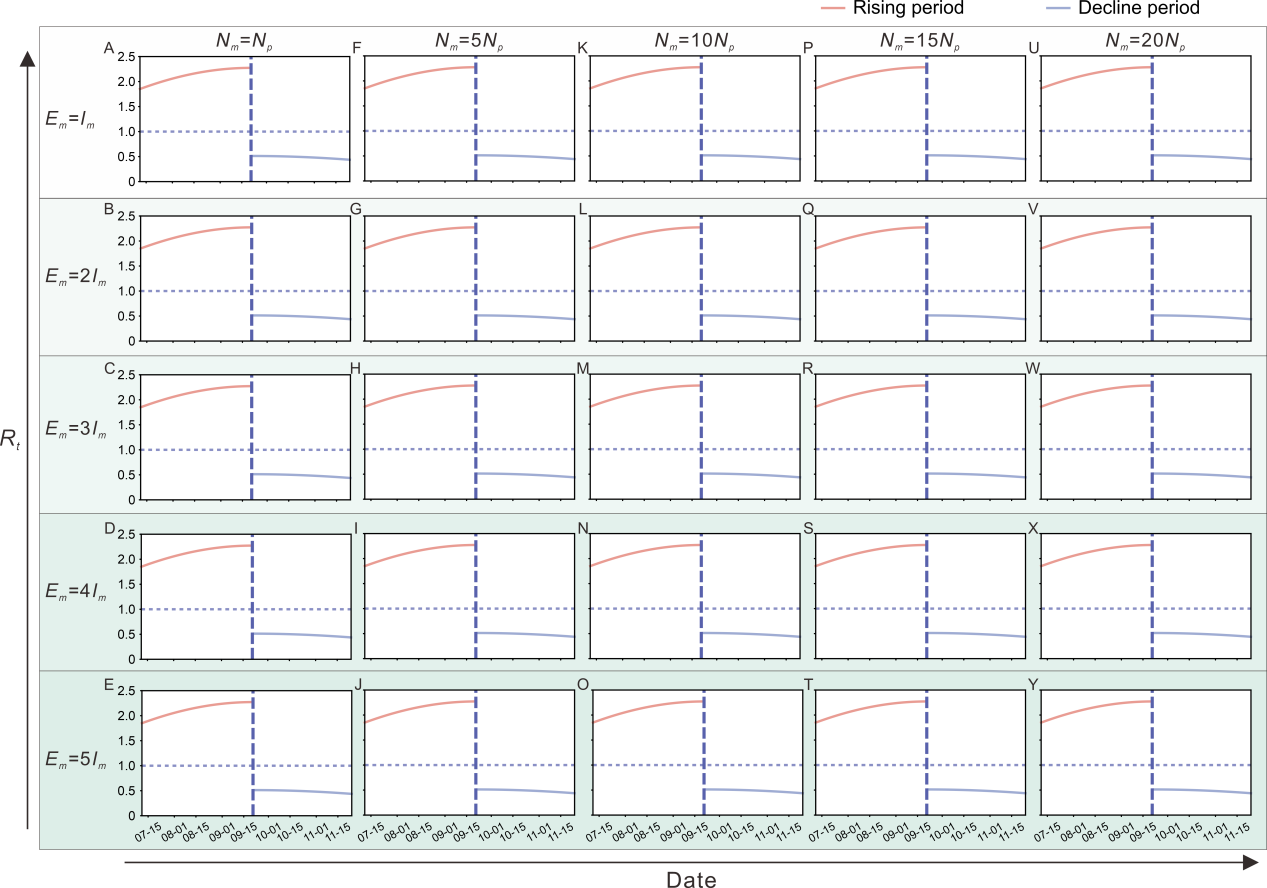


**Fig. 3.5 Calculation results of the dengue fever transmissibility in Dehong Dai and Jingpo Autonomous Prefecture, 2019.** (A)-(Y) for scenario 1-25, with segmentation points marked by purple dashed lines.


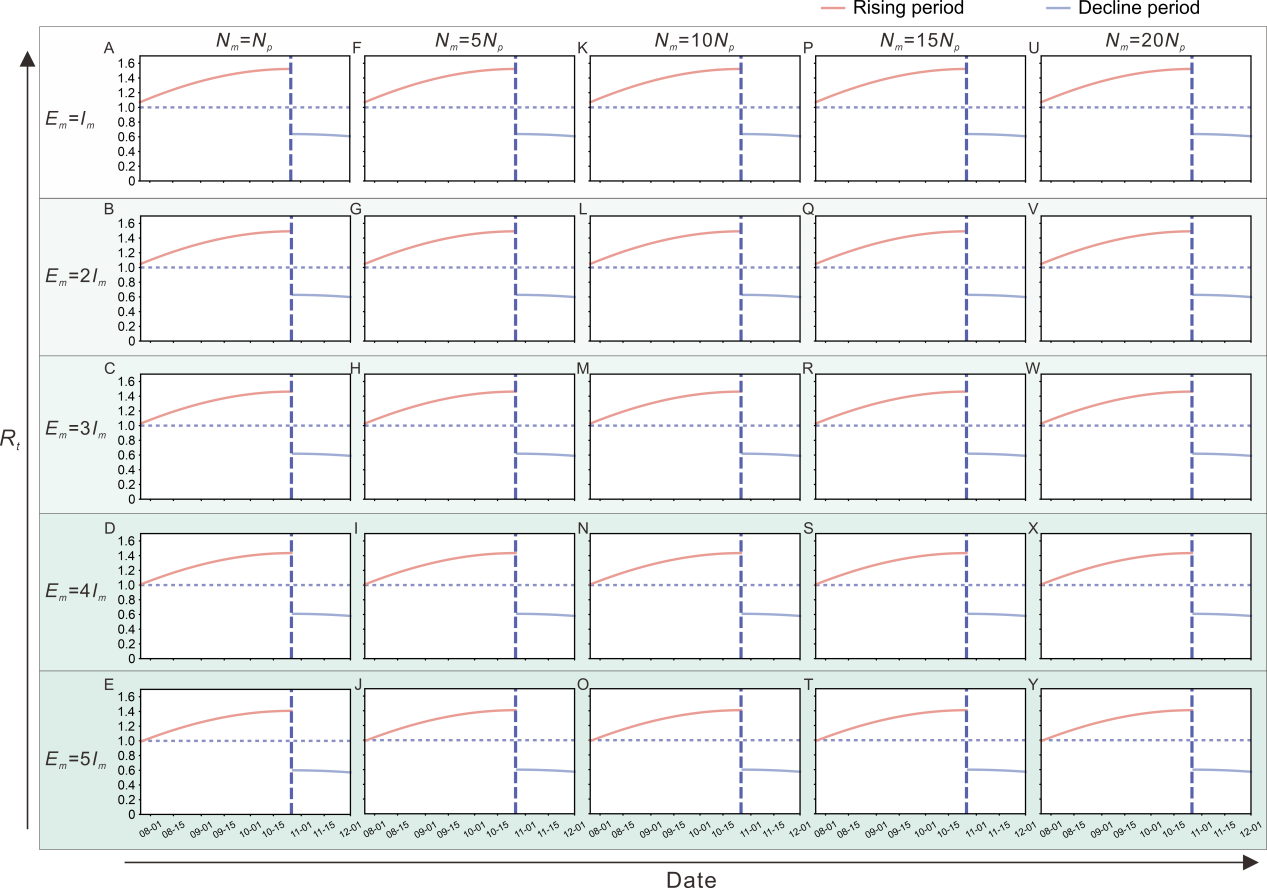


**Fig. 3.6 Calculation results of the dengue fever transmissibility in Dehong Dai and Jingpo Autonomous Prefecture, 2020.** (A)-(Y) for scenario 1-25, with segmentation points marked by purple dashed lines.


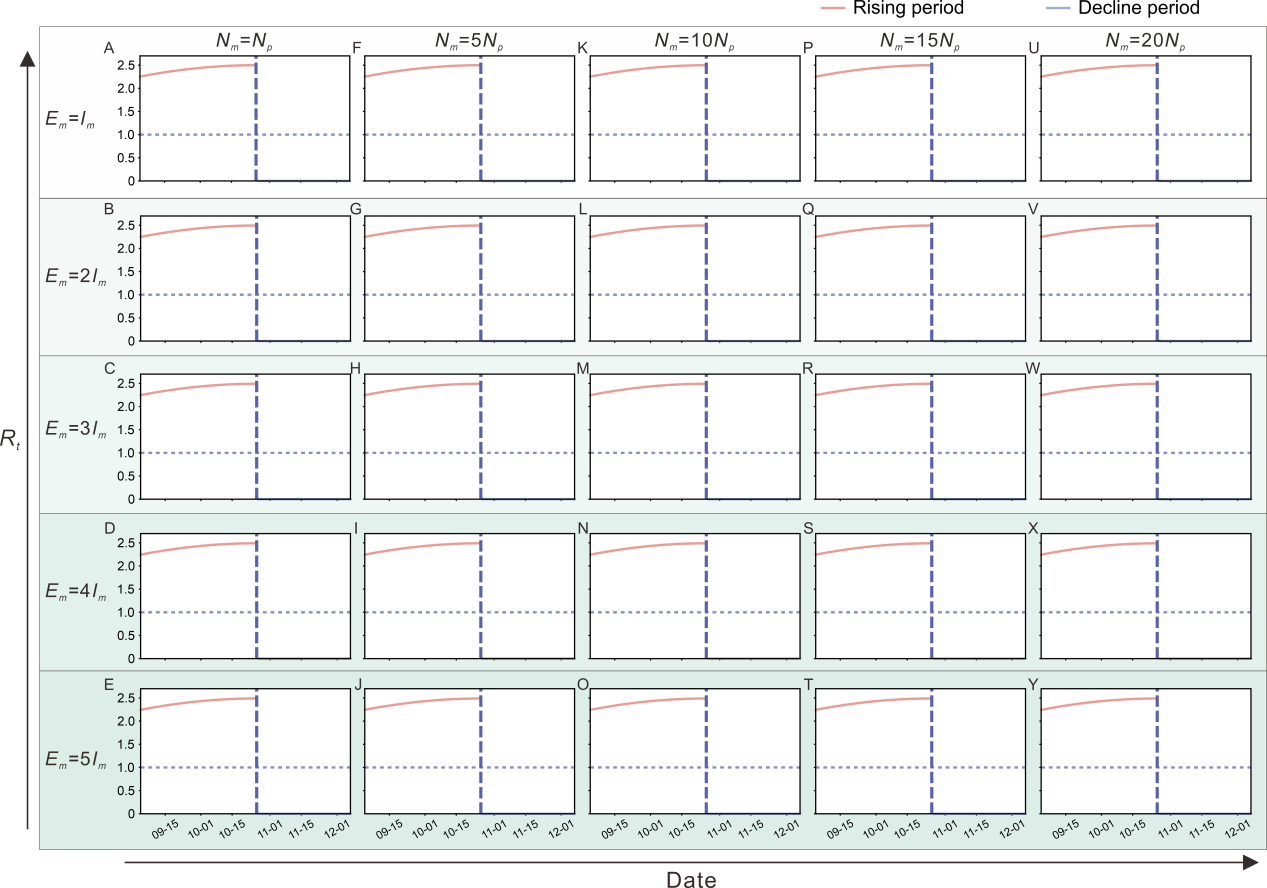


**Fig. 3.7 Calculation results of the dengue fever transmissibility in Dehong Dai and Jingpo Autonomous Prefecture, 2022.** (A)-(Y) for scenario 1-25, with segmentation points marked by purple dashed lines.


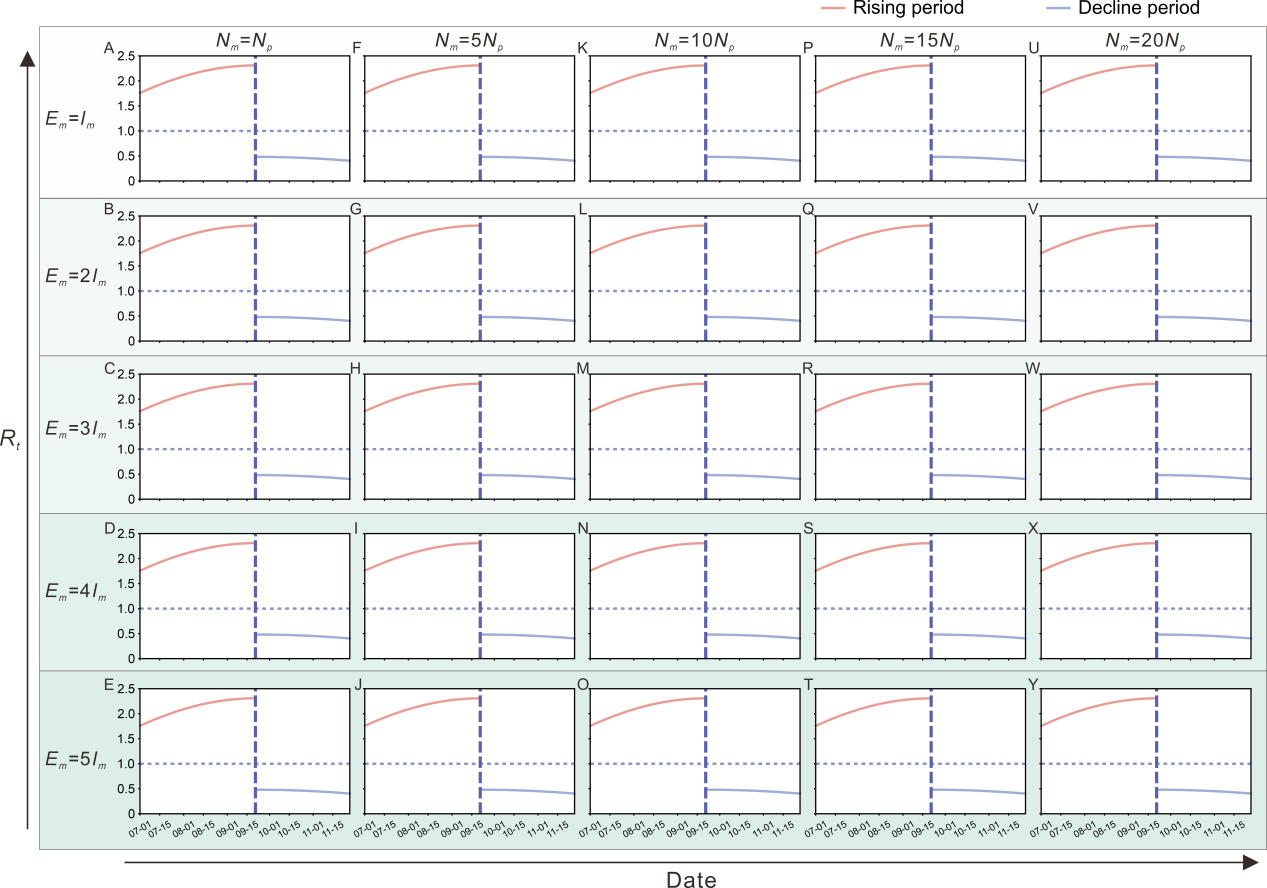


**Fig. 3.8 Calculation results of the dengue fever transmissibility in Dehong Dai and Jingpo Autonomous Prefecture, 2023.** (A)-(Y) for scenario 1-25, with segmentation points marked by purple dashed lines.


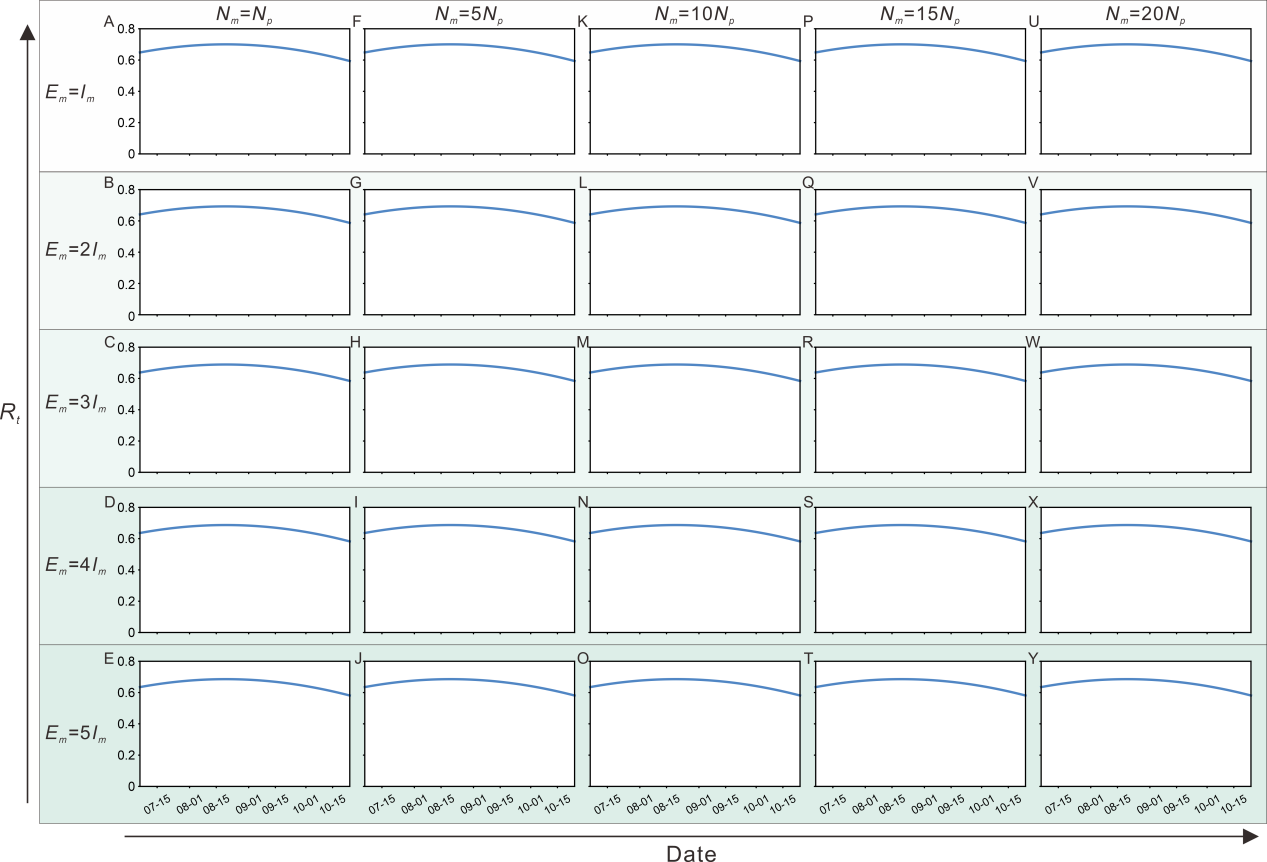


**Fig. 3.9 Calculation results of the dengue fever transmissibility in Lincang City, 2015.** (A)-(Y) for scenario 1-25.


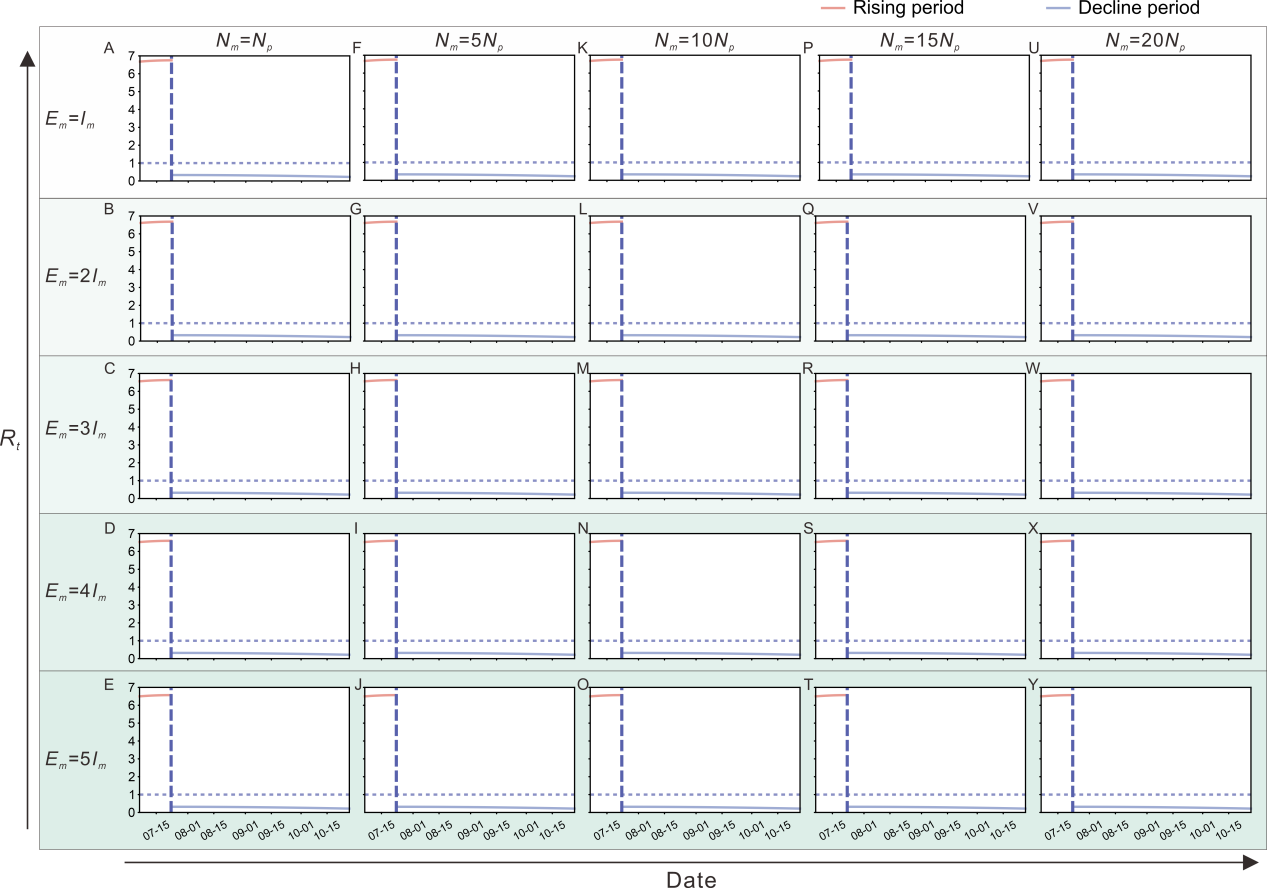


**Fig. 3.10 Calculation results of the dengue fever transmissibility in Lincang City, 2017.** (A)-(Y) for scenario 1-25, with segmentation points marked by purple dashed lines.


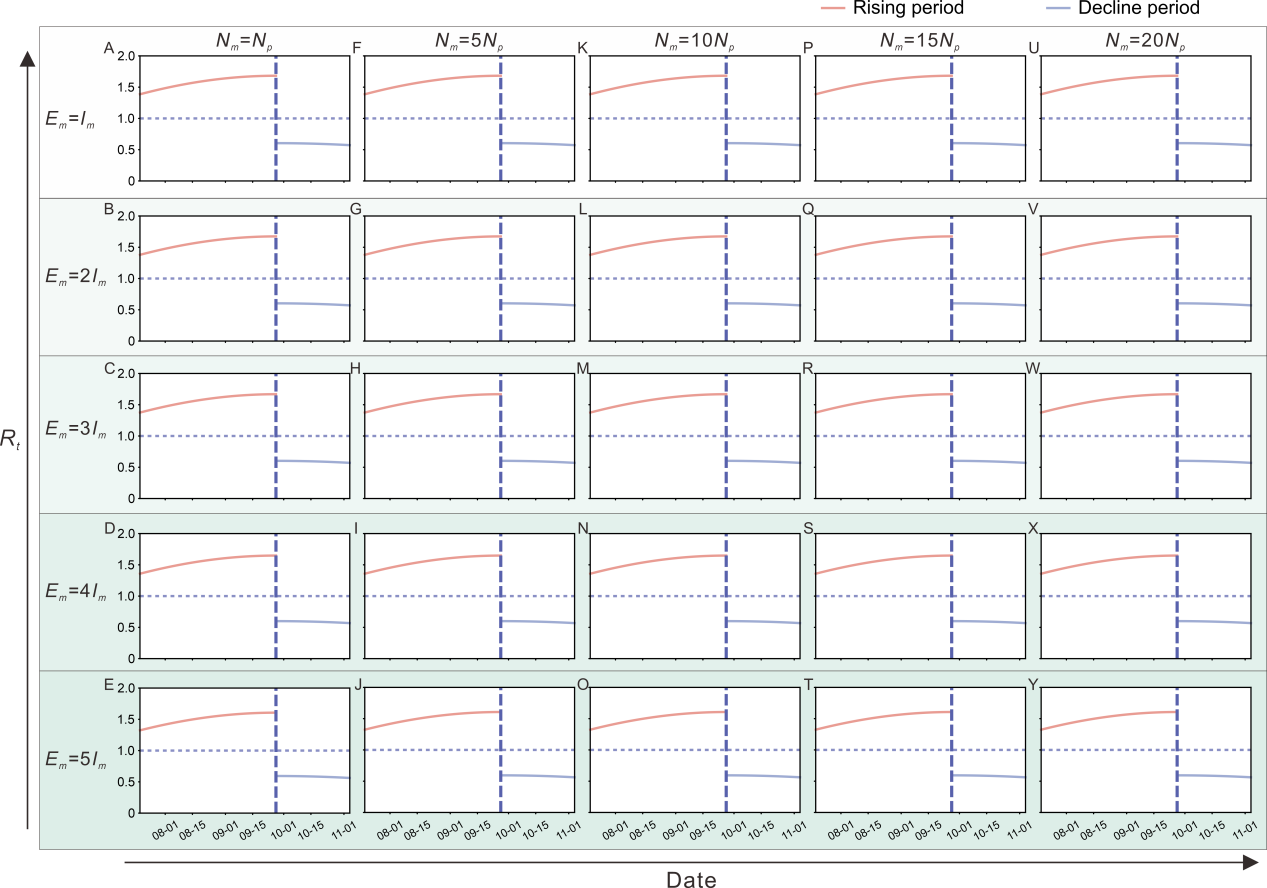


**Fig. 3.11 Calculation results of the dengue fever transmissibility in Lincang City, 2019.** (A)-(Y) for scenario 1-25, with segmentation points marked by purple dashed lines.


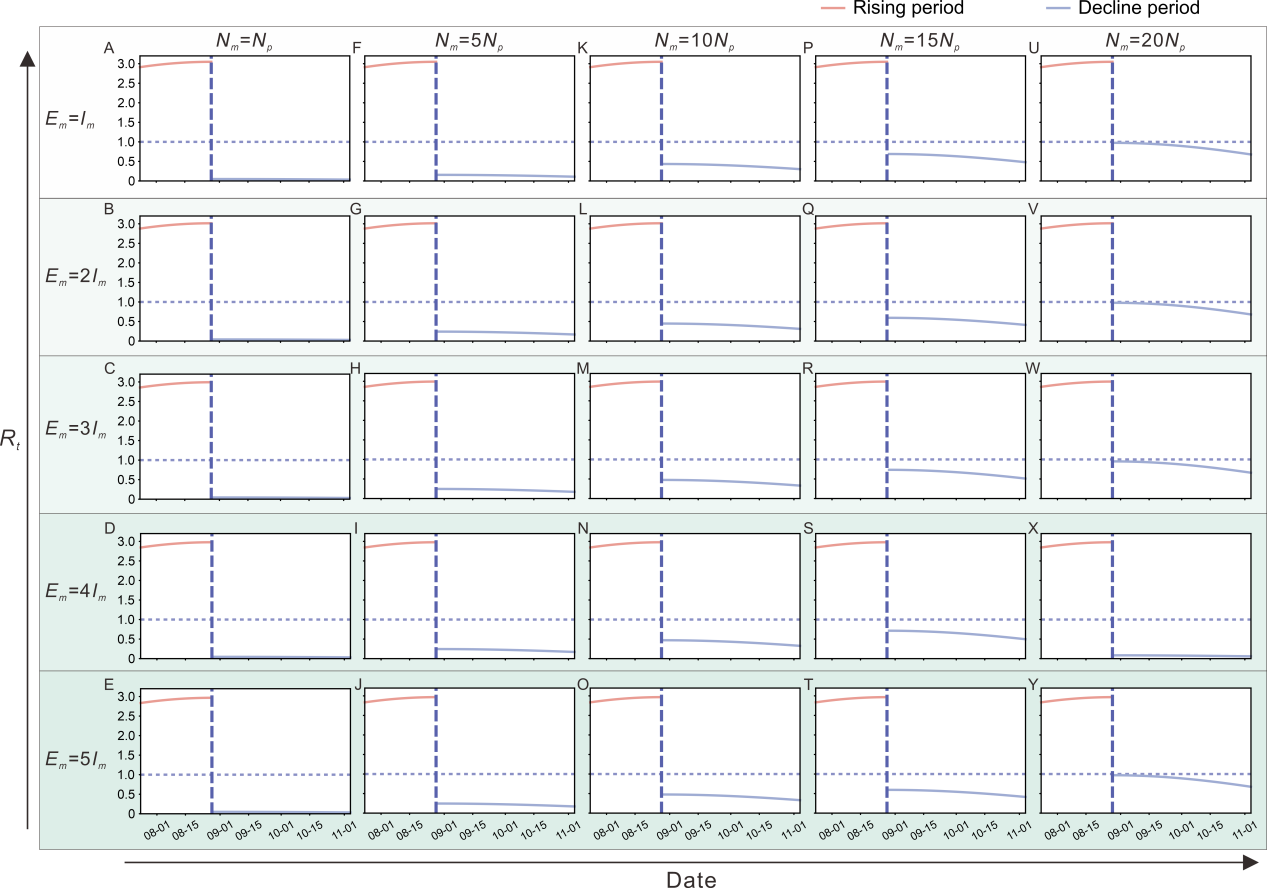


**Fig. 3.12 Calculation results of the dengue fever transmissibility in Lincang City, 2023.** (A)-(Y) for scenario 1-25, with segmentation points marked by purple dashed lines.

# Dengue fever human-to-mosquito transmissibility *R_t_*_(_*_hm_*_)_ for all scenarios


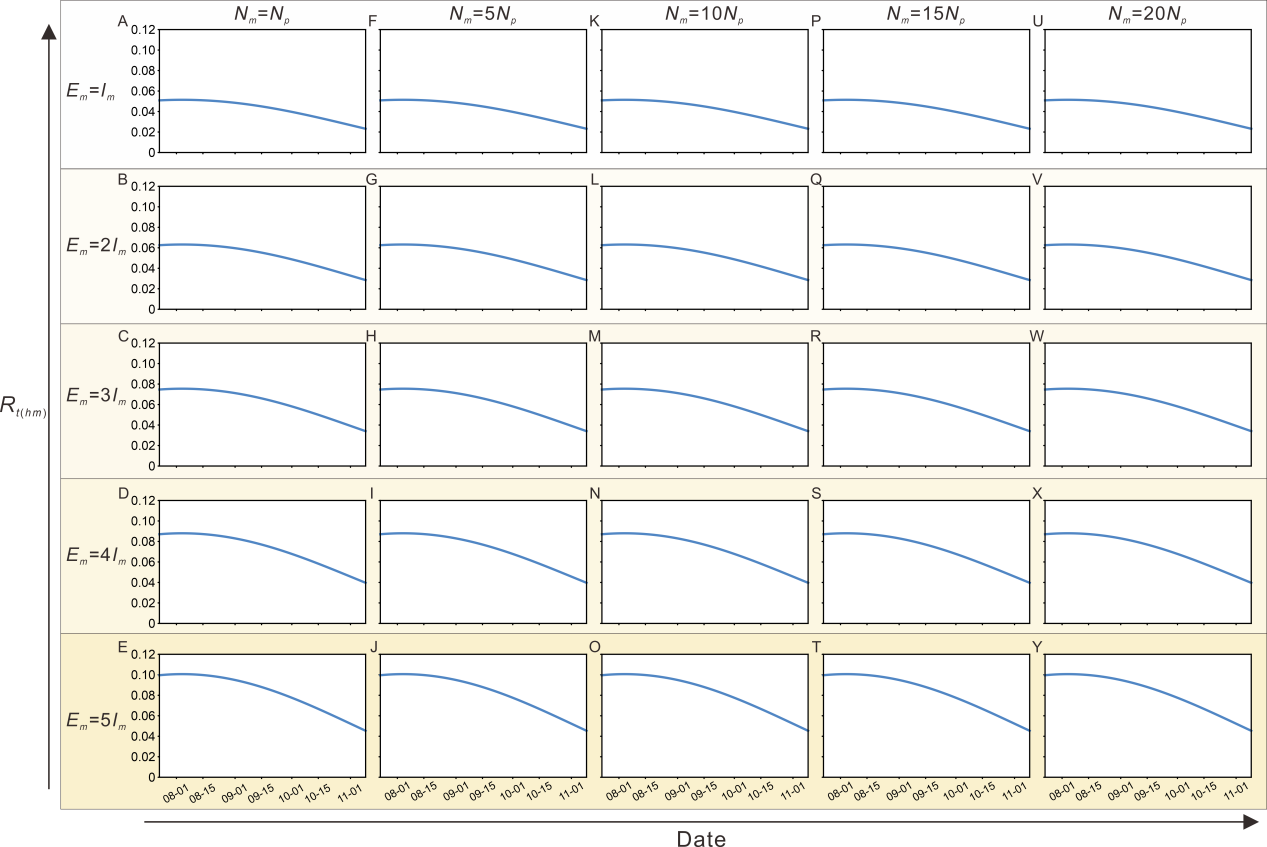


**Fig. 4.1 Calculation results of the human-to-mosquito transmissibility in Dehong Dai and Jingpo Autonomous Prefecture, 2014.** (A)-(Y) for scenario 1-25.


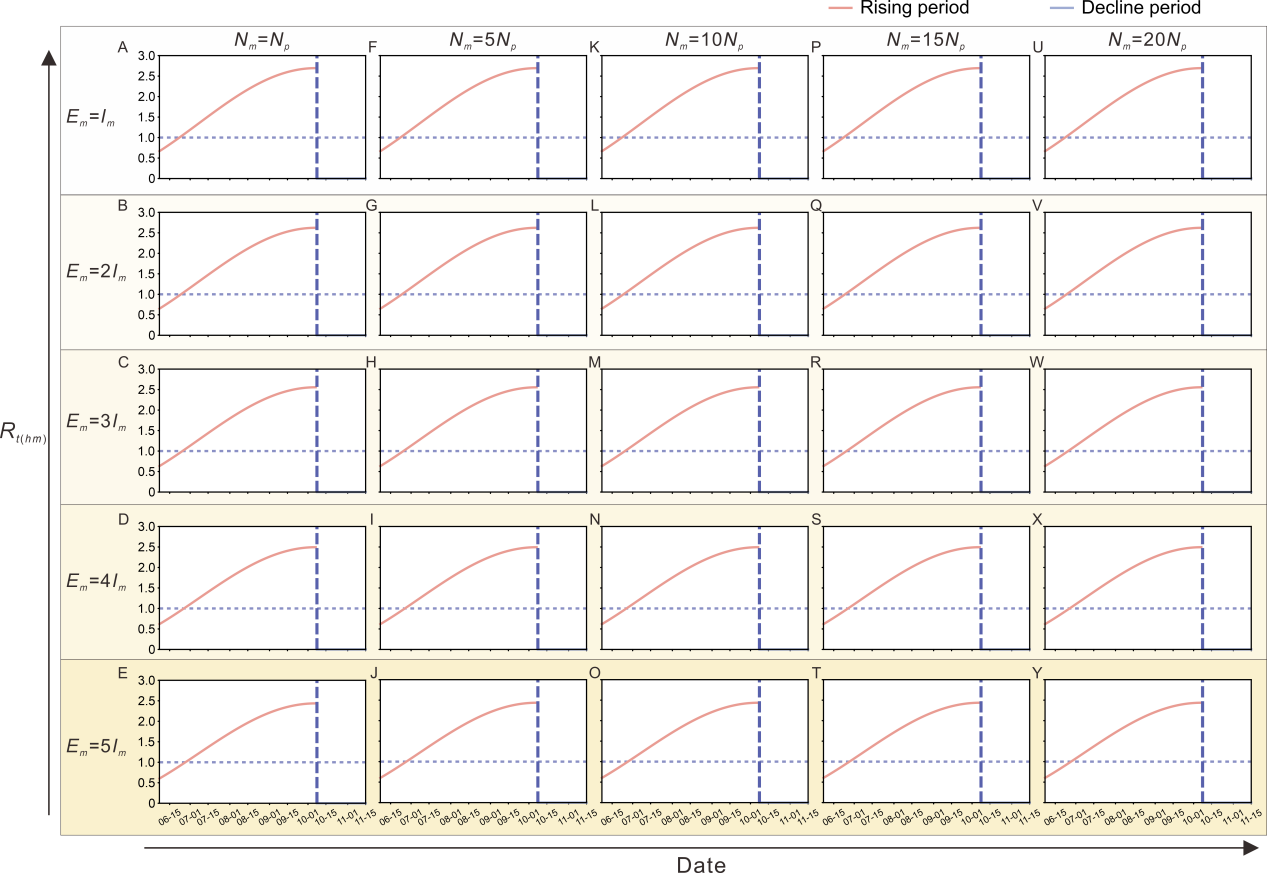


**Fig. 4.2 Calculation results of the human-to-mosquito transmissibility in Dehong Dai and Jingpo Autonomous Prefecture, 2015.** (A)-(Y) for scenario 1-25, with segmentation points marked by purple dashed lines.


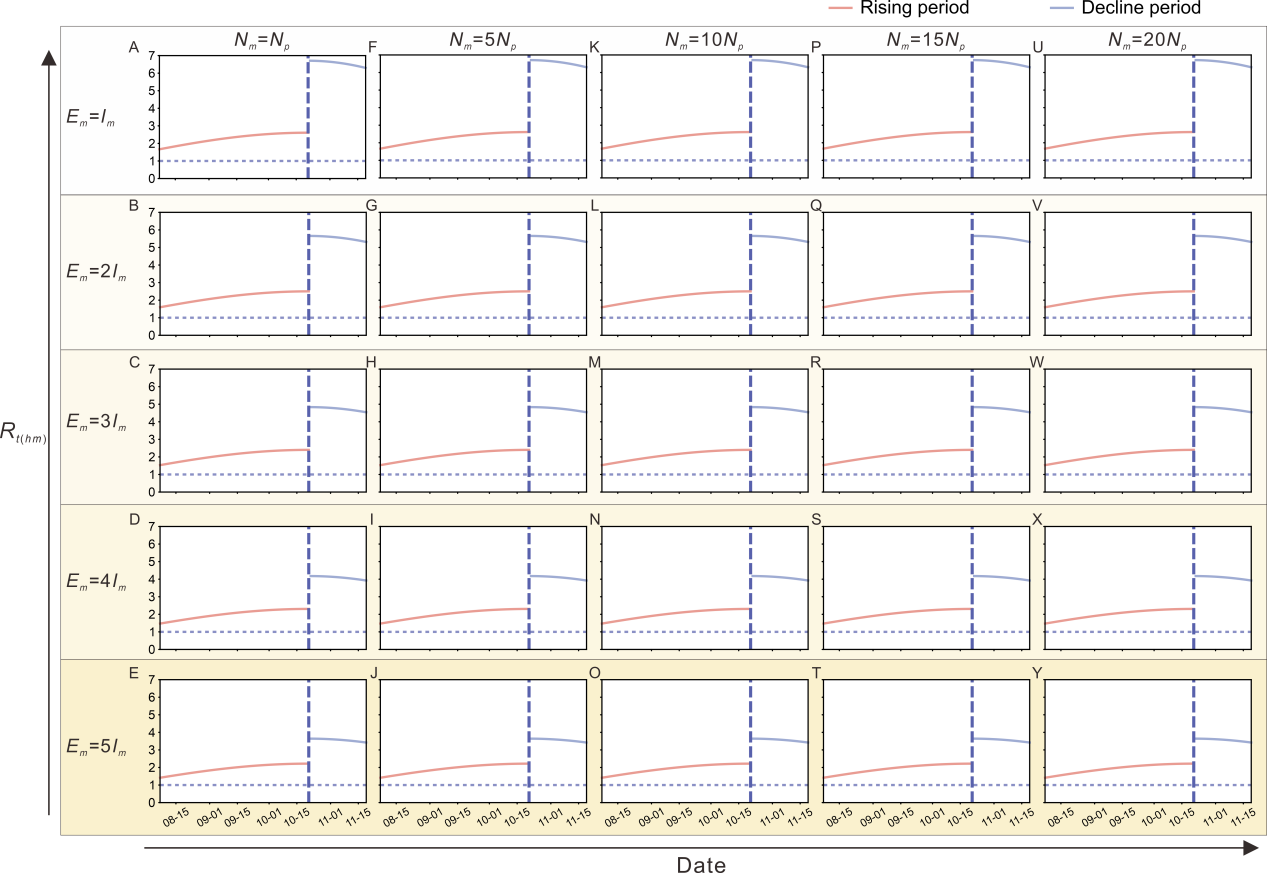


**Fig. 4.3 Calculation results of the human-to-mosquito transmissibility in Dehong Dai and Jingpo Autonomous Prefecture, 2016.** (A)-(Y) for scenario 1-25, with segmentation points marked by purple dashed lines.


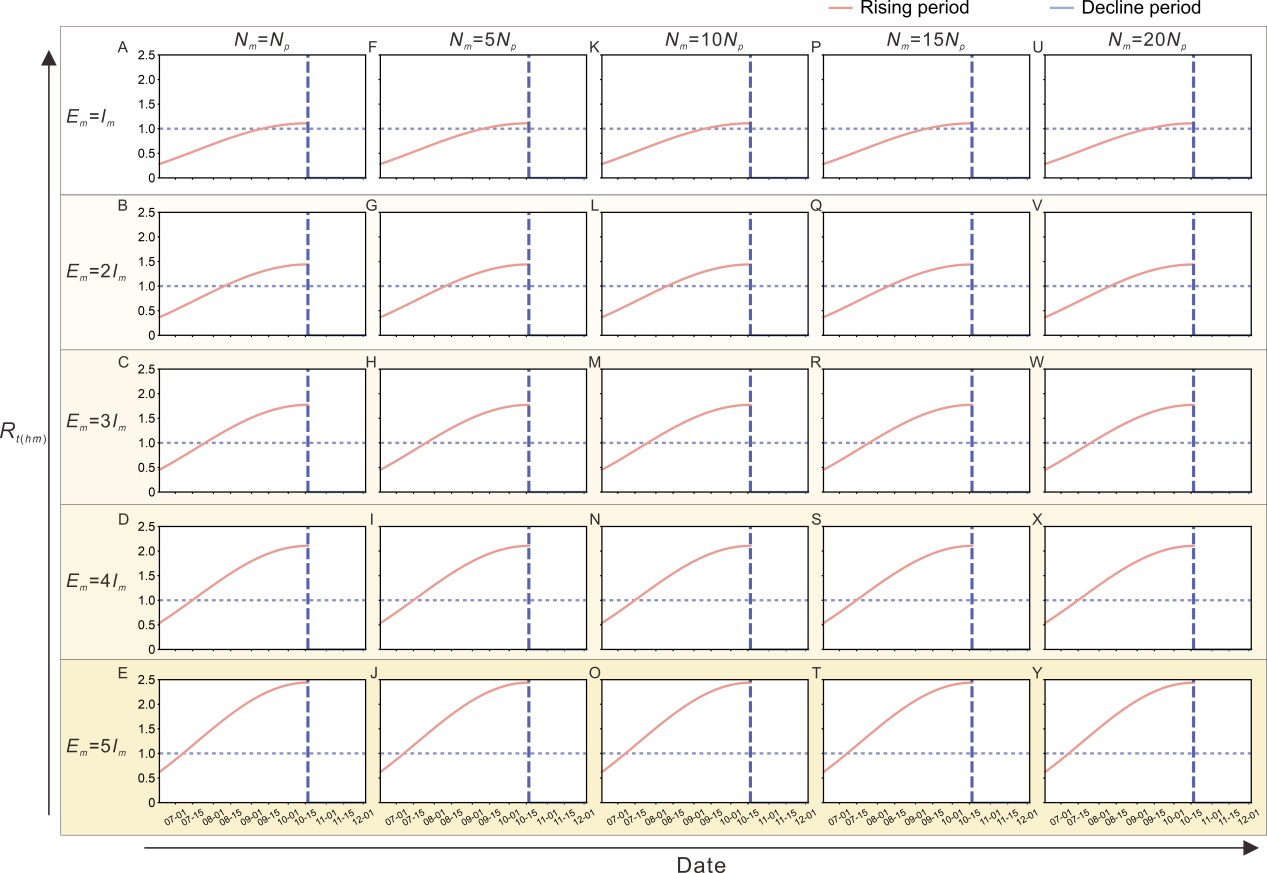


**Fig. 4.4 Calculation results of the human-to-mosquito transmissibility in Dehong Dai and Jingpo Autonomous Prefecture, 2017.** (A)-(Y) for scenario 1-25, with segmentation points marked by purple dashed lines.


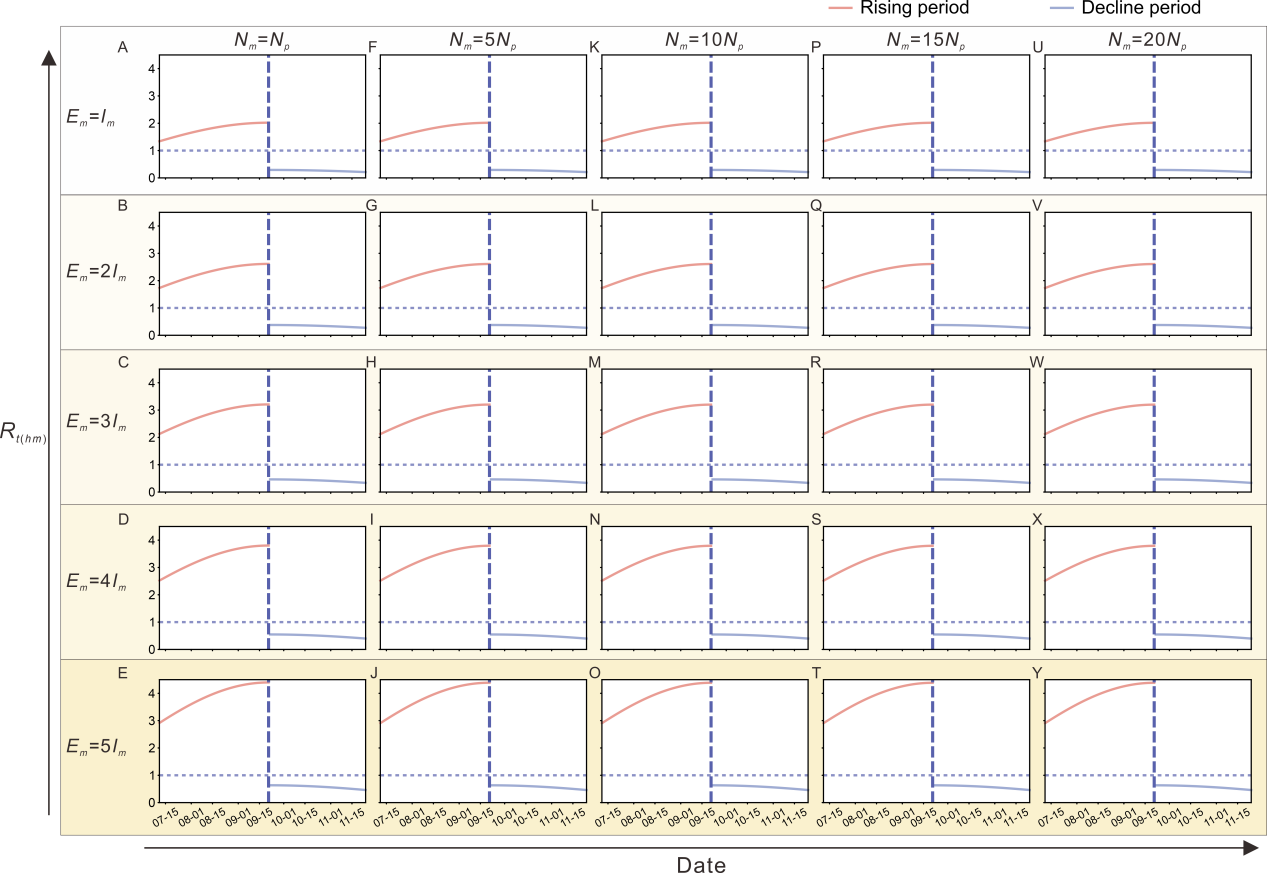


**Fig. 4.5 Calculation results of the human-to-mosquito transmissibility in Dehong Dai and Jingpo Autonomous Prefecture, 2019.** (A)-(Y) for scenario 1-25, with segmentation points marked by purple dashed lines.


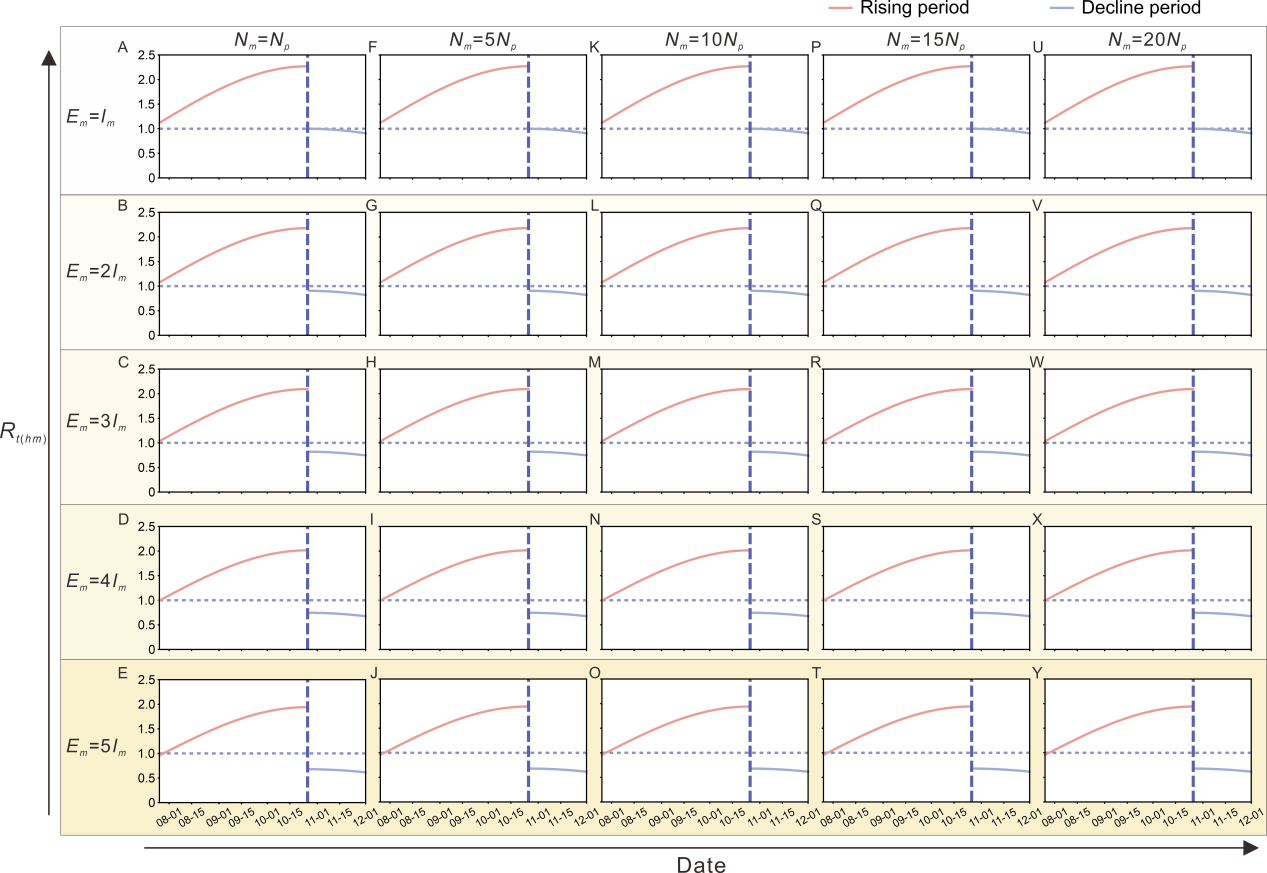


**Fig. 4.6 Calculation results of the human-to-mosquito transmissibility in Dehong Dai and Jingpo Autonomous Prefecture, 2020.** (A)-(Y) for scenario 1-25, with segmentation points marked by purple dashed lines.


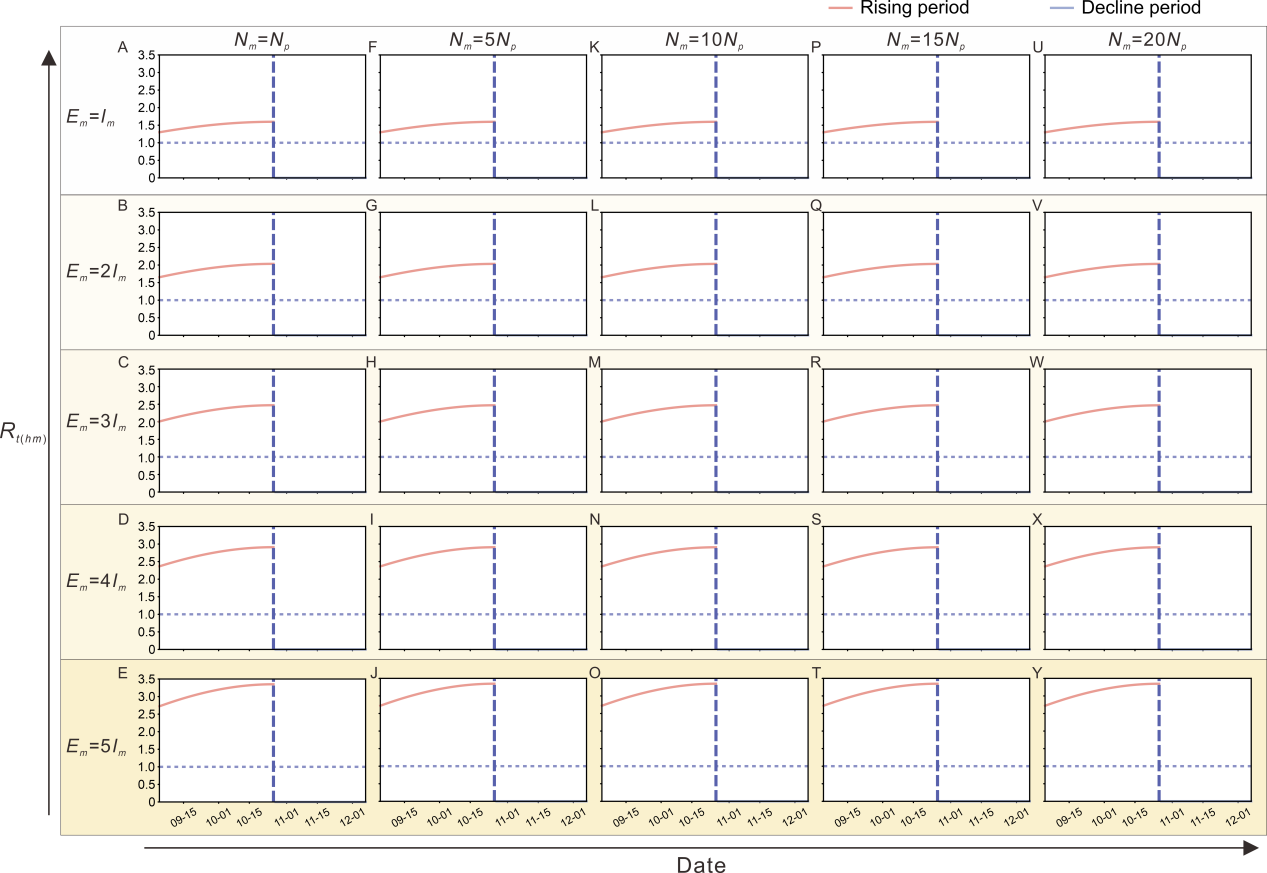


**Fig. 4.7 Calculation results of the human-to-mosquito transmissibility in Dehong Dai and Jingpo Autonomous Prefecture, 2022.** (A)-(Y) for scenario 1-25, with segmentation points marked by purple dashed lines.


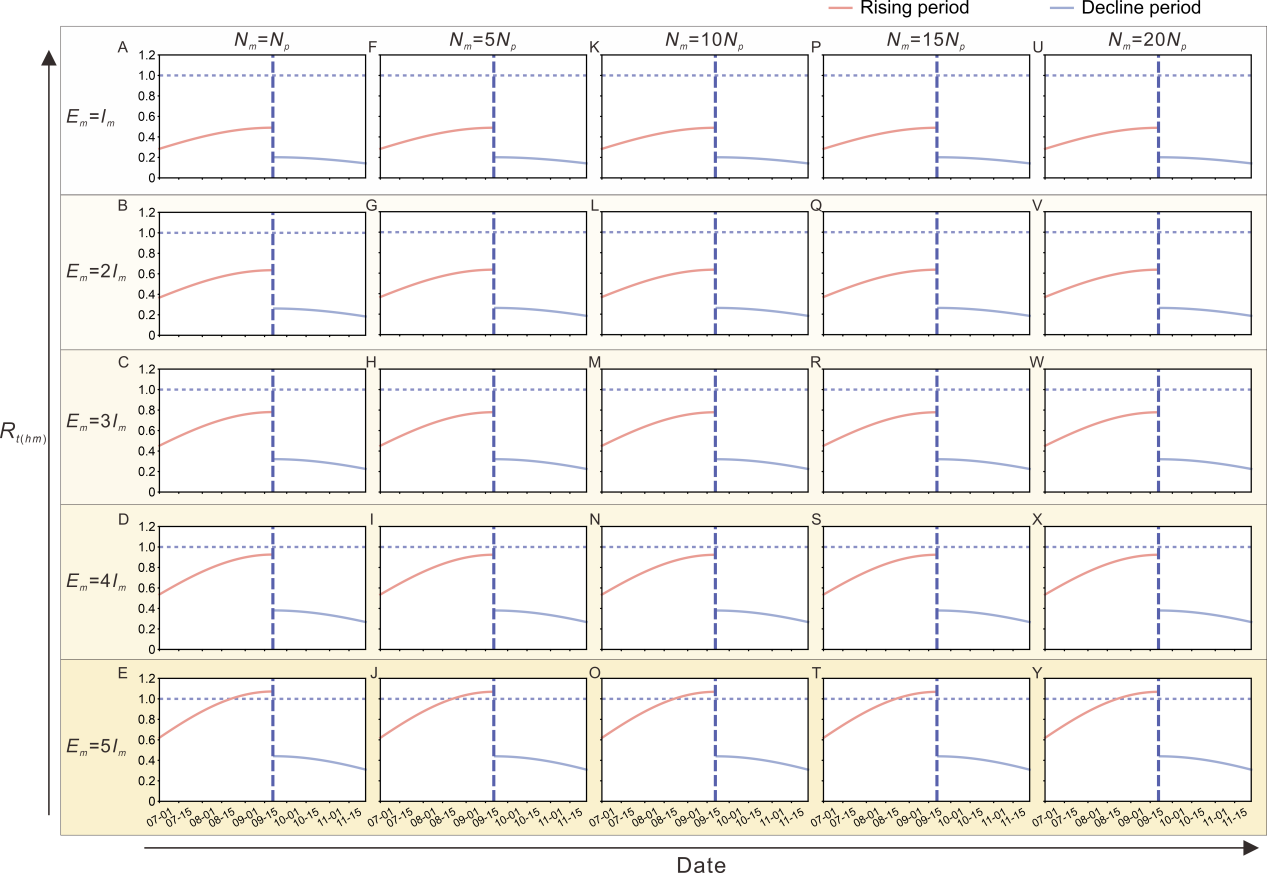


**Fig. 4.8 Calculation results of the human-to-mosquito transmissibility in Dehong Dai and Jingpo Autonomous Prefecture, 2023.** (A)-(Y) for scenario 1-25, with segmentation points marked by purple dashed lines.


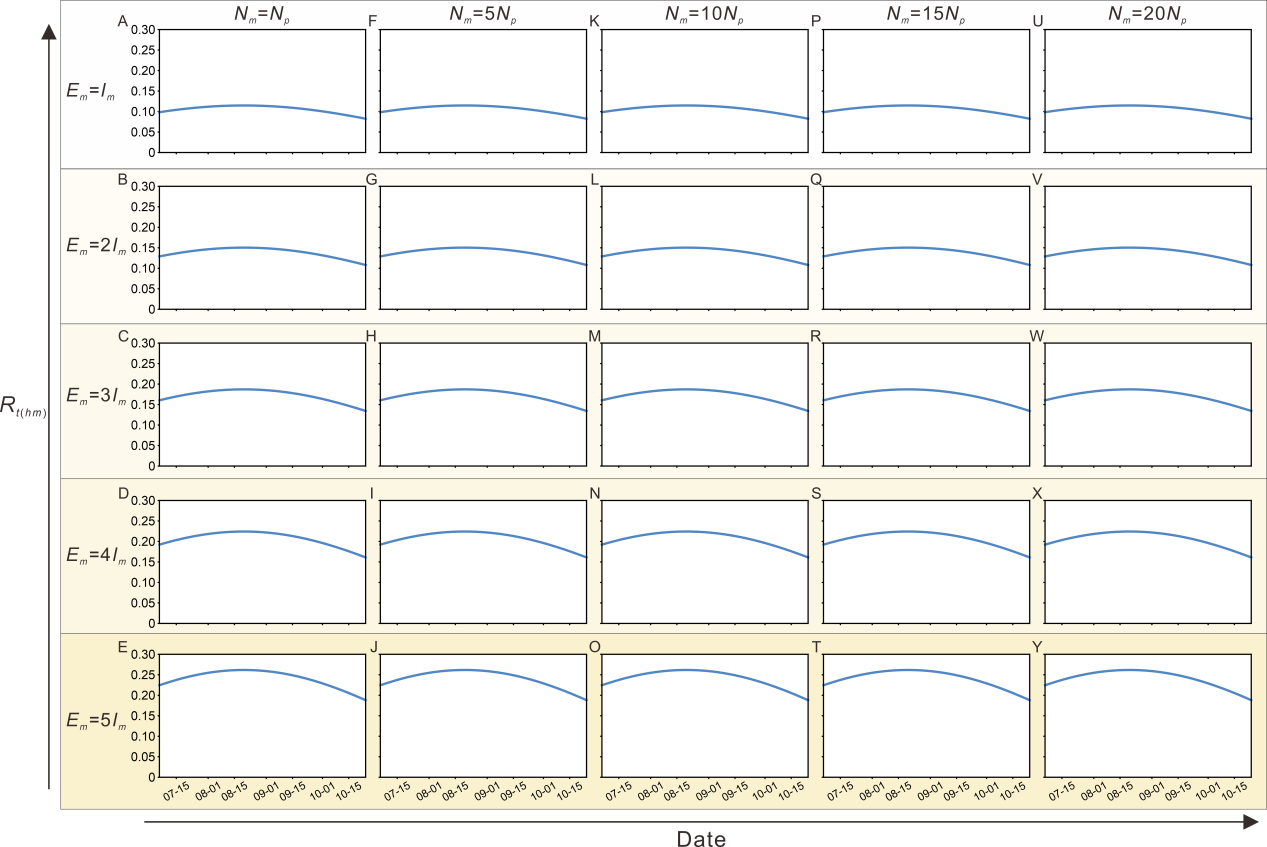


**Fig. 4.9 Calculation results of the human-to-mosquito transmissibility in Lincang City, 2015.** (A)-(Y) for scenario 1-25.


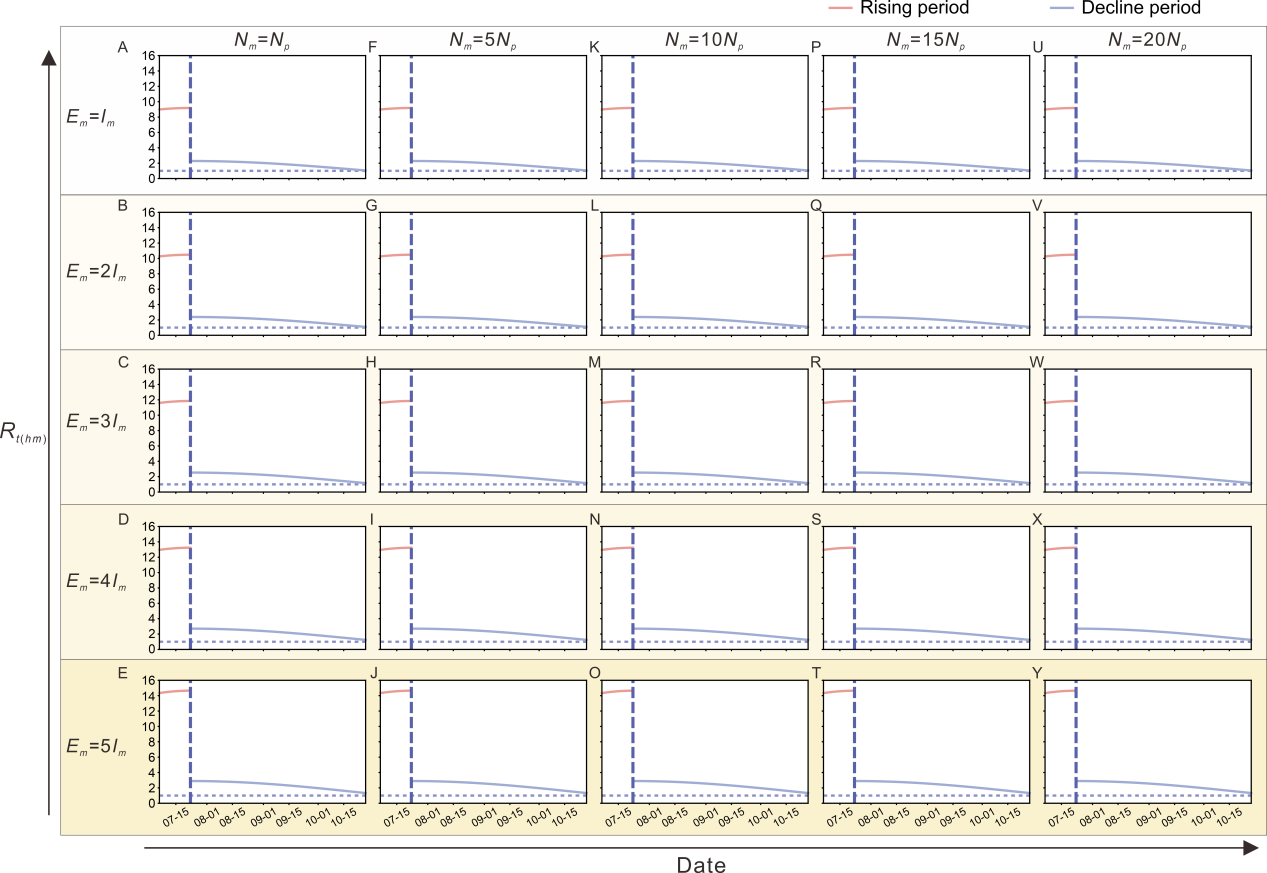


**Fig. 4.10 Calculation results of the human-to-mosquito transmissibility in Lincang City, 2017.** (A)-(Y) for scenario 1-25, with segmentation points marked by purple dashed lines


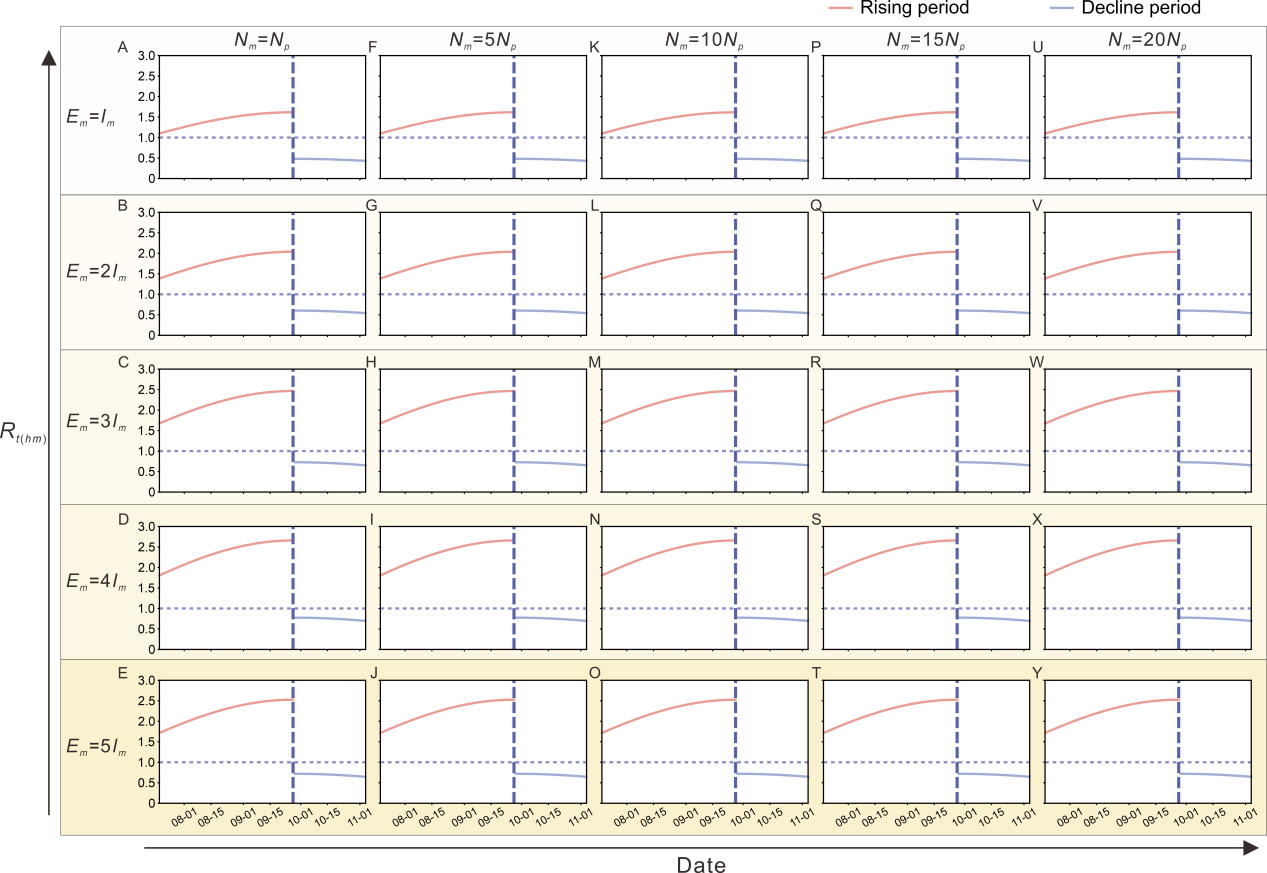


**Fig. 4.11 Calculation results of the human-to-mosquito transmissibility in Lincang City, 2019.** (A)-(Y) for scenario 1-25, with segmentation points marked by purple dashed lines.


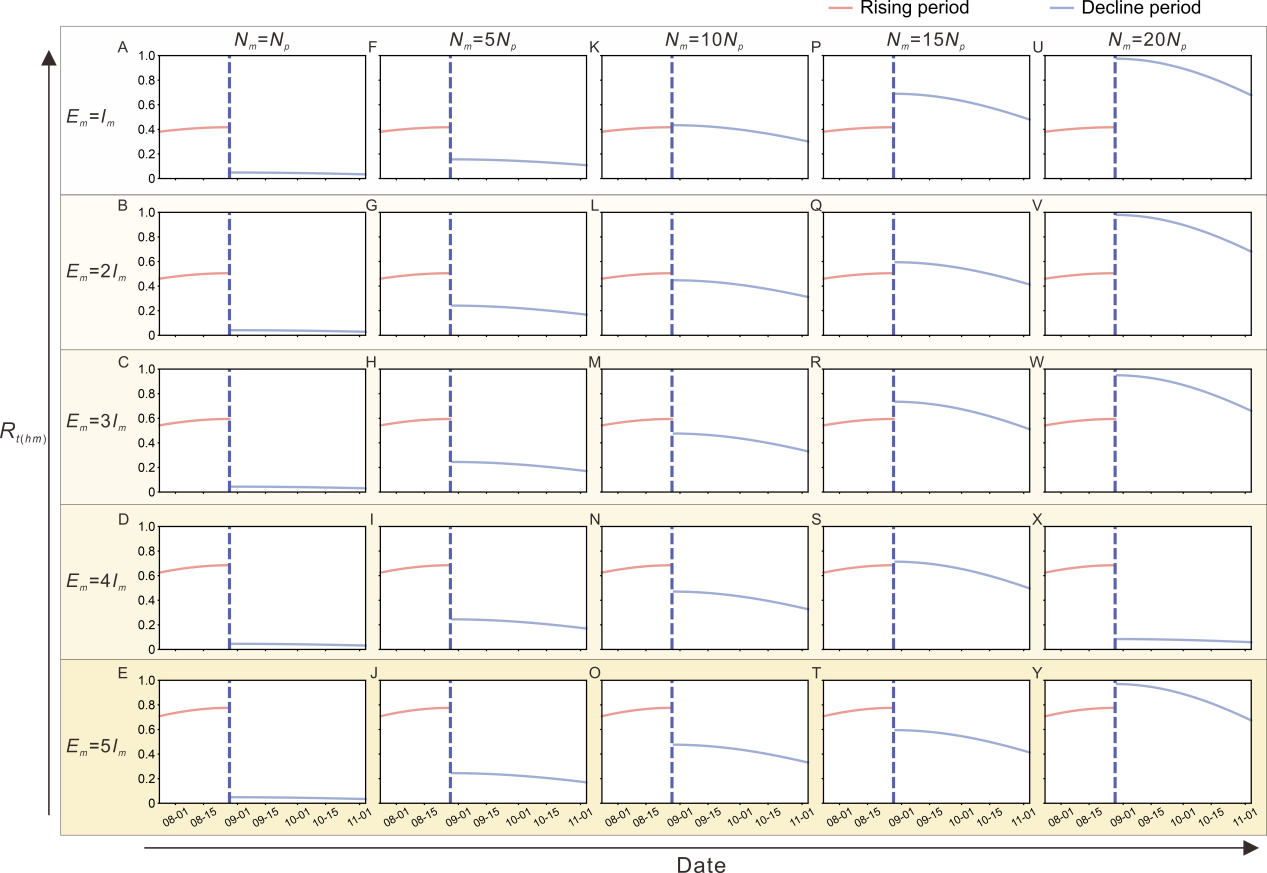


**Fig. 4.12 Calculation results of the human-to-mosquito transmissibility in Lincang City, 2023.** (A)-(Y) for scenario 1-25, with segmentation points marked by purple dashed lines.

# Uncertainty analysis

We performed 5000 Monte Carlo simulations to assess uncertainty in transmission capacity. Transmission capacity values of zero during the epidemic decline phase in some years reflect values approximating zero due to their extremely small magnitude.

## 5.1 *R_t_* results


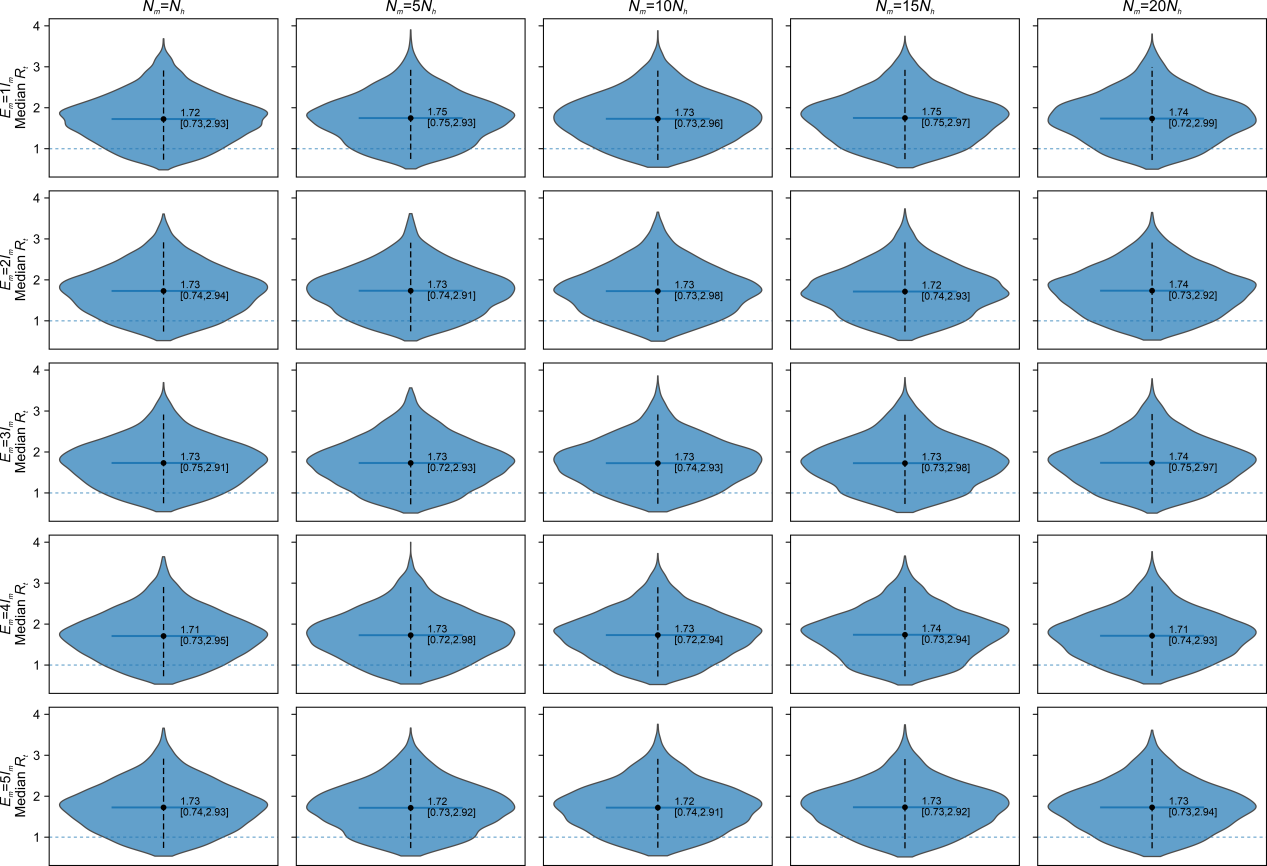


**Fig. 5.1 Monte Carlo simulation results of the median *R_t_* during the rising period for all scenarios in DH, 2017.**

**
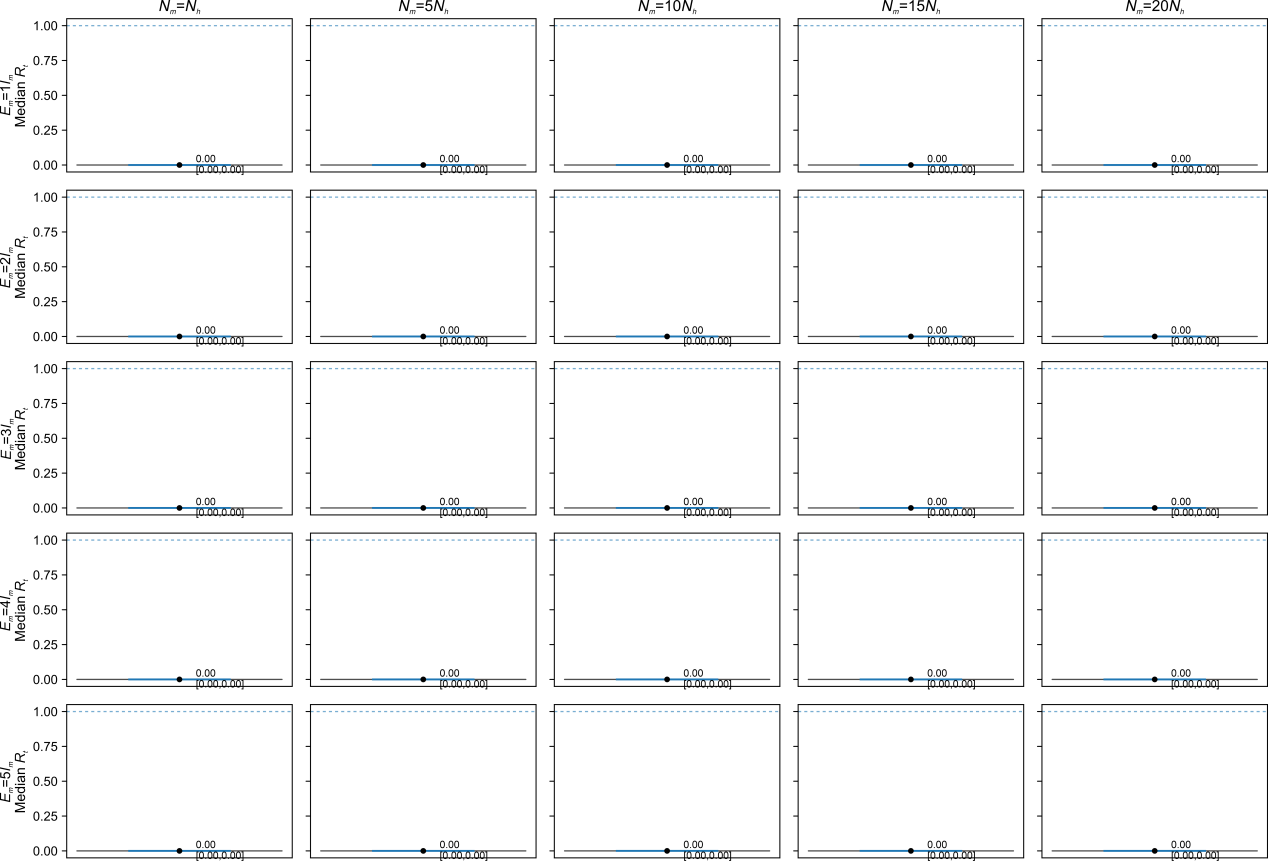
**

**Fig. 5.2 Monte Carlo simulation results of the median *R_t_* during the decline period for all scenarios in DH, 2017.**


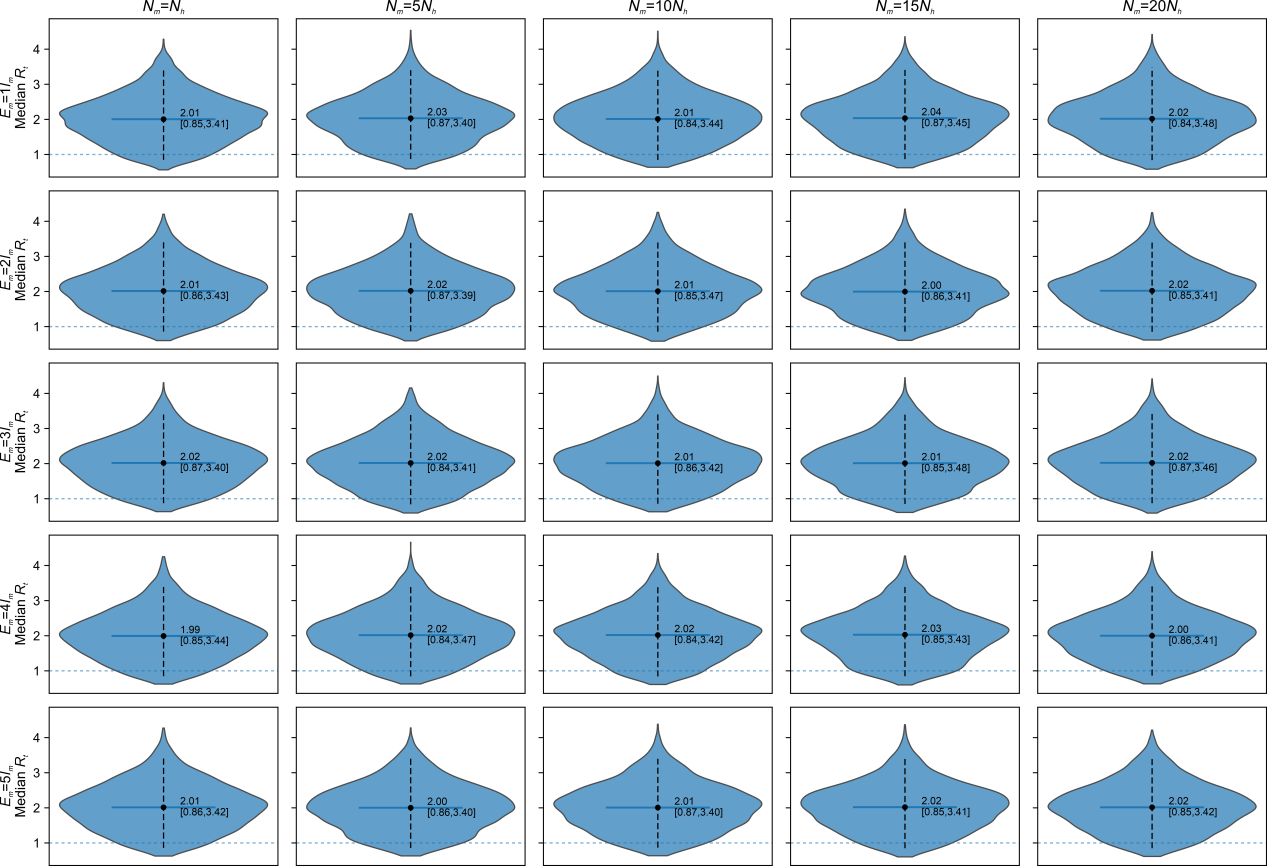


**Fig. 5.3 Monte Carlo simulation results of the median *R_t_* during the rising period for all scenarios in DH, 2019.**


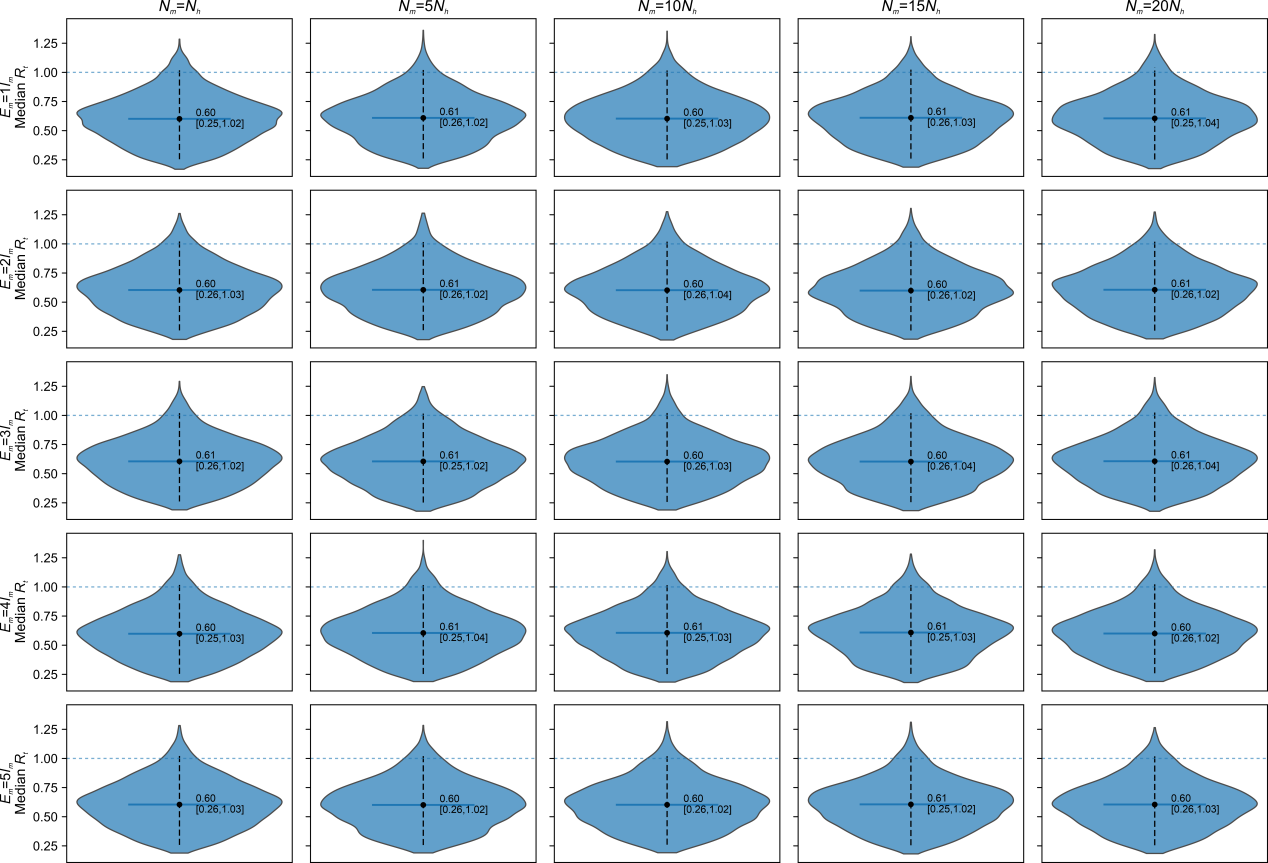


**Fig. 5.4 Monte Carlo simulation results of the median *R_t_* during the decline period for all scenarios in DH, 2019.**


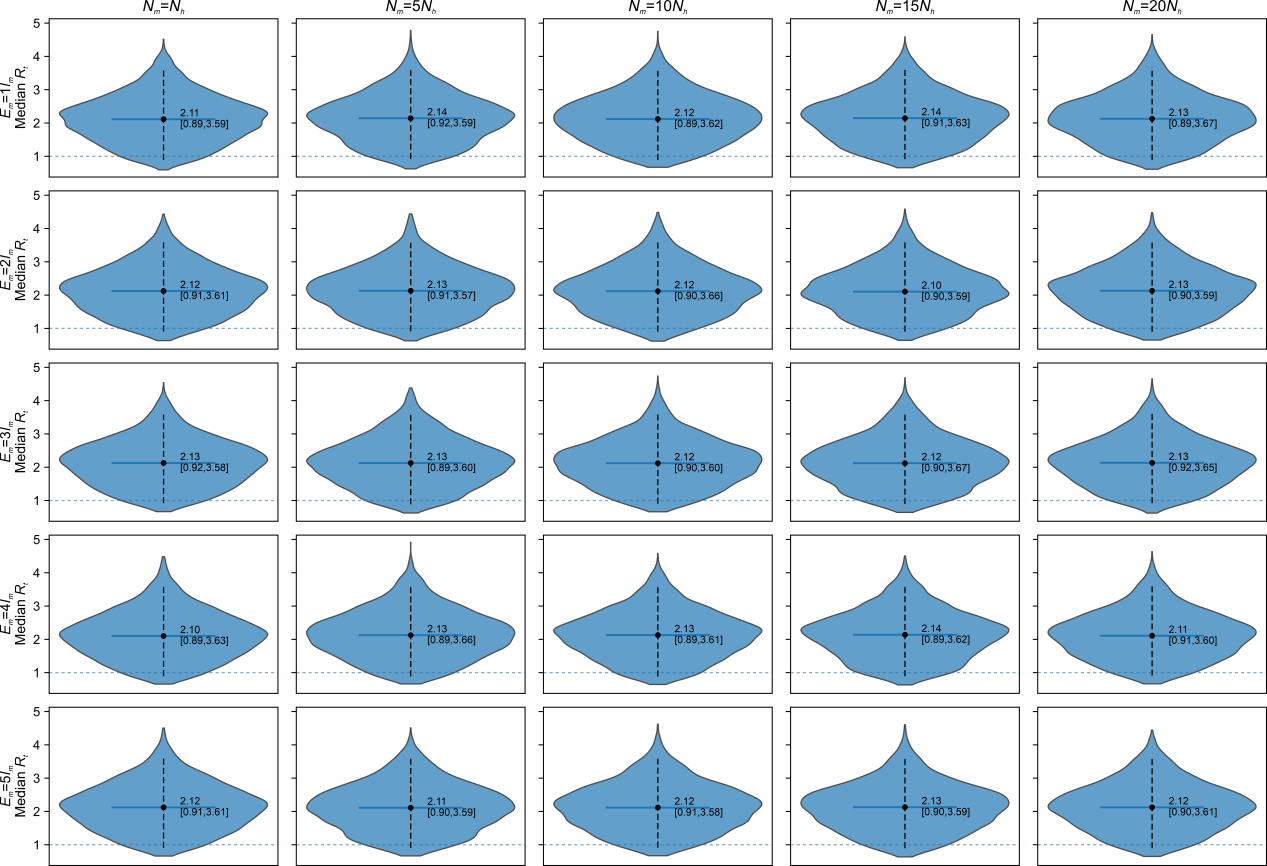


**Fig. 5.5 Monte Carlo simulation results of the median *R_t_* during the rising period for all scenarios in DH, 2023.**


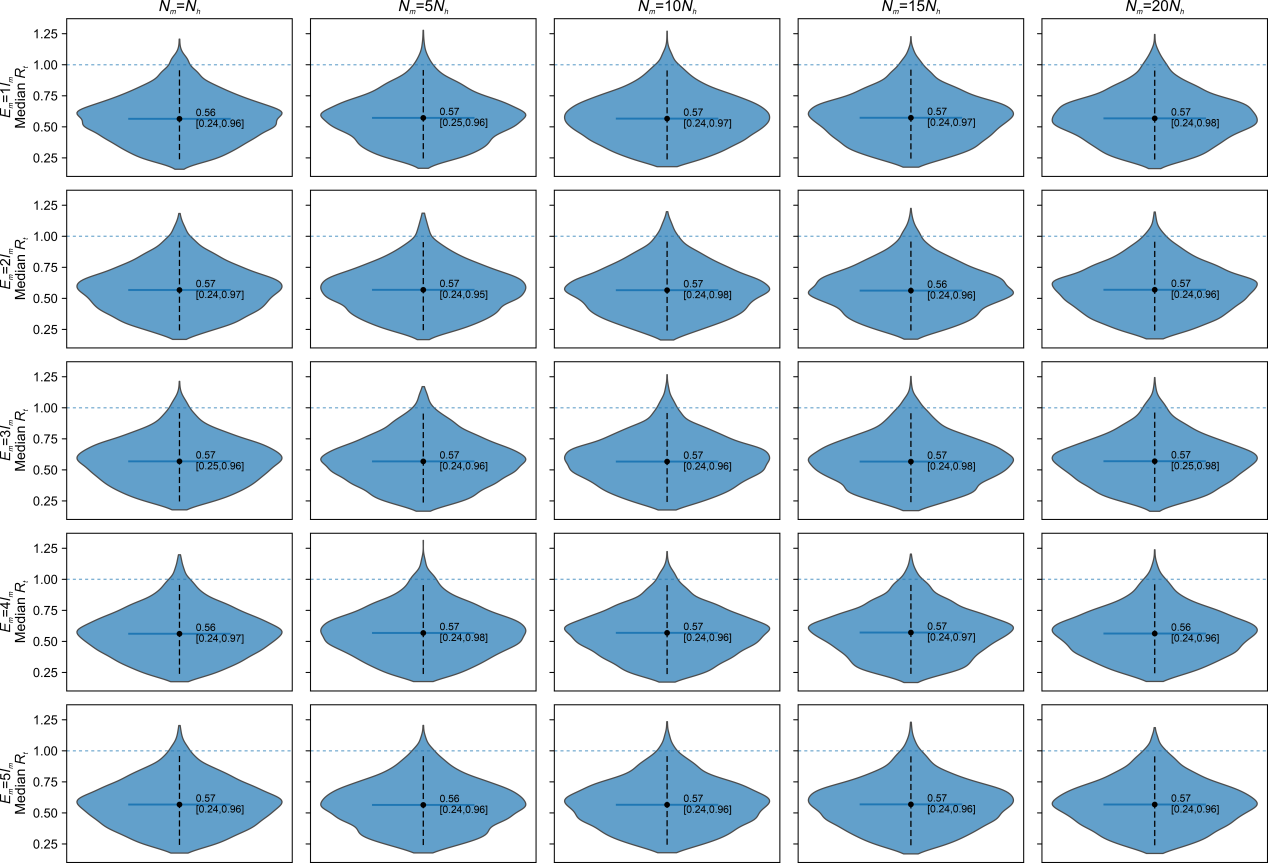


**Fig. 5.6 Monte Carlo simulation results of the median *R_t_* during the decline period for all scenarios in DH, 2023.**


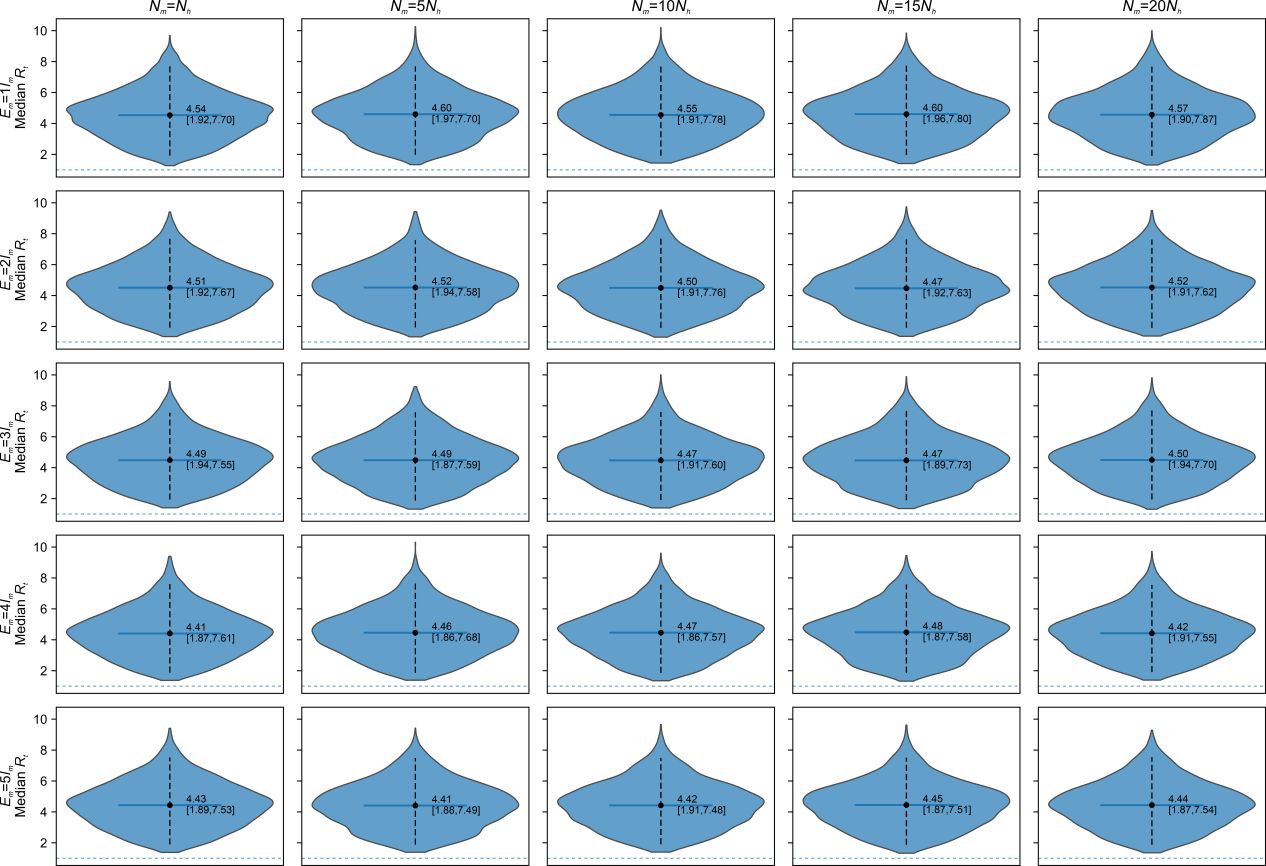


**Fig. 5.7 Monte Carlo simulation results of the median *R_t_* during the rising period for all scenarios in LC, 2017.**

**
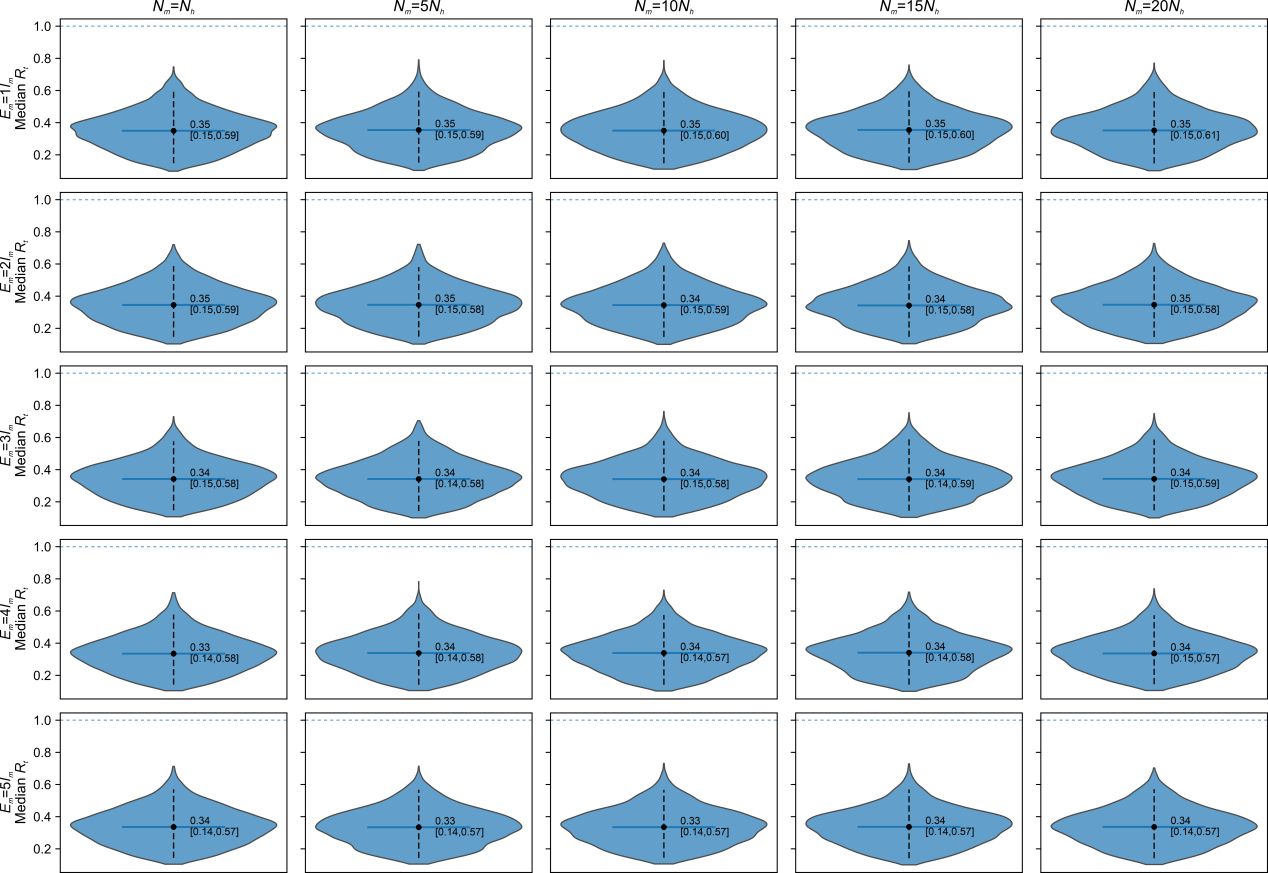
**

**Fig. 5.8 Monte Carlo simulation results of the median *R_t_* during the decline period for all scenarios in LC, 2017.**

**
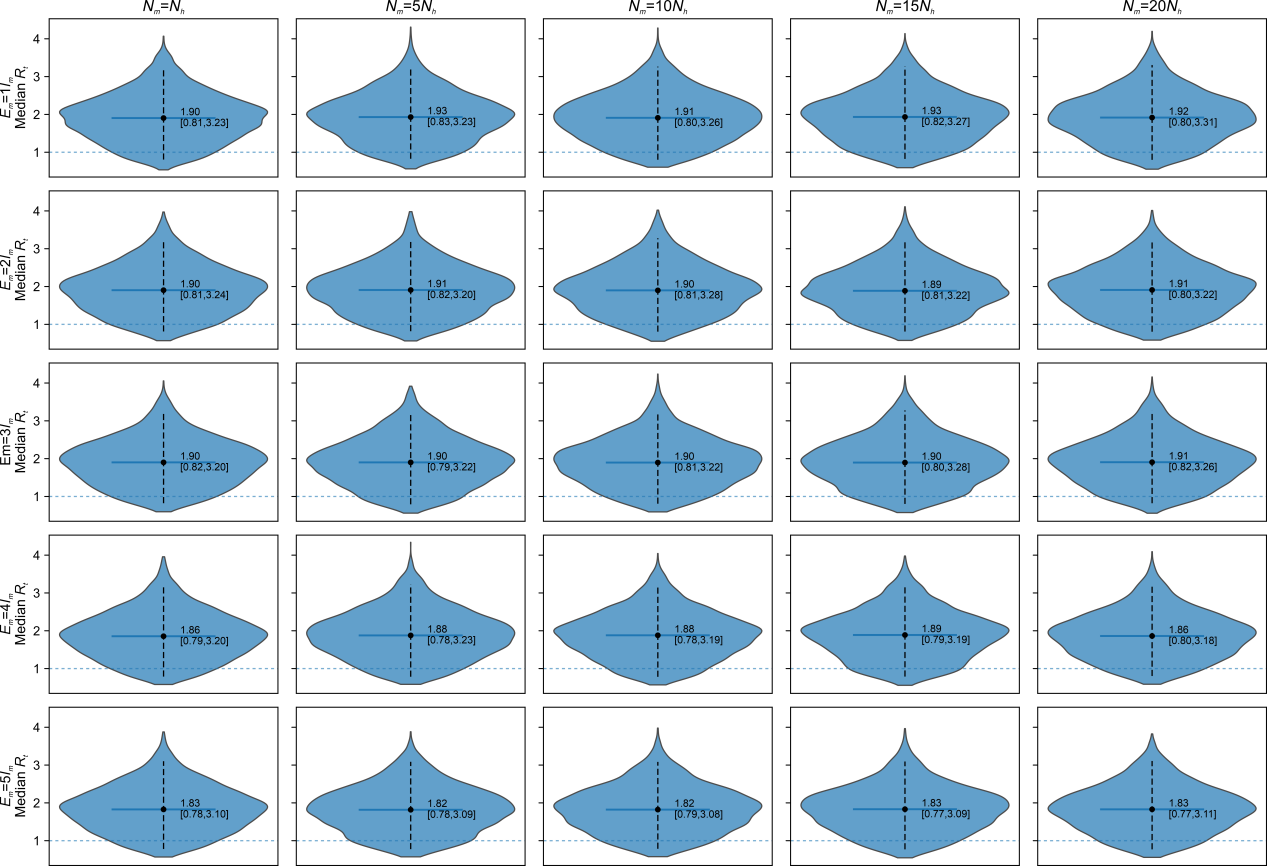
**

**Fig. 5.9 Monte Carlo simulation results of the median *R_t_* during the rising period for all scenarios in LC, 2019.**

**
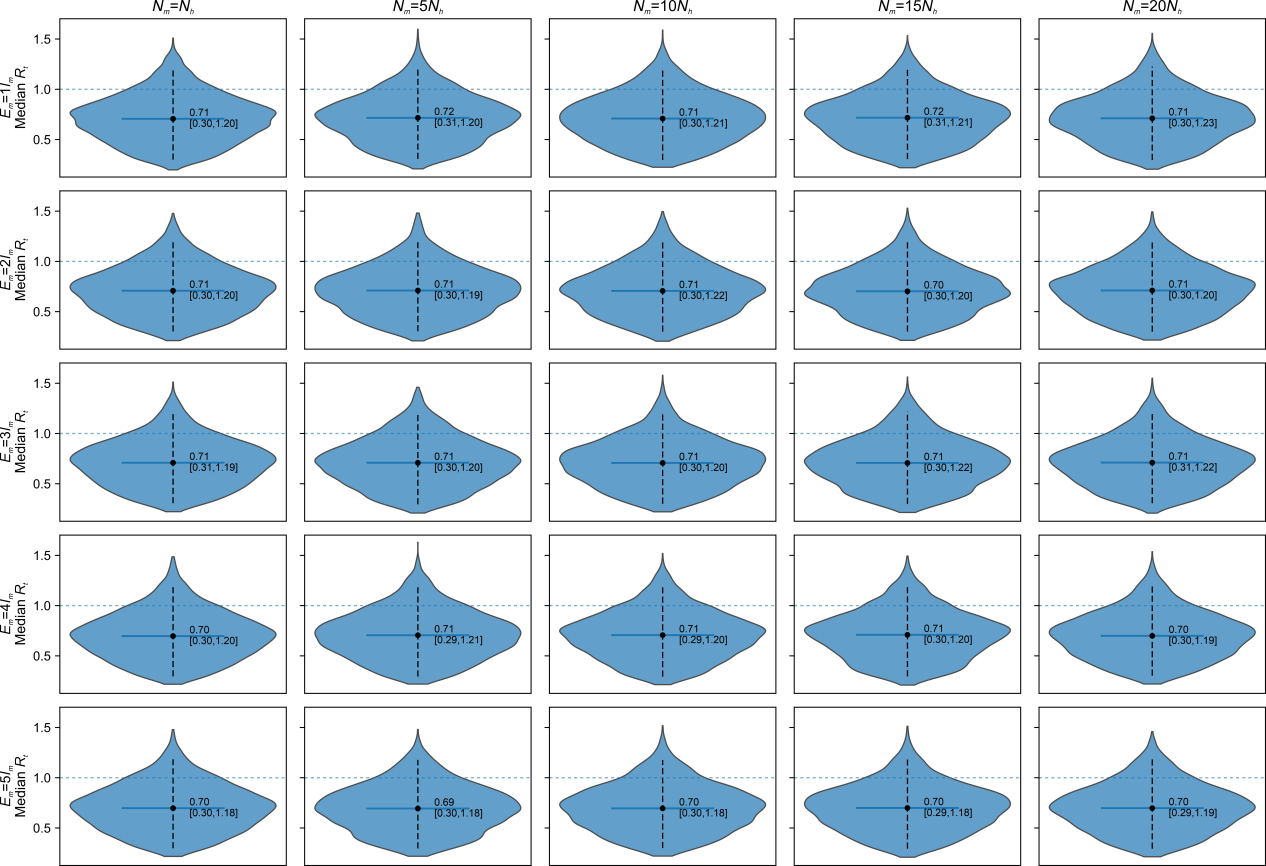
**

**Fig. 5.10 Monte Carlo simulation results of the median *R_t_* during the decline period for all scenarios in LC, 2019.**

**
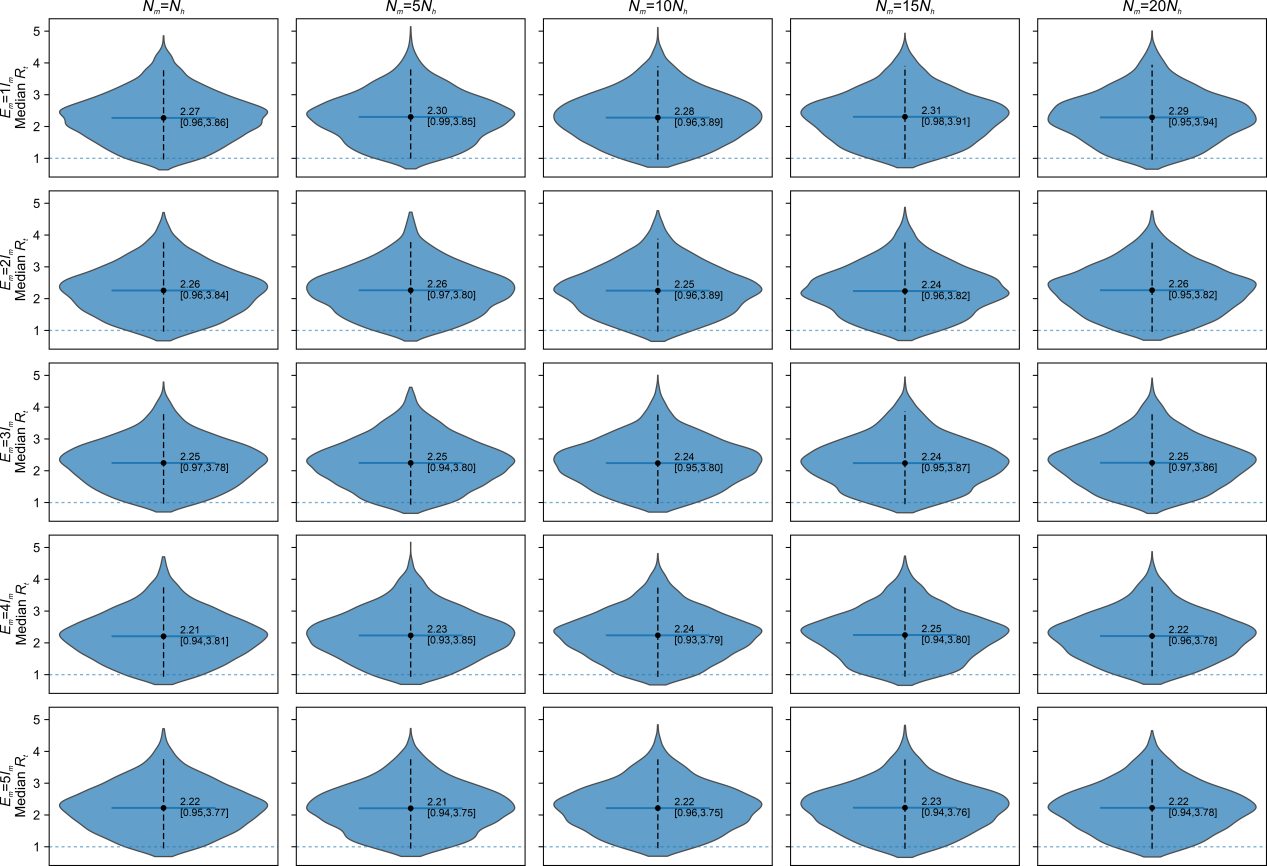
**

**Fig. 5.11 Monte Carlo simulation results of the median *R_t_* during the rising period for all scenarios in LC, 2023.**

**
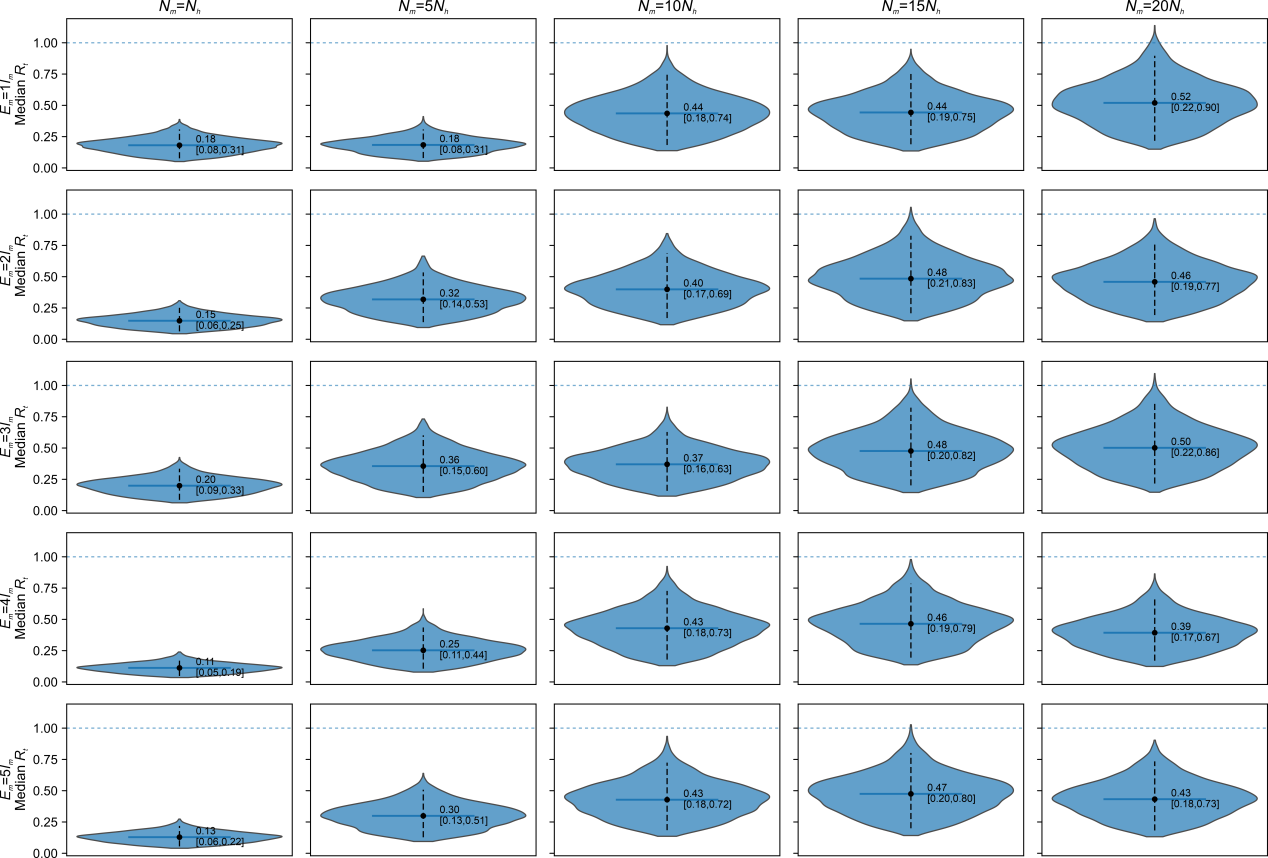
**

**Fig. 5.12 Monte Carlo simulation results of the median *R_t_* during the decline period for all scenarios in LC, 2023.**

## 5.2 *R_t_*_(_*_hm_*_)_ results


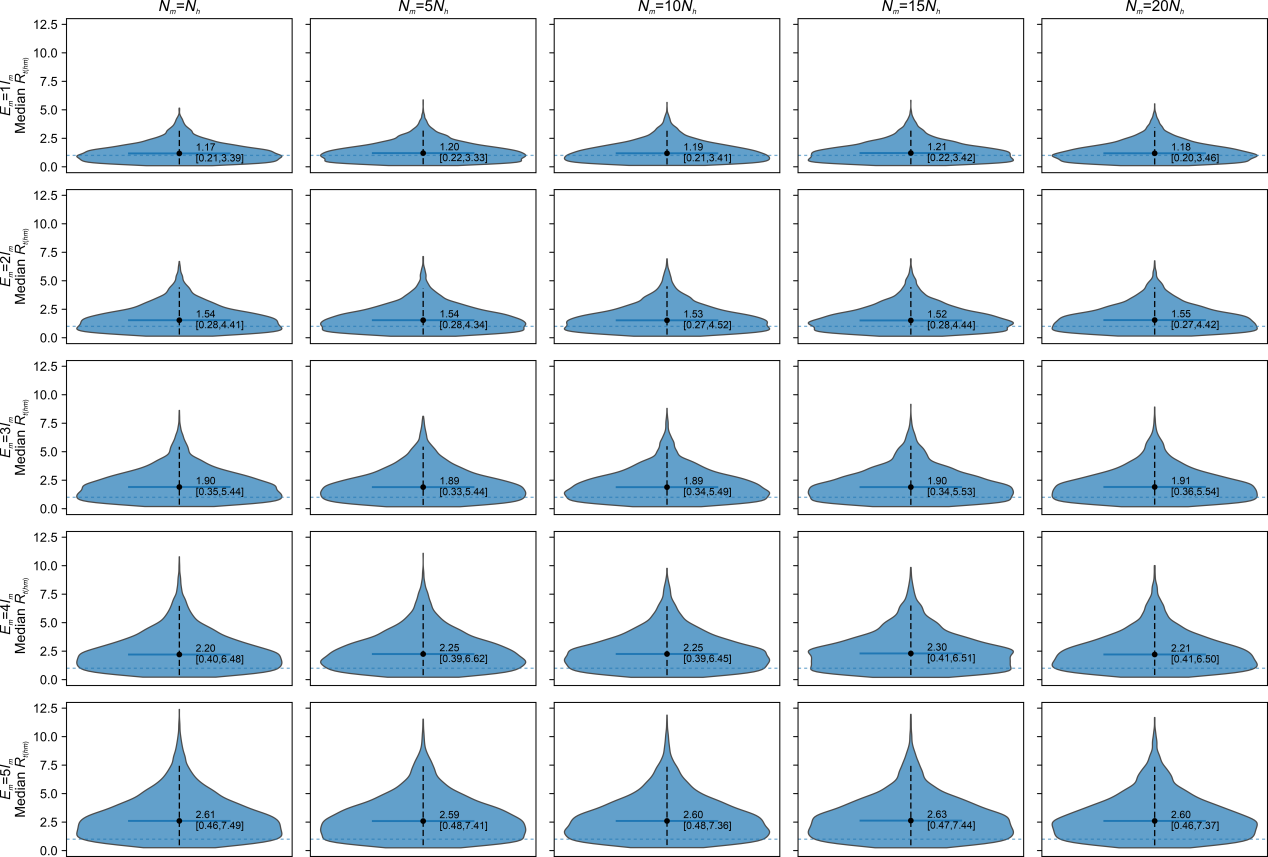


**Fig. 5.13 Monte Carlo simulation results of the median *R_t_*_(_*_hm_***_)_ **during the rising period for all scenarios in DH, 2017.**

**
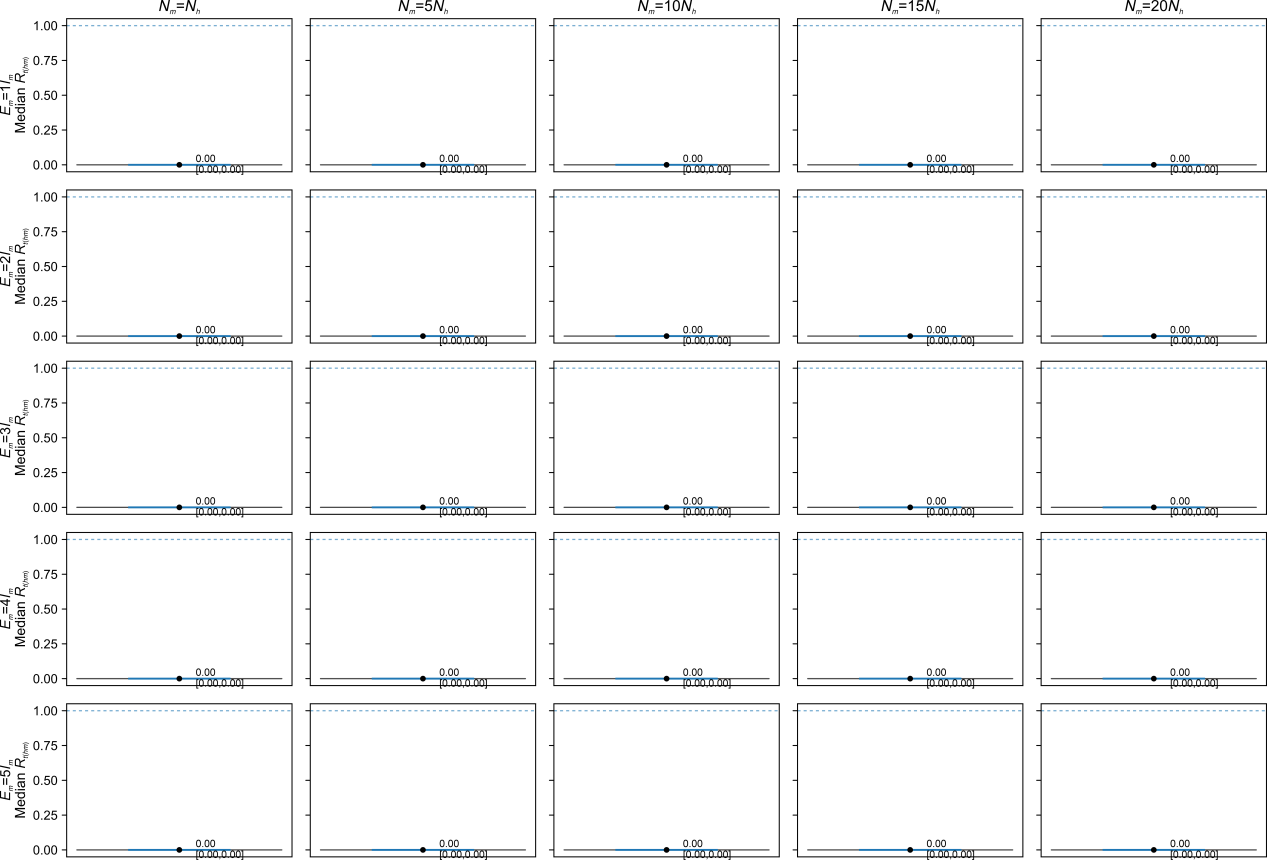
**

**Fig. 5.14 Monte Carlo simulation results of the median *R_t_*_(_*_hm_***_)_ **during the decline period for all scenarios in DH, 2017.**

**
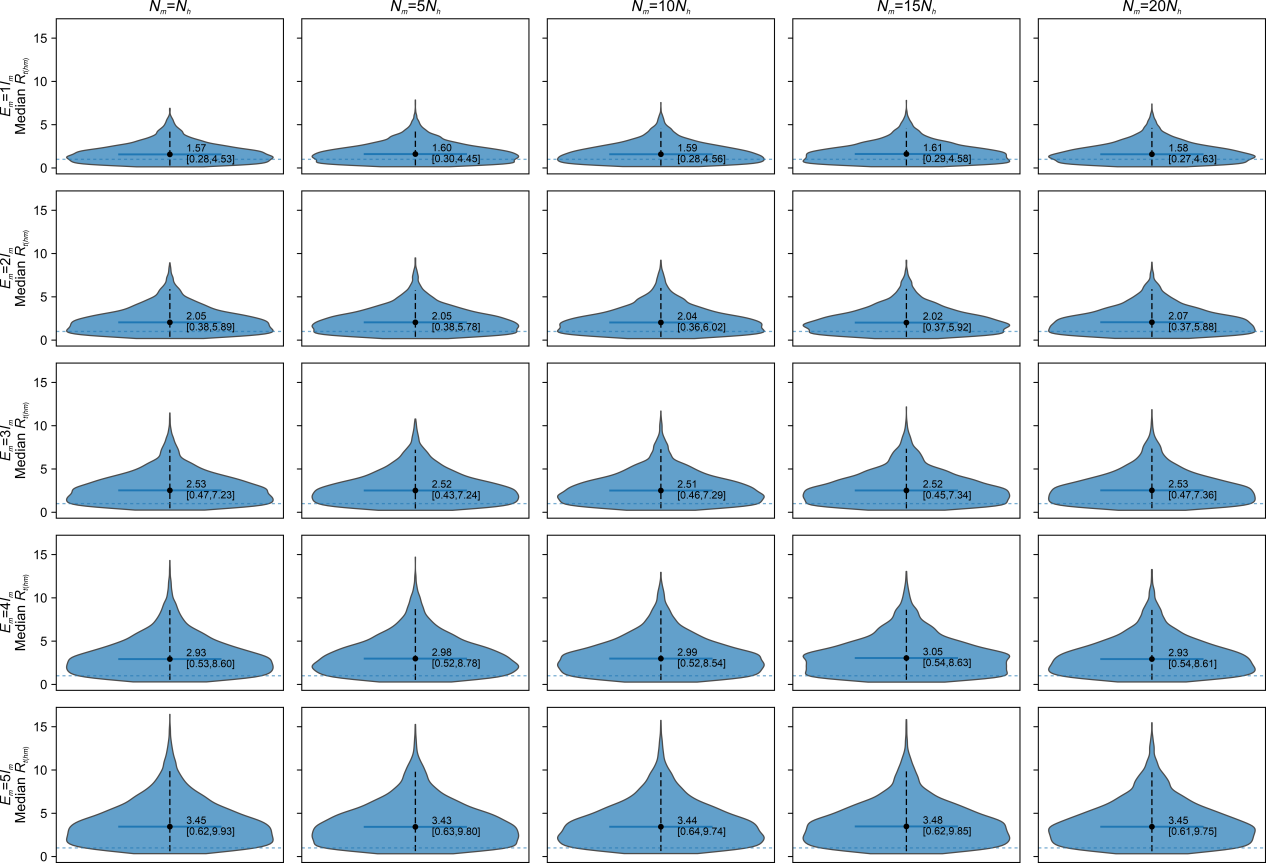
**

**Fig. 5.15 Monte Carlo simulation results of the median *R_t_*_(_*_hm_***_)_ **during the rising period for all scenarios in DH, 2019.**

**
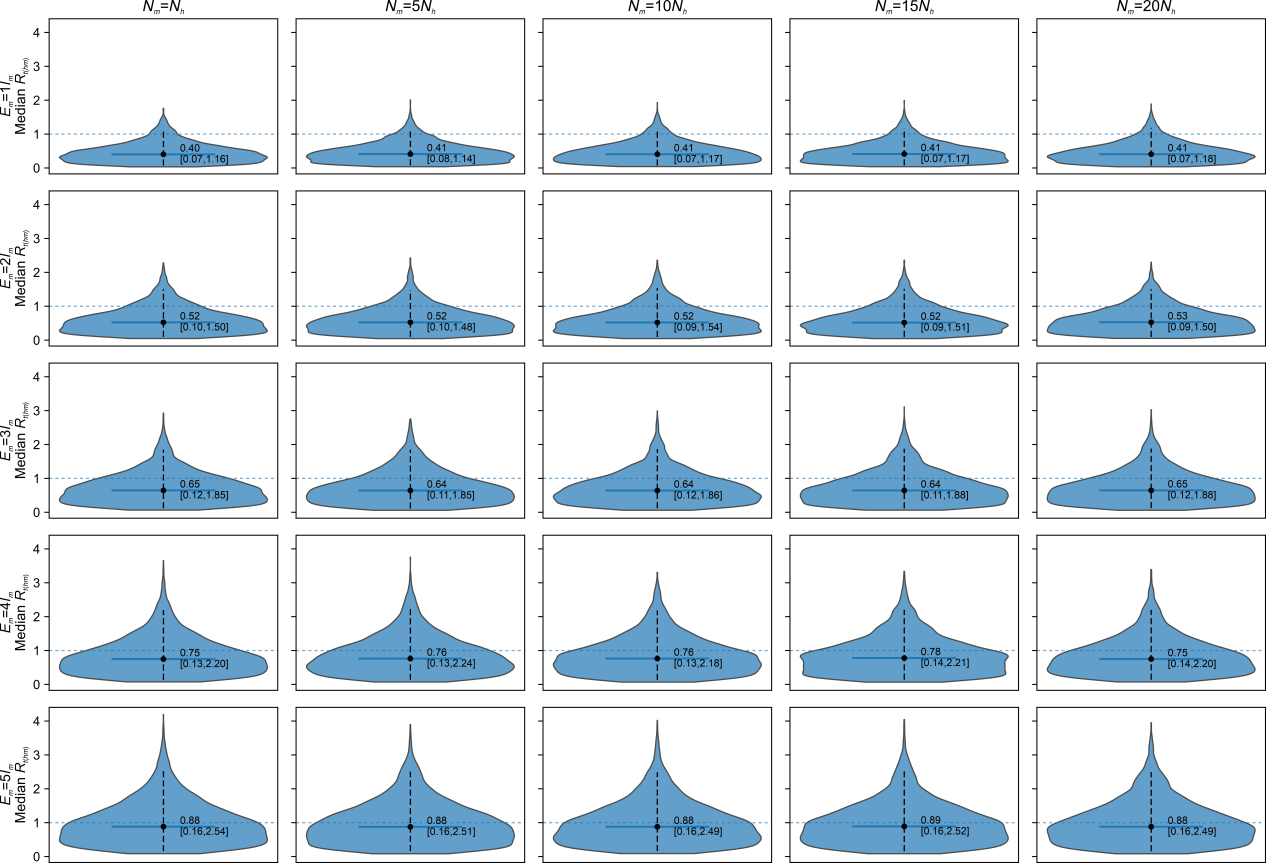
**

**Fig. 5.16 Monte Carlo simulation results of the median *R_t_*_(_*_hm_***_)_ **during the decline period for all scenarios in DH, 2019.**

**
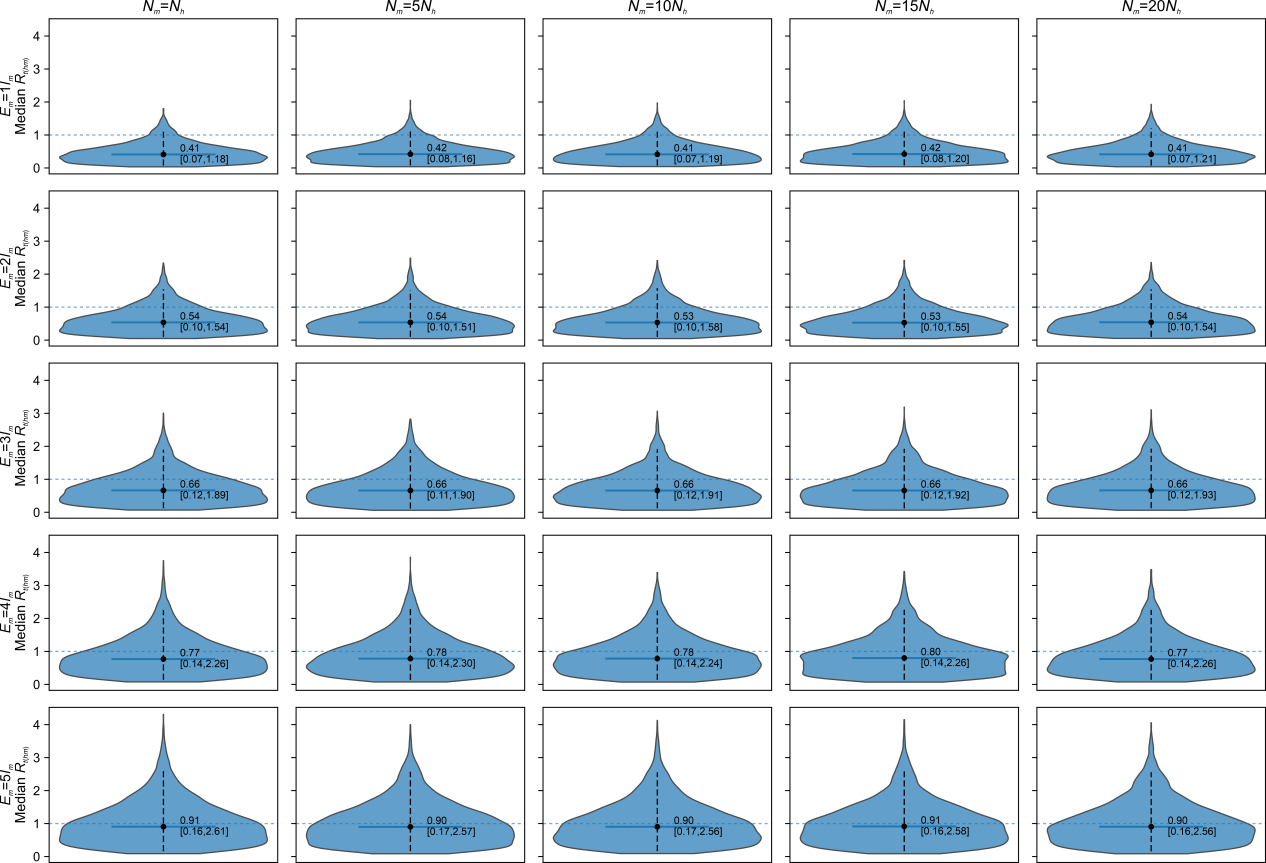
**

**Fig. 5.17 Monte Carlo simulation results of the median *R_t_*_(_*_hm_***_)_ **during the rising period for all scenarios in DH, 2023.**

**
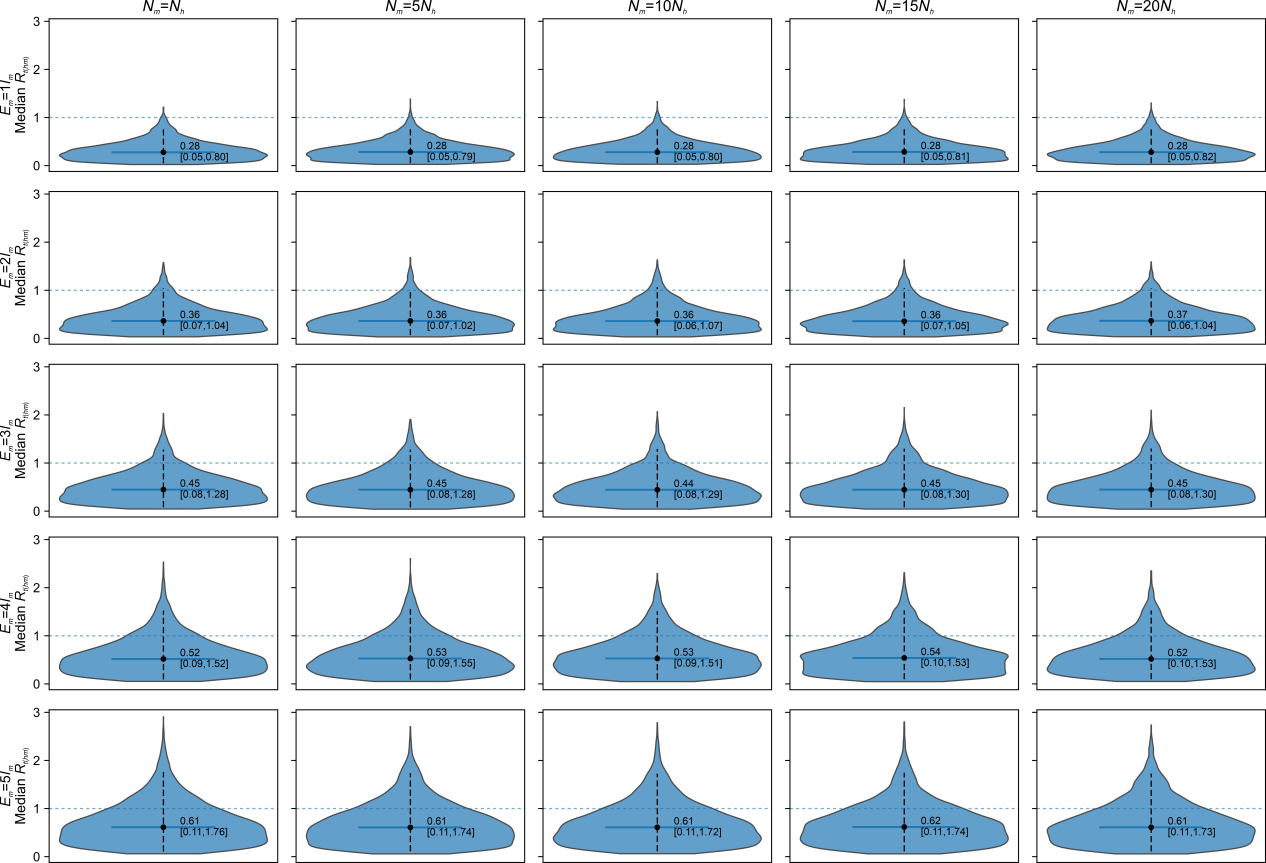
**

**Fig. 5.18 Monte Carlo simulation results of the median *R_t_*_(_*_hm_***_)_ **during the decline period for all scenarios in DH, 2023.**

**
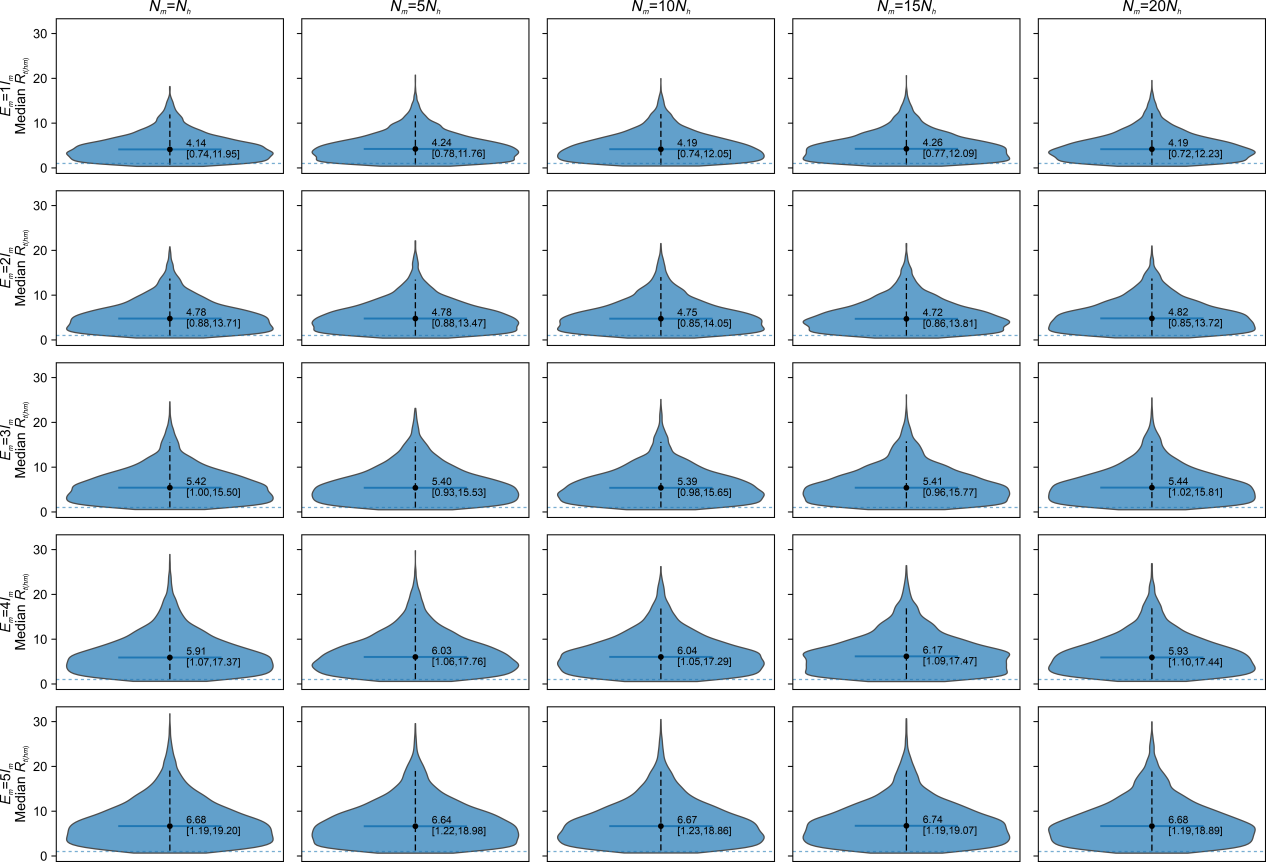
**

**Fig. 5.19 Monte Carlo simulation results of the median *R_t_*_(_*_hm_***_)_ **during the rising period for all scenarios in LC, 2017.**

**
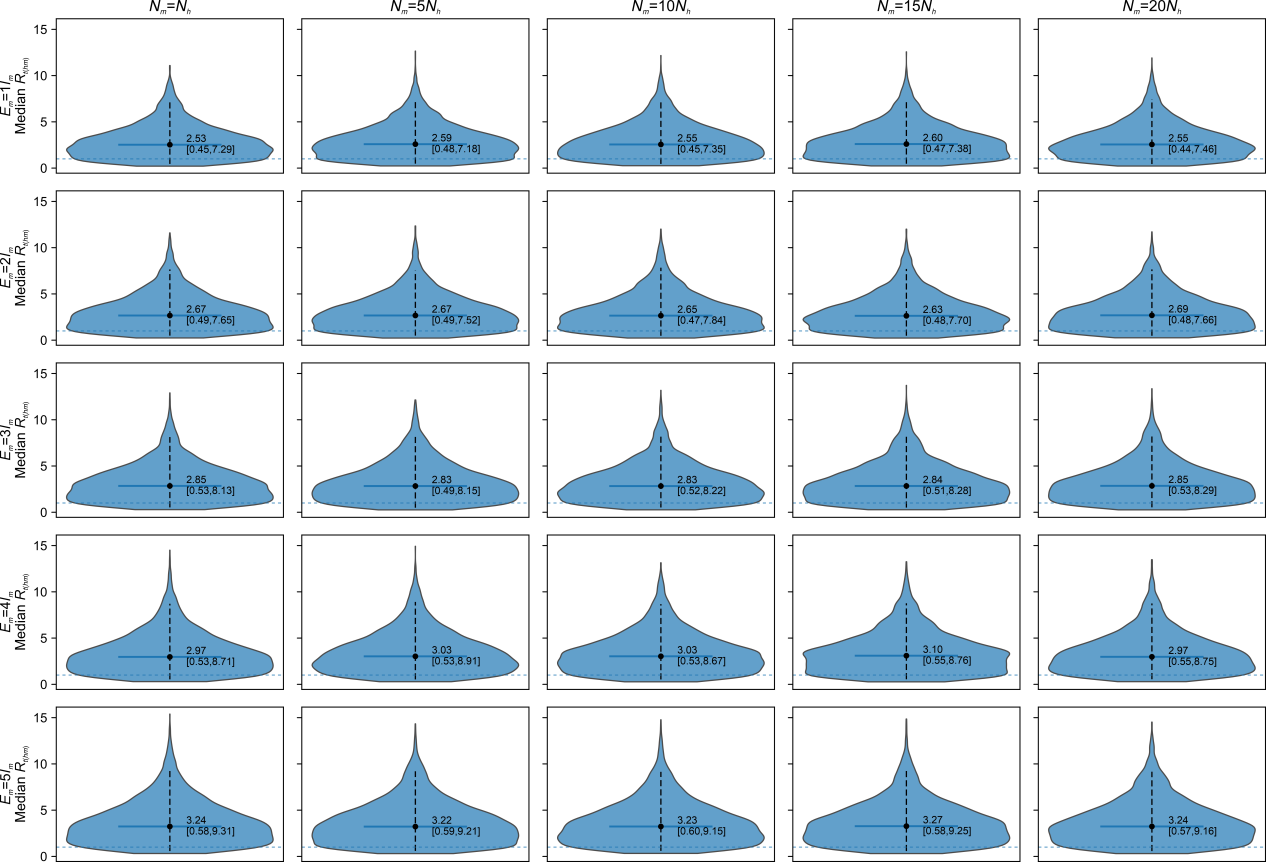
**

**Fig. 5.20 Monte Carlo simulation results of the median *R_t_*_(_*_hm_***_)_ **during the decline period for all scenarios in LC, 2017.**

**
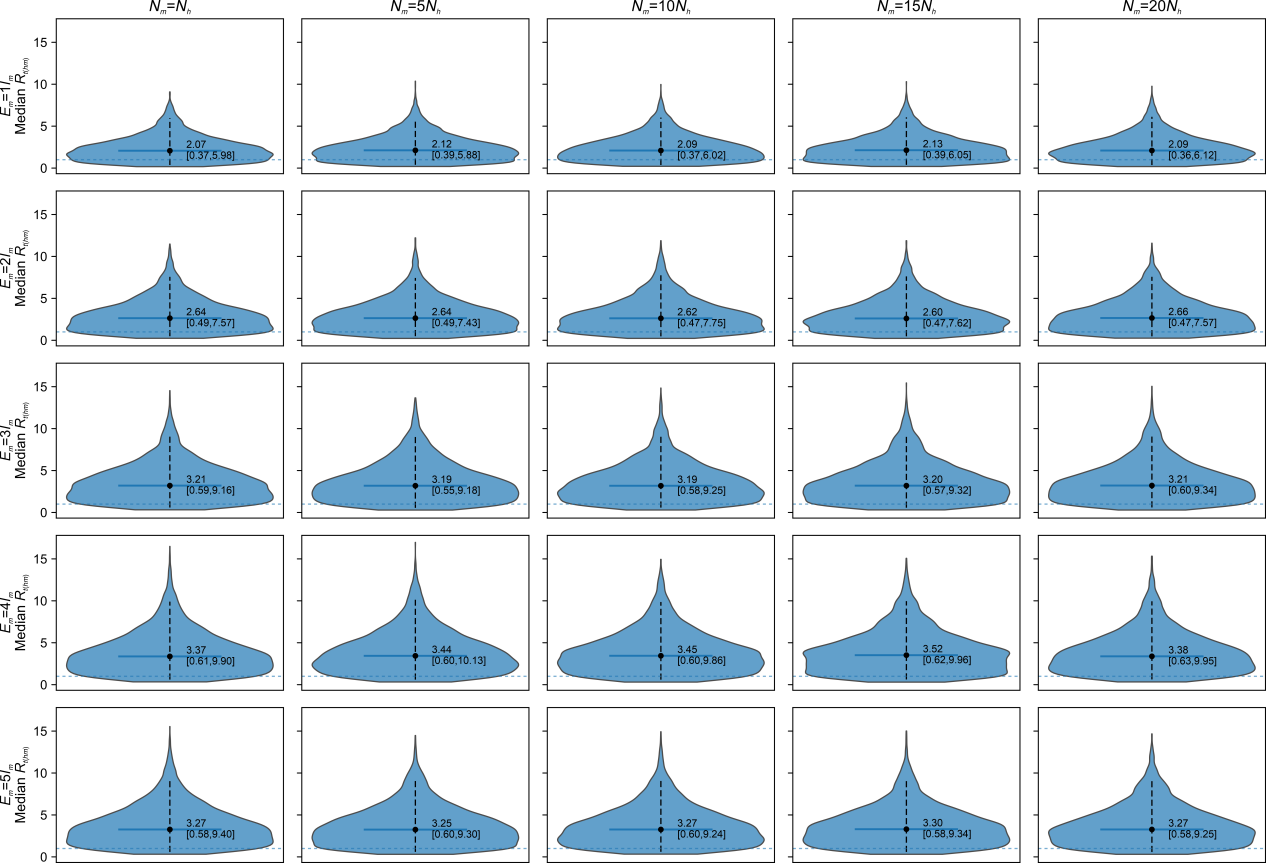
**

**Fig. 5.21 Monte Carlo simulation results of the median *R_t_*_(_*_hm_***_)_ **during the rising period for all scenarios in LC, 2019.**

**
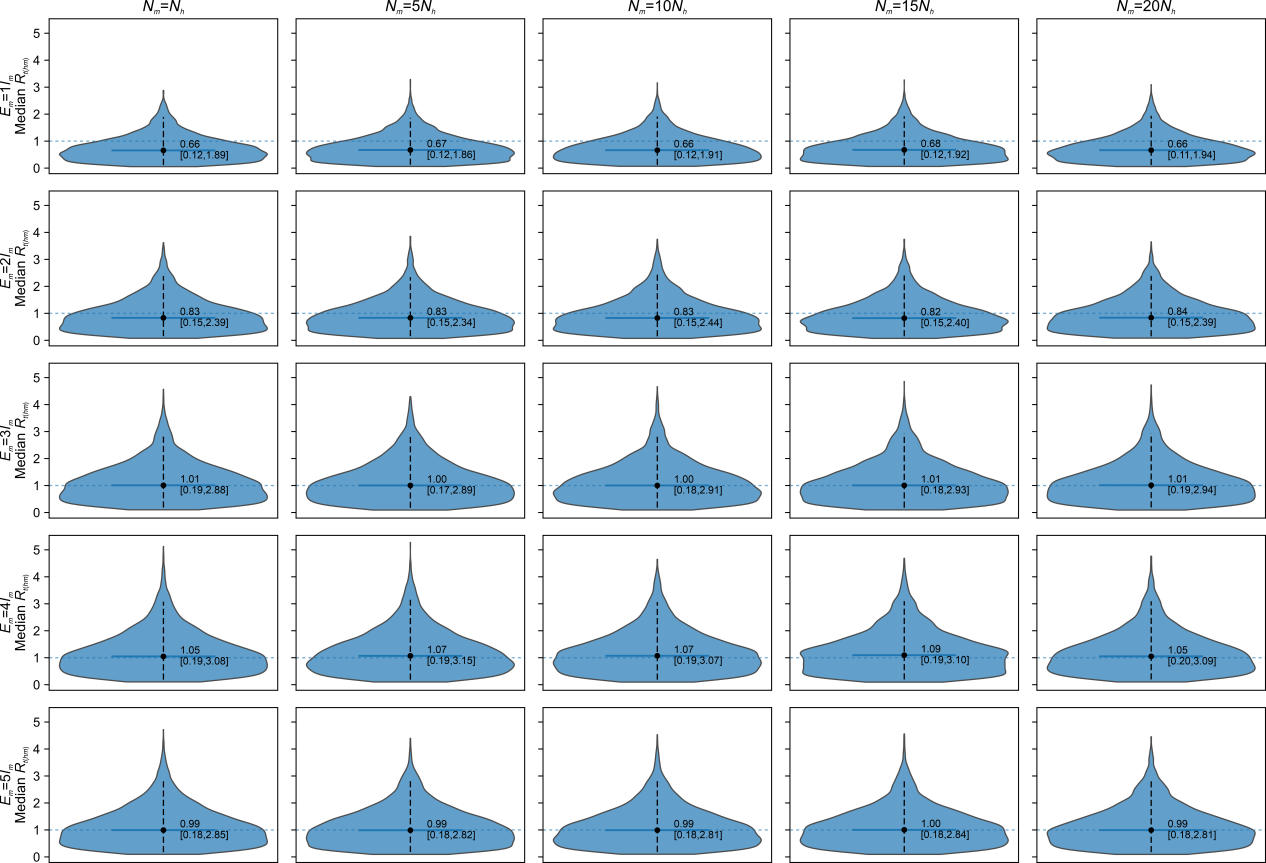
**

**Fig. 5.22 Monte Carlo simulation results of the median *R_t_*_(_*_hm_***_)_ **during the decline period for all scenarios in LC, 2019.**

**
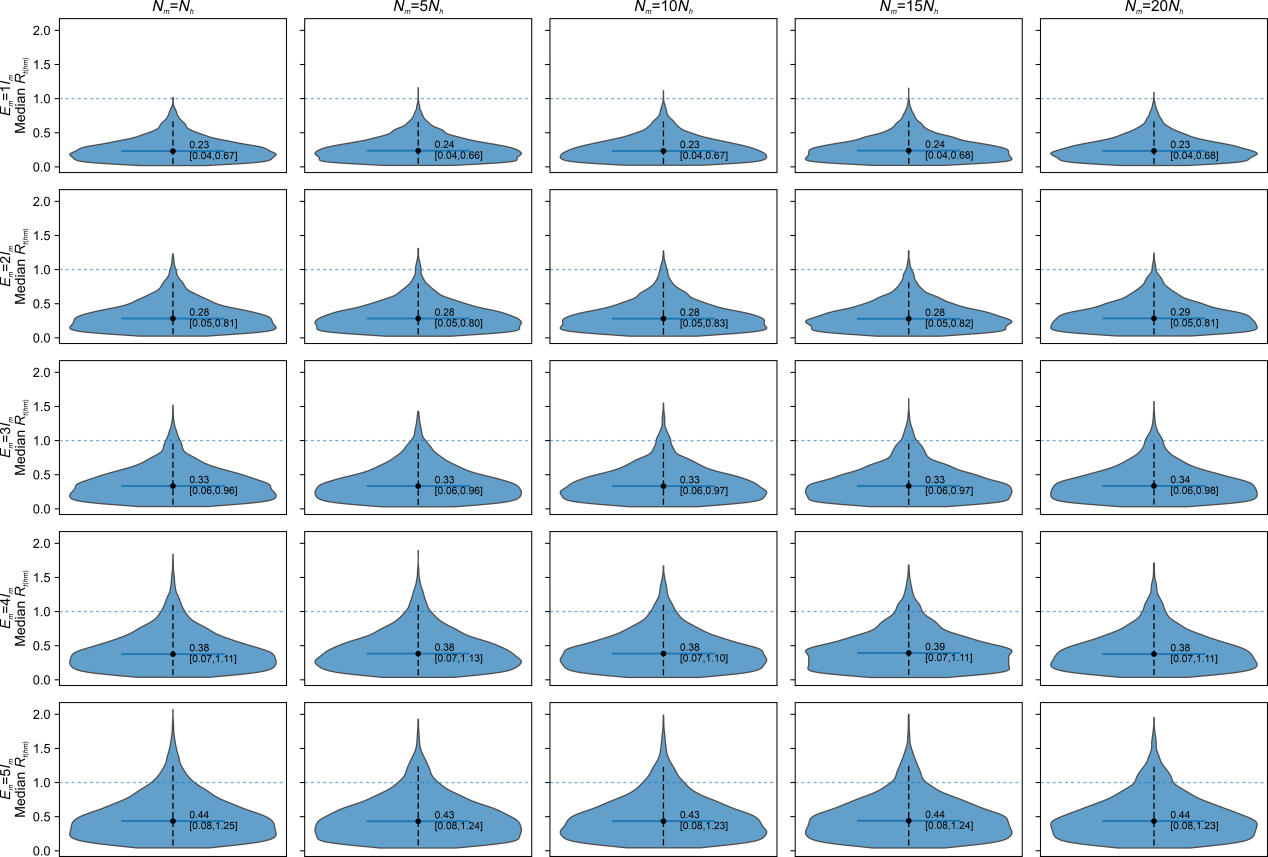
**

**Fig. 5.23 Monte Carlo simulation results of the median *R_t_*_(_*_hm_***_)_ **during the rising period for all scenarios in LC, 2023.**

**
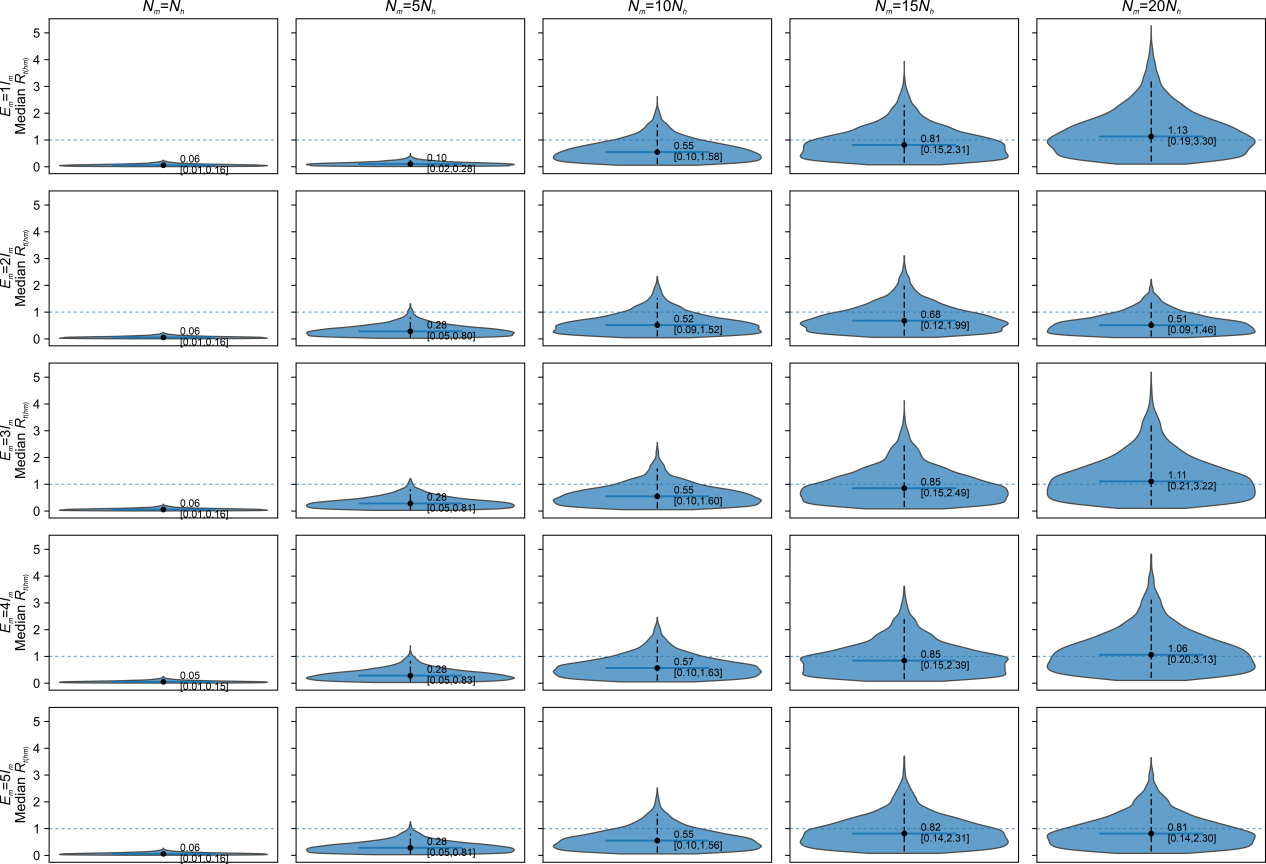
**

**Fig. 5.24 Monte Carlo simulation results of the median *R_t_*_(_*_hm_***_)_ **during the decline period for all scenarios in LC, 2023.**

## 5.3 *R_t_*_(_*_mh_*_)_ results


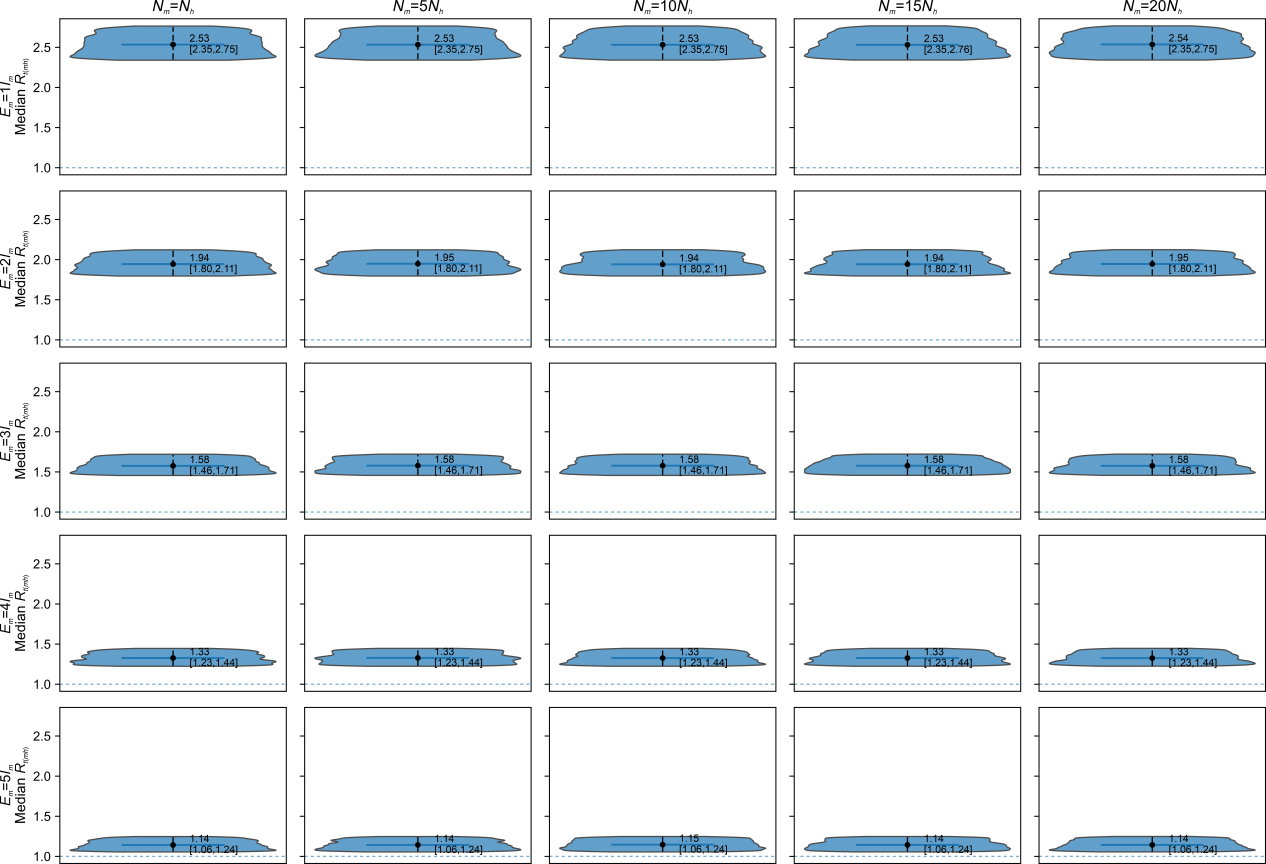


**Fig. 5.25 Monte Carlo simulation results of *R_t_*_(_*_mh_***_)_ **during the rising period for all scenarios in DH, 2017.**

**
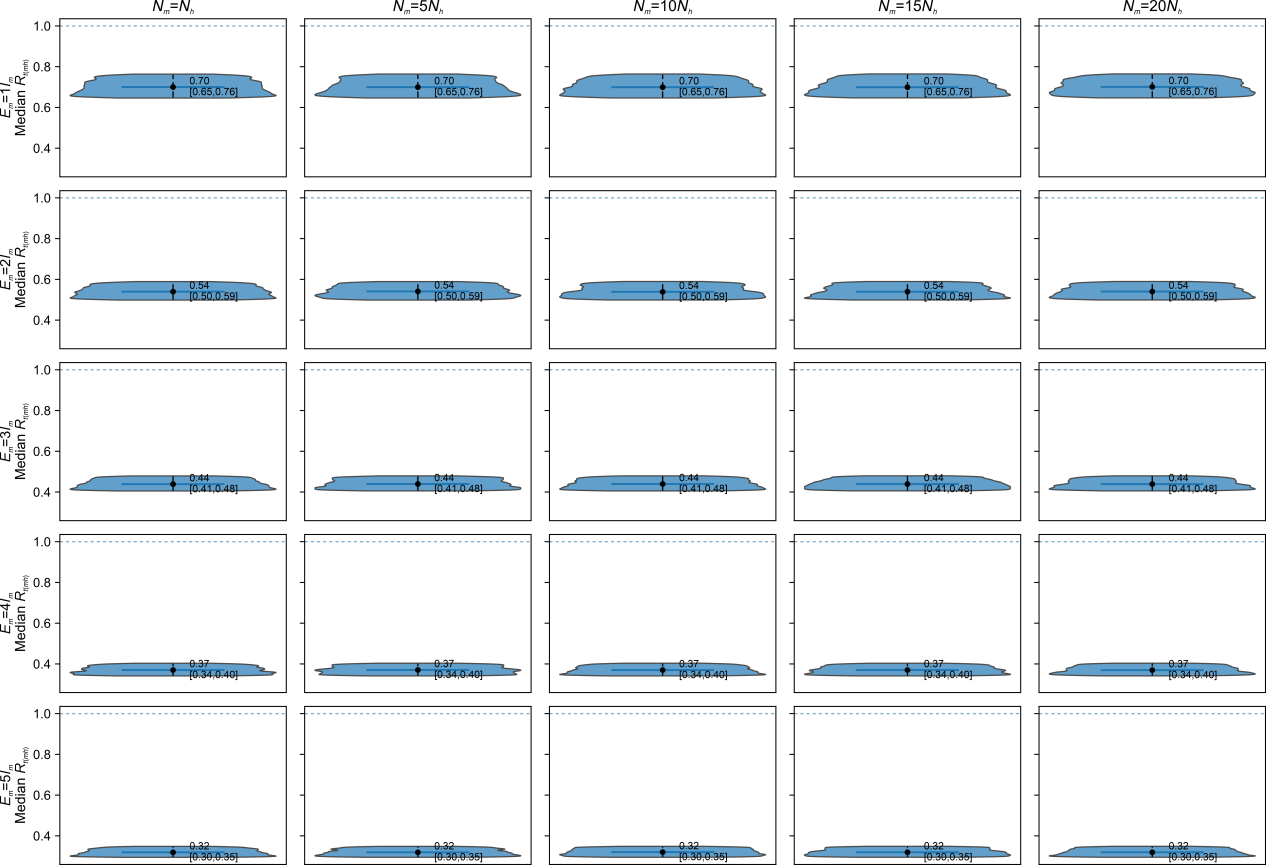
**

**Fig. 5.26 Monte Carlo simulation results of *R_t_*_(_*_mh_***_)_ **during the decline period for all scenarios in DH, 2017.**

**
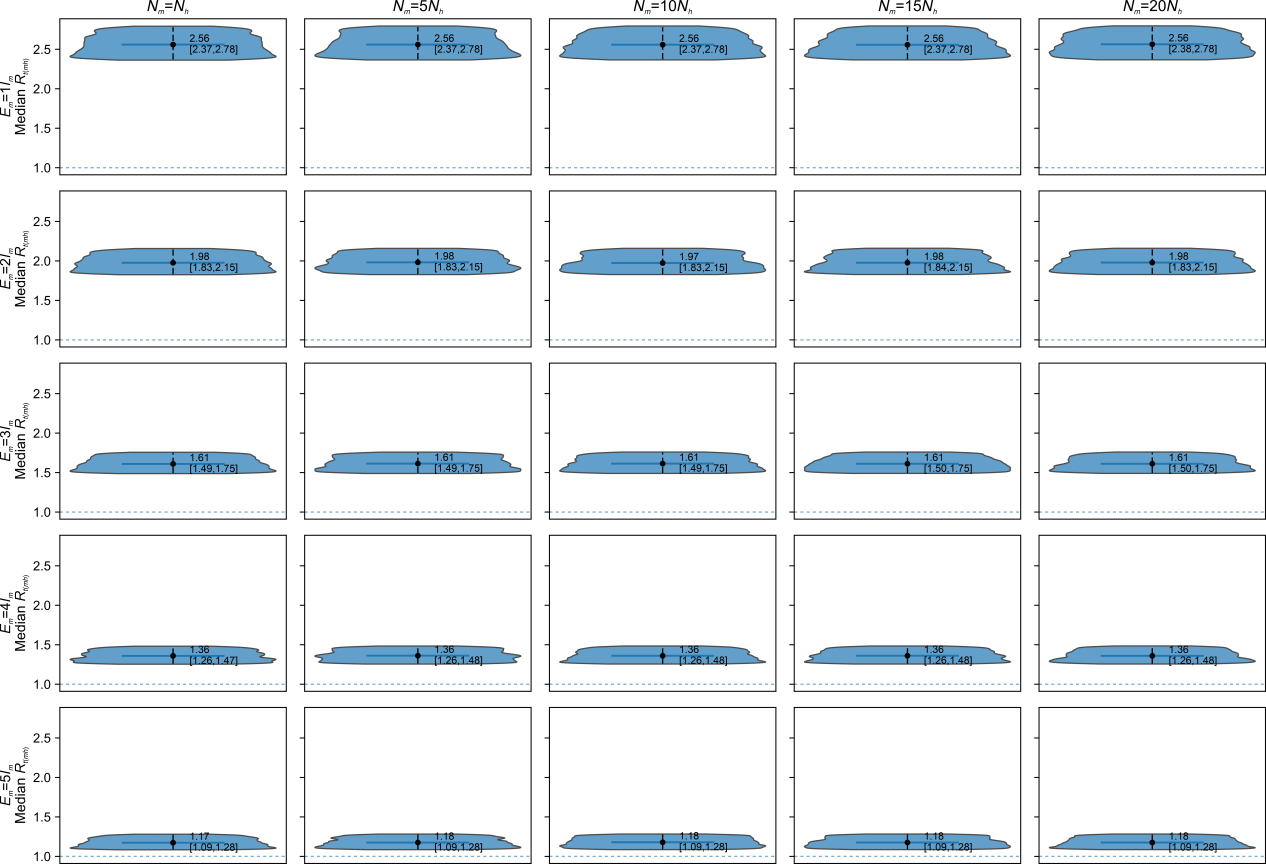
**

**Fig. 5.27 Monte Carlo simulation results of *R_t_*_(_*_mh_***_)_ **during the rising period for all scenarios in DH, 2019.**

**
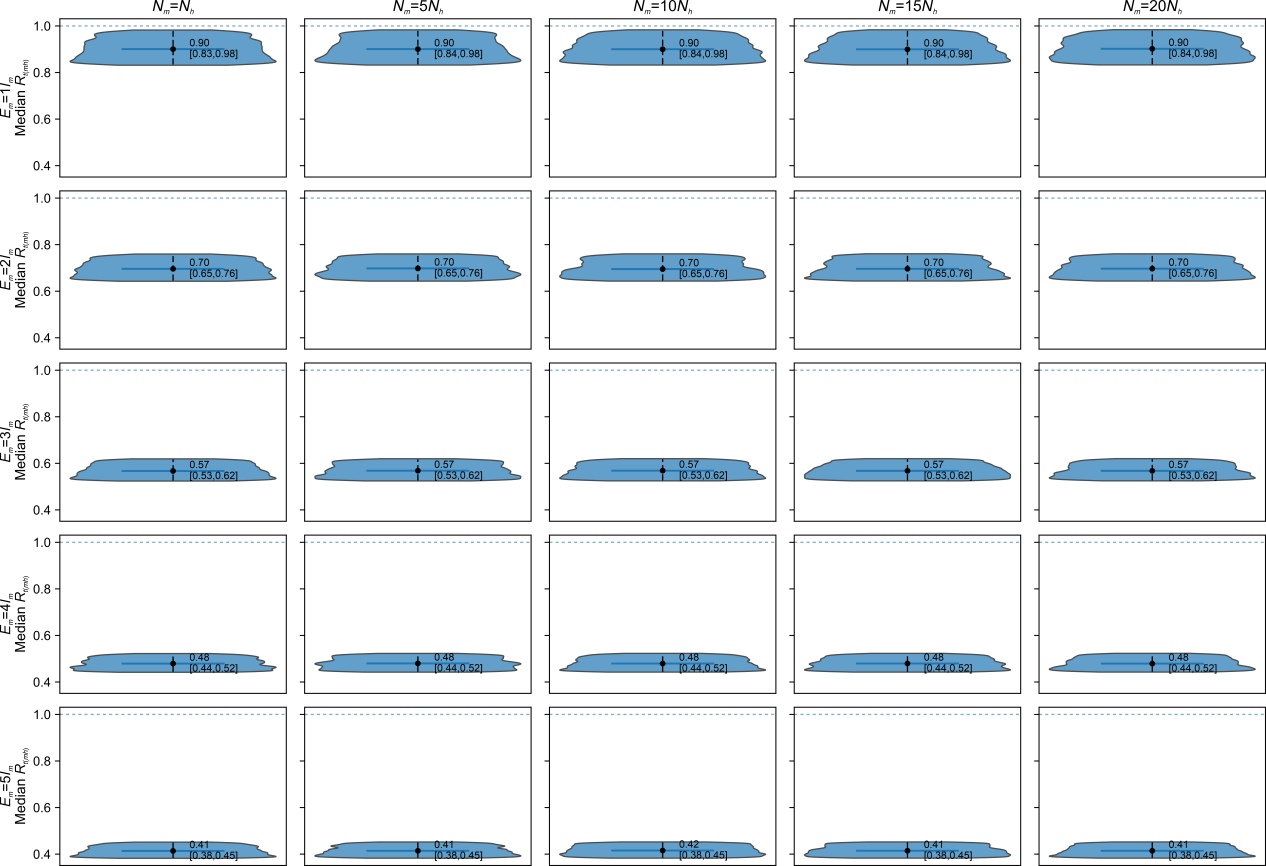
**

**Fig. 5.28 Monte Carlo simulation results of *R_t_*_(_*_mh_***_)_ **during the decline period for all scenarios in DH, 2019.**

**
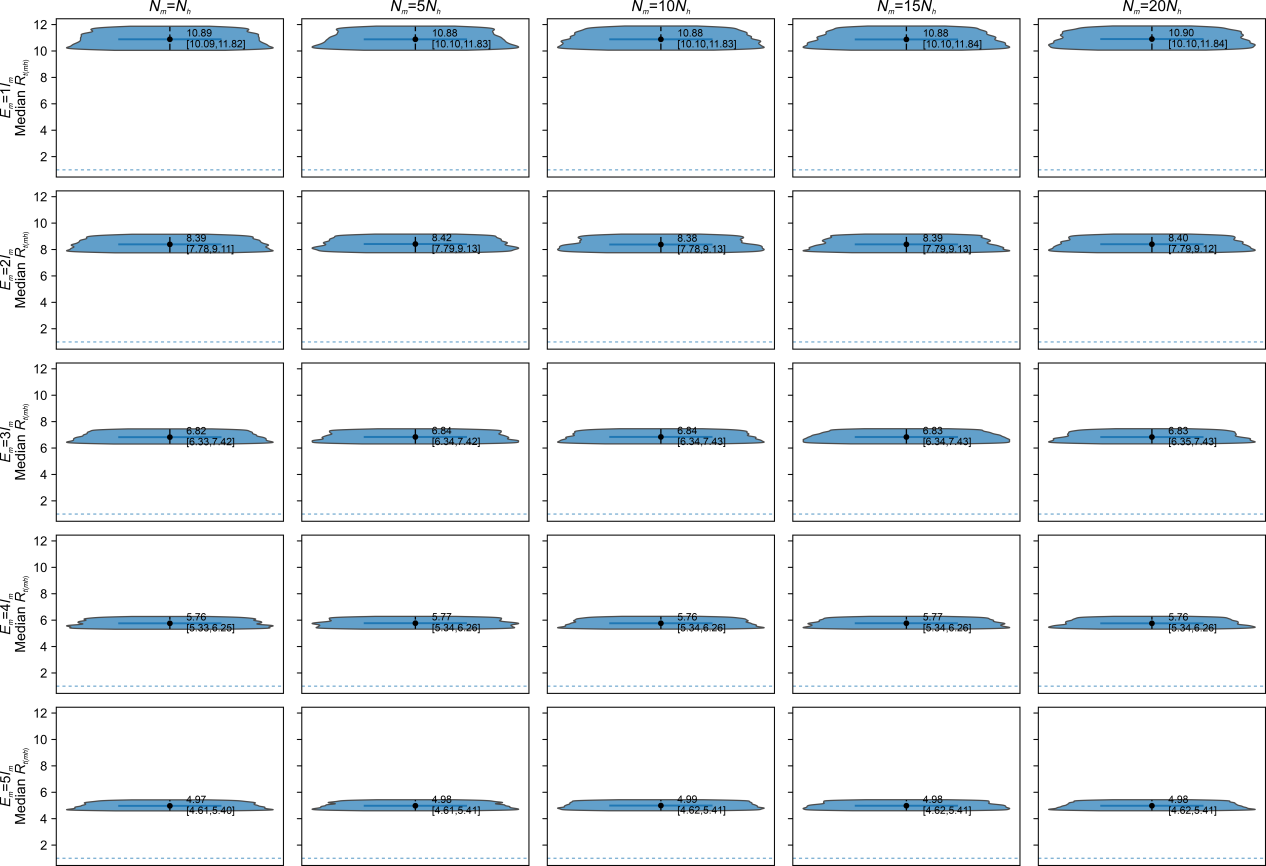
**

**Fig. 5.29 Monte Carlo simulation results of *R_t_*_(_*_mh_***_)_ **during the rising period for all scenarios in DH, 2023.**

**
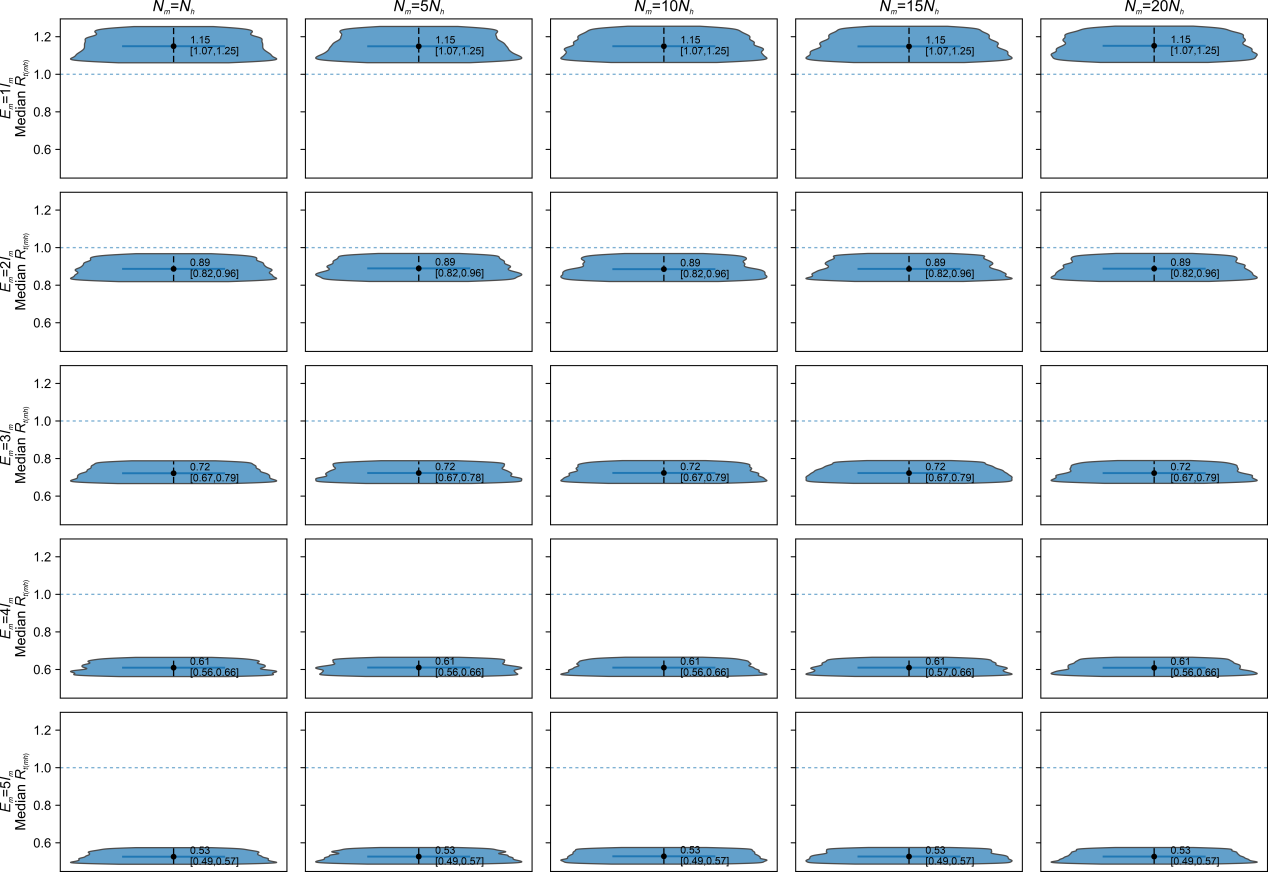
**

**Fig. 5.30 Monte Carlo simulation results of *R_t_*_(_*_mh_***_)_ **during the decline period for all scenarios in DH, 2023.**

**
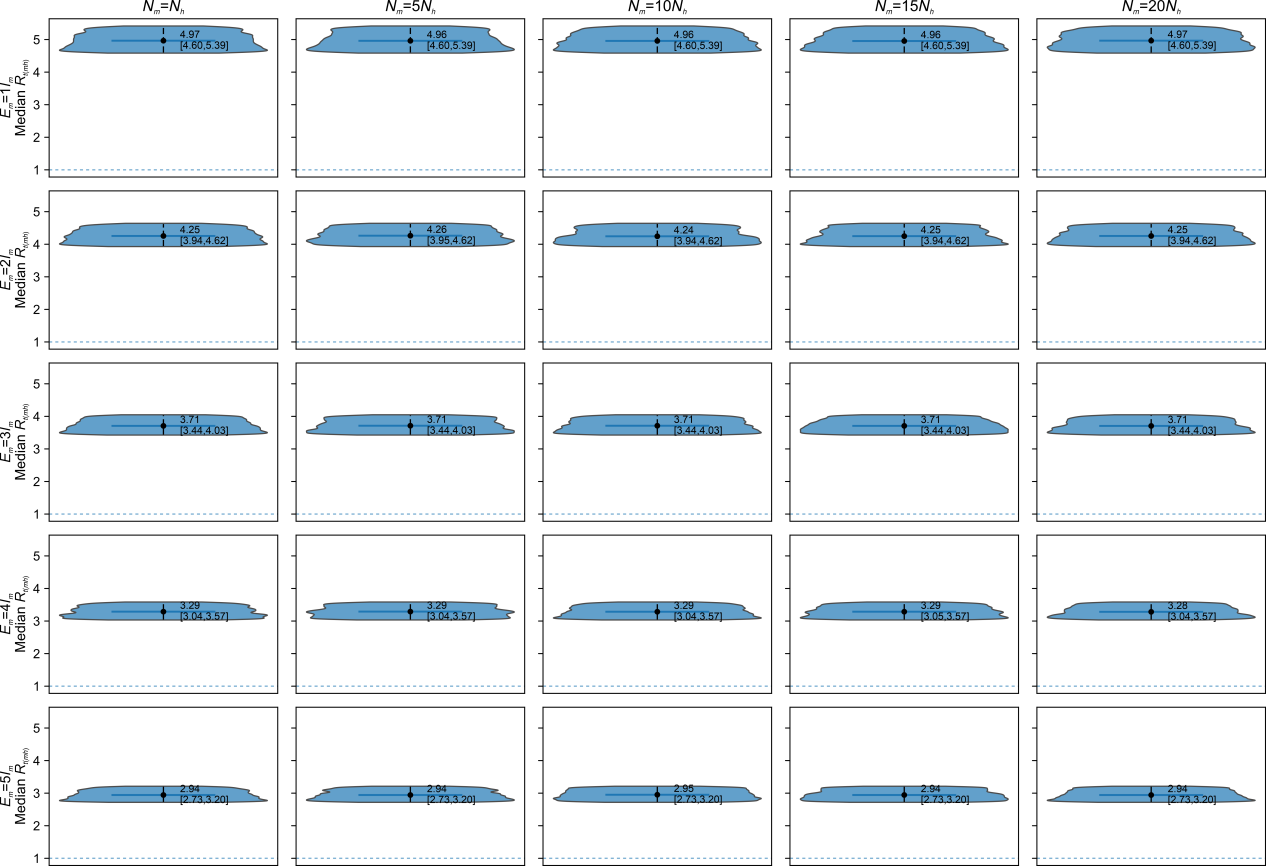
**

**Fig. 5.31 Monte Carlo simulation results of *R_t_*_(_*_mh_***_)_ **during the rising period for all scenarios in LC, 2017.**

**
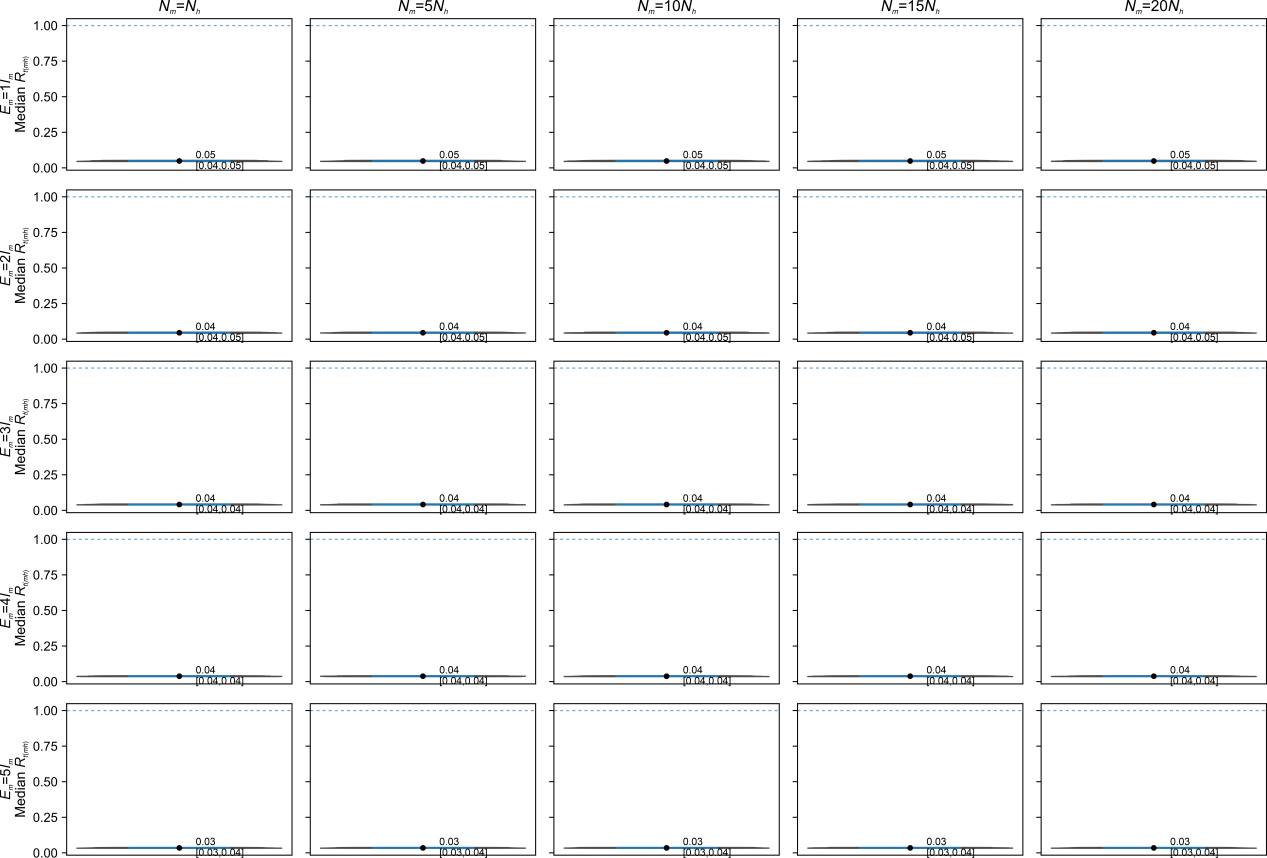
**

**Fig. 5.32 Monte Carlo simulation results of *R_t_*_(_*_mh_***_)_ **during the decline period for all scenarios in LC, 2017.**

**
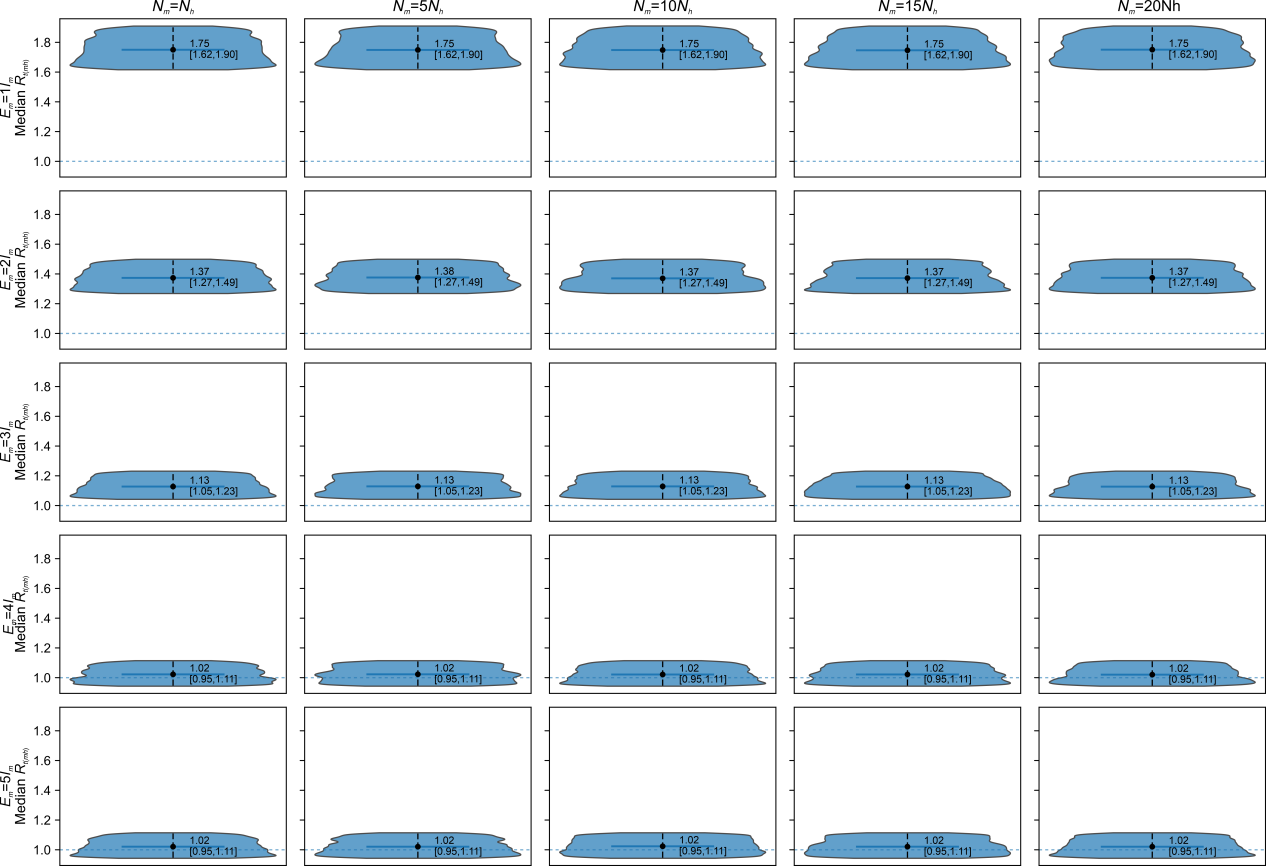
**

**Fig. 5.33 Monte Carlo simulation results of *R_t_*_(_*_mh_***_)_ **during the rising period for all scenarios in LC, 2019.**

**
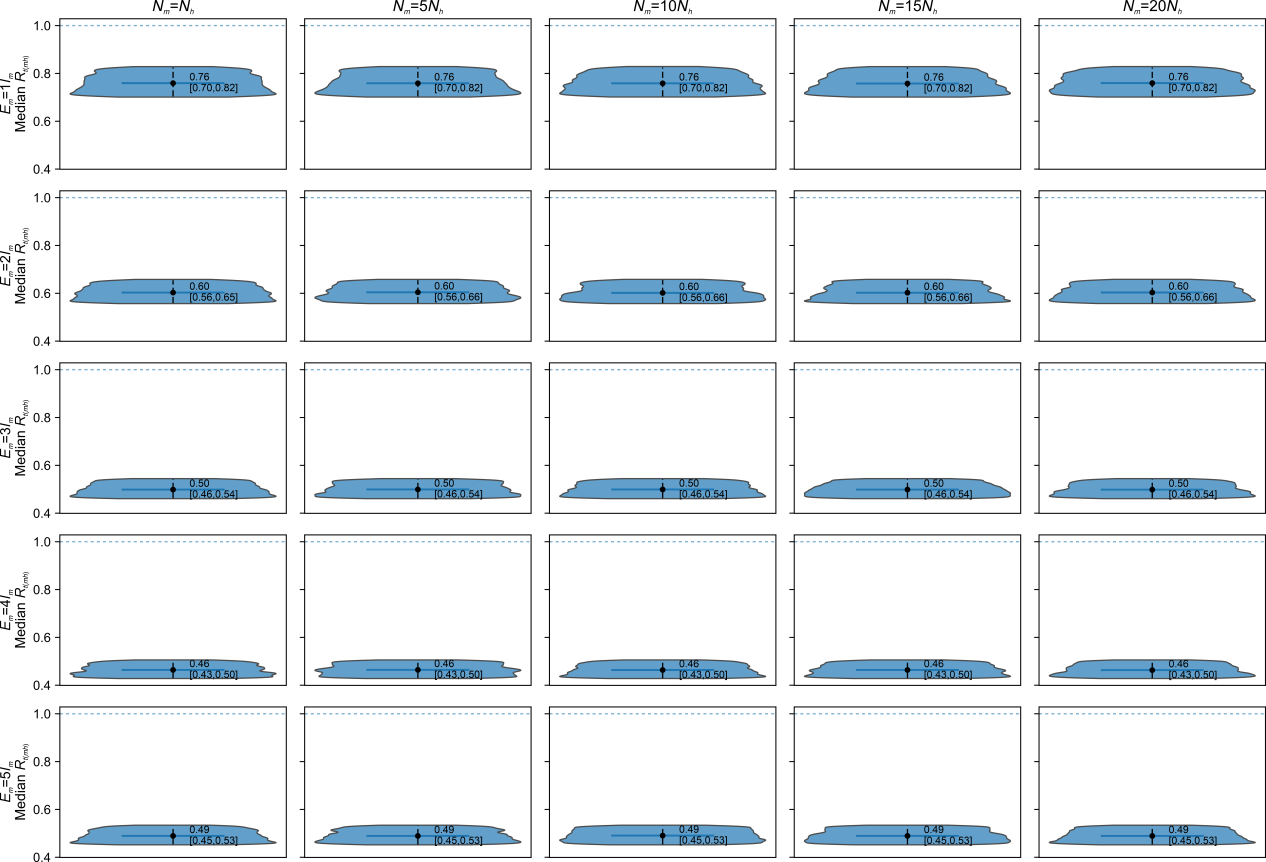
**

**Fig. 5.34 Monte Carlo simulation results of *R_t_*_(_*_mh_***_)_ **during the decline period for all scenarios in LC, 2019.**

**
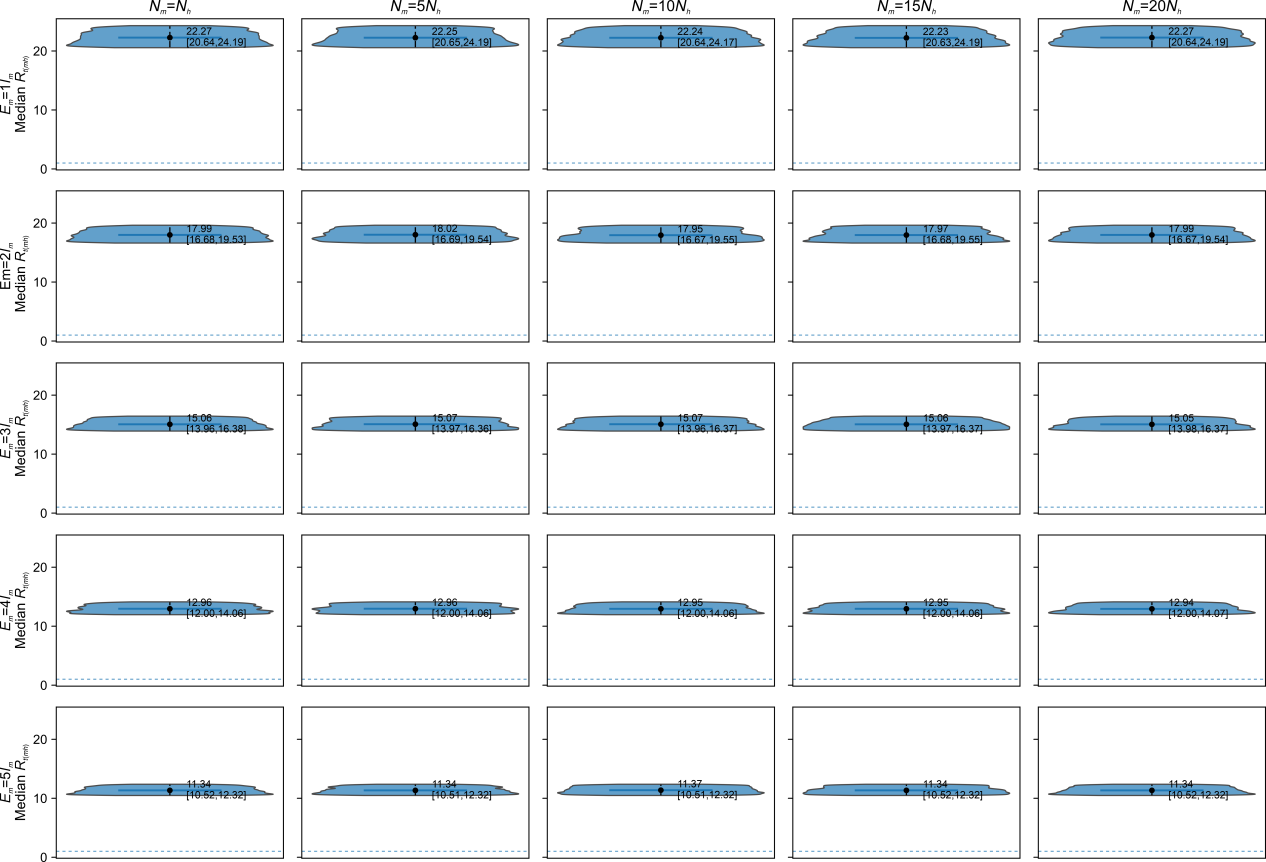
**

**Fig. 5.35 Monte Carlo simulation results of *R_t_*_(_*_mh_***_)_ **during the rising period for all scenarios in LC, 2023.**

**
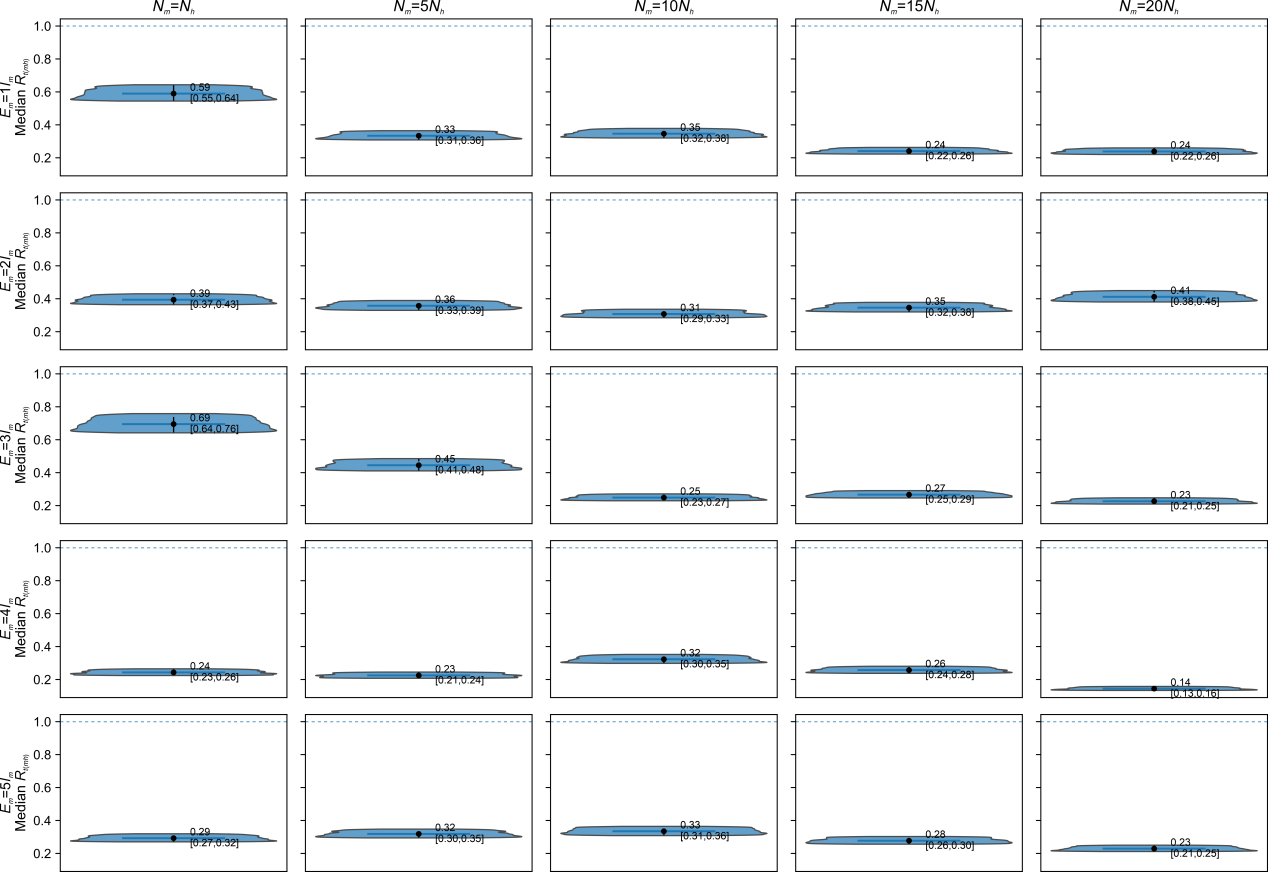
**

**Fig. 5.36 Monte Carlo simulation results of *R_t_*_(_*_mh_***_)_ **during the decline period for all scenarios in LC, 2023.**
